# Grad‐seq in a Gram‐positive bacterium reveals exonucleolytic sRNA activation in competence control

**DOI:** 10.15252/embj.2019103852

**Published:** 2020-03-30

**Authors:** Jens Hör, Geneviève Garriss, Silvia Di Giorgio, Lisa‐Marie Hack, Jens T Vanselow, Konrad U Förstner, Andreas Schlosser, Birgitta Henriques‐Normark, Jörg Vogel

**Affiliations:** ^1^ Institute of Molecular Infection Biology University of Würzburg Würzburg Germany; ^2^ Department of Microbiology, Tumor & Cell Biology Karolinska Institutet Stockholm Sweden; ^3^ ZB MED—Information Centre for Life Sciences Cologne Germany; ^4^ Helmholtz Institute for RNA‐based Infection Research (HIRI) Helmholtz Centre for Infection Research (HZI) Würzburg Germany; ^5^ Rudolf Virchow Center for Experimental Biomedicine University of Würzburg Würzburg Germany; ^6^ Faculty of Information Science and Communication Studies TH Köln Cologne Germany; ^7^ Department of Clinical Microbiology Karolinska University Hospital Stockholm Sweden; ^8^ SCELSE and LKC Nanyang Technological University, NTU Singapore Singapore

**Keywords:** Cbf1, competence, Grad‐seq, RNA–protein complex, *Streptococcus pneumoniae*, Microbiology, Virology & Host Pathogen Interaction, RNA Biology

## Abstract

RNA–protein interactions are the crucial basis for many steps of bacterial gene expression, including post‐transcriptional control by small regulatory RNAs (sRNAs). In stark contrast to recent progress in the analysis of Gram‐negative bacteria, knowledge about RNA–protein complexes in Gram‐positive species remains scarce. Here, we used the Grad‐seq approach to draft a comprehensive landscape of such complexes in *Streptococcus pneumoniae*, in total determining the sedimentation profiles of ~ 88% of the transcripts and ~ 62% of the proteins of this important human pathogen. Analysis of in‐gradient distributions and subsequent tag‐based protein capture identified interactions of the exoribonuclease Cbf1/YhaM with sRNAs that control bacterial competence for DNA uptake. Unexpectedly, the nucleolytic activity of Cbf1 stabilizes these sRNAs, thereby promoting their function as repressors of competence. Overall, these results provide the first RNA/protein complexome resource of a Gram‐positive species and illustrate how this can be utilized to identify new molecular factors with functions in RNA‐based regulation of virulence‐relevant pathways.

## Graphical abstract

Comprehensive assessment of protein‐RNA complexes in *Streptococcus pneumoniae* via Grad‐seq uncovers an unexpected role for the exoribonuclease Cbf1 in stabilizing sRNAs that control bacterial competence for DNA uptake.

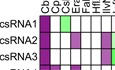

## Introduction

Within the past two decades, our view of bacterial gene regulation has dramatically changed. Once considered as organisms with a protein output that is a straight function of transcription initiation, we know now that bacteria amply use noncoding RNAs (ncRNAs) and RNA‐binding proteins (RBPs) in post‐transcriptional control networks that impact almost every aspect of physiology (Storz & Papenfort, 2019). Interactions with cellular proteins crucially underlie many of these RNA functions, running the gamut of universal ribonucleoprotein particles (RNPs) with housekeeping functions, 6S RNA‐mediated modulation of RNA polymerase (RNAP), CRISPR/Cas complexes for genome defense, and an increasing number of small regulatory RNAs (sRNAs) that associate with global RNA‐binding proteins (RBPs) (Holmqvist & Vogel, 2018).

Much of this recent progress has come from studies in Gram‐negative bacteria, especially *Escherichia coli* and *Salmonella enterica* (Hör *et al*, 2018, 2020). These model bacteria express at least three global RBPs—CsrA, Hfq, and ProQ—that form complexes with hundreds of different sRNAs to facilitate extensive post‐transcriptional control of mRNAs (Olejniczak & Storz, 2017; Holmqvist & Vogel, 2018; Kavita *et al*, 2018; Babitzke *et al*, 2019). The situation is very different with Gram‐positive bacteria: Despite much evidence that sRNAs also play important roles in these organisms (Brantl & Brückner, 2014; Wagner & Romby, 2015; Quereda & Cossart, 2017; Wassarman, 2018; Desgranges *et al*, 2019), many aspects of their sRNA biology seem to differ from Gram‐negative species. For example, CsrA circuits work without antagonistic sRNAs, Hfq seems to play a minor role, ProQ is generally absent, and a major sRNA‐related RBP yet awaits to be discovered. Major ribonucleases, and hence RNA metabolism, also differ from Gram‐negative bacteria (Durand & Condon, 2018), and in more general terms, it is fair to say that our knowledge on RNA–protein interactions and molecular complexes is lagging behind in Gram‐positive species.

We have recently developed gradient profiling by sequencing (Grad‐seq) to accelerate the discovery of the major functional RNAs and RBPs in a bacterium of interest (Smirnov *et al*, 2016). This new type of complexomics approach predicts native cellular complexes by high‐throughput RNA and protein analysis of biochemically fractionated cellular lysates. In its pioneering application to *Salmonella* (Smirnov *et al*, 2016), Grad‐seq brought to light ProQ as a previously overlooked major RBP in enteric bacteria (Smirnov *et al*, 2017b; Holmqvist *et al*, 2018; Westermann *et al*, 2019; Melamed *et al*, 2020). Given the generic nature of the method, we reasoned that Grad‐seq would lend itself to guide the discovery of new RNA‐based mechanisms in Gram‐positive bacteria as well.

In the present work, we have applied Grad‐seq to *Streptococcus pneumoniae* to provide the first census of potential RNA and protein complexes in a Gram‐positive bacterium. The pneumococcus is a leading human pathogen, causing diverse infectious diseases such as otitis media, sinusitis, sepsis, meningitis, and pneumonia, and is responsible for more than one million deaths annually (O'Brien *et al*, 2009; Henriques‐Normark & Tuomanen, 2013). Unsurprisingly, pneumococcal transcriptomes, including condition‐specific sRNA expression, have been extensively characterized (Acebo *et al*, 2012; Mann *et al*, 2012; Aprianto *et al*, 2018; Slager *et al*, 2018, 2019; Warrier *et al*, 2018; Sinha *et al*, 2019) and potential binary protein–protein interactions have also been predicted (Wuchty *et al*, 2017). Although these studies yielded invaluable new insight into the molecular underpinnings of pneumococcal virulence, they did not cover functional RNA–protein interactions, especially for sRNAs with known roles in these processes. Here, the application of Grad‐seq allowed us to identify the sedimentation profiles of thousands of *S. pneumoniae* transcripts and proteins simultaneously in a single experiment. We establish a use case for this complexome resource by uncovering an unexpected exonucleolytic stabilization of sRNAs in the competence regulon, a pathway essential for DNA uptake and important for pneumococcal virulence (Lin *et al*, 2016; Muschiol *et al*, 2019; Salvadori *et al*, 2019).

## Results

### Grad‐seq captures major RNA–protein complexes of the pneumococcus

**Figure 1:**
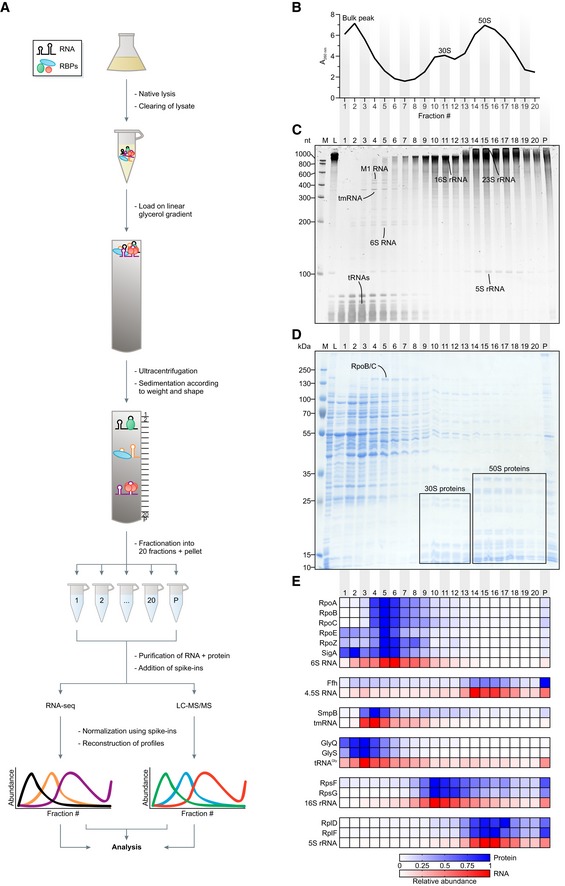
Grad‐seq reveals the RNA/protein complexome of *Streptococcus pneumoniae* AGrad‐seq workflow.BA_260 nm_ profile of the gradient with highlighted low‐molecular‐weight complexes (bulk peak) and ribosomal subunits (30S, 50S). Particles larger than the 50S subunit were pelleted.CEthidium bromide‐stained RNA gel. Bands corresponding to abundant housekeeping RNAs are indicated.DCoomassie‐stained SDS–PAGE. Bands corresponding to abundant housekeeping proteins are indicated.EHeat map of digital in‐gradient distributions of known RNA–protein complexes derived from RNA‐seq and LC‐MS/MS data. The profiles are normalized to the range from 0 to 1. RBP, RNA‐binding protein. LC‐MS/MS, liquid chromatography‐tandem mass spectrometry. M, size marker. L, lysate (input control). P, pellet fraction. Source data are available online for this figure.

To draft a digital map of soluble cellular complexes formed by pneumococcal RNA and proteins, a lysate of the model strain TIGR4 grown to mid‐exponential phase was fractionated on a linear glycerol gradient. Each of 20 fractions was then analyzed by RNA‐seq and mass spectrometry (Fig 1A). Quality control by A_260 nm_ measurements showed the expected sedimentation profile (Smirnov *et al*, 2016): one bulk peak in the low‐molecular‐weight (LMW) fractions and two additional peaks for the 30S and 50S ribosomal subunits (Fig 1B). Importantly, these three peaks corresponded well with ribosome‐free tRNAs, 16S rRNA, and 5S/23S rRNAs, respectively, visible in a stained RNA gel (Fig 1C). Additional abundant transcripts known to form stable complexes with proteins, e.g., 6S RNA, M1 RNA of RNase P, and tmRNA, were readily visible (Fig 1C), thus confirming the quality of the gradient. Complementary SDS gel analysis offered a coarse‐grained view of the in‐gradient distribution of some of the corresponding proteins of, e.g., RNAP [target of 6S RNA (Wassarman & Storz, 2000)] and the 30S and 50S ribosomal subunits (Fig 1D).

**Figure EV1:**
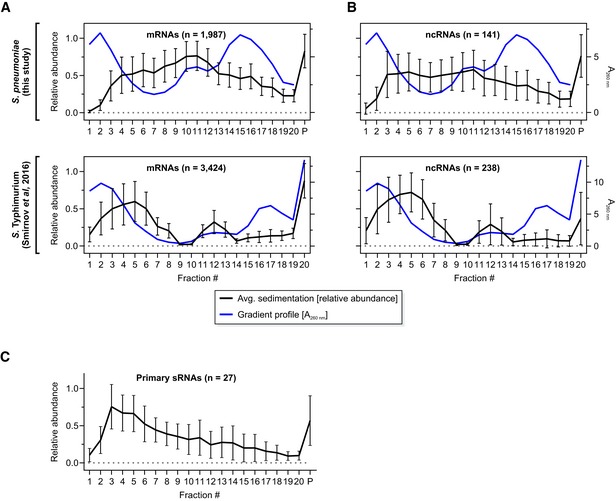
Average Grad‐seq transcript profiles A, BAverage mRNA (A) and ncRNA (B) profiles of *Streptococcus pneumoniae* (upper panels, this study) and, for comparison, *S*. Typhimurium [lower panels, data from Smirnov *et al* (2016)]. As a reference, UV profiles of the gradients were added in blue [Fig 1B and Smirnov *et al* (2016)].CAverage profile of high confidence, independently transcribed sRNAs.Data information: Error bars show SD from the mean.

RNA‐seq of the 20 fractions detected 2,240 transcripts, accounting for ~ 88% of the *S. pneumoniae* transcriptome (Dataset EV1). As seen previously with Gram‐negative *Salmonella* (Smirnov *et al*, 2016), mRNAs populated the whole gradient but peaked in the 30S and pellet (containing 70S ribosomes) fractions (Fig EV1A). Notable differences from *Salmonella* Grad‐seq are a slight shift of mRNAs toward the soluble fractions (Fig EV1A), indicative of partial ribosome dissociation, and a broader average distribution of ncRNAs as compared to the mRNAs (Fig EV1B). For the ncRNAs, this broader distribution may be due to the inclusion of various different classes such as riboswitches and sRNAs in the annotation used for the study here. Importantly, the sRNAs that are transcribed from independent genes (i.e., do not result from processing of longer transcripts) tend to be found in small complexes, as suggested by their accumulation in low‐molecular‐weight fractions (Fig EV1C).

**Figure EV2:**
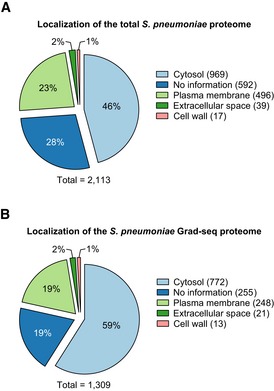
Localization of detected proteins A, BPredicted localization of the total (A) and the Grad‐seq (B) proteome of *Streptococcus pneumoniae*. Note that the numbers for (B) do not fit with Dataset EV2 because some proteins have more than one localization assigned. Predicted localizations were downloaded from BioCyc (Karp *et al*, 2019).

Complementary mass spectrometry analysis of the gradient detected 1,301 proteins (Dataset EV2), most of which were cytosolic and in sum represented ~ 62% of the annotated proteome (Fig EV2A and B). Of known stable RNPs, we observed excellent correlations in the digital RNA and protein data for 30S and 50S proteins with their respective rRNAs, as well as for the 6S RNA‐RNAP complex (Wassarman, 2018), the signal recognition particle (SRP; complex of Ffh protein and 4.5S RNA) (Akopian *et al*, 2013), and the tmRNA‐based ribosome rescue system (Keiler, 2015; (Fig 1E). Interestingly, different from *Salmonella*, where the SRP peaks in fraction 3 (Smirnov *et al*, 2016), the pneumococcal SRP sediments in the 50S region, suggesting that its quaternary configuration is more stable in lysed pneumococcal cells. Lastly, as observed previously (Smirnov *et al*, 2016), reads of major tRNAs such as those decoding glycine coincided with peptides of their corresponding tRNA synthetases (Fig 1E).

### Grad‐seq assists in the characterization of protein functions

Our focus on RNA–protein complexes notwithstanding, the digital in‐gradient distributions obtained by Grad‐seq proteomics should also aid the prediction of other macromolecular assemblies of proteins. For example, the nearly congruent profiles of the RpoA, RpoB, and RpoC proteins readily predict that they form a large complex, i.e., RNAP (Fig 1E).

**Figure 2:**
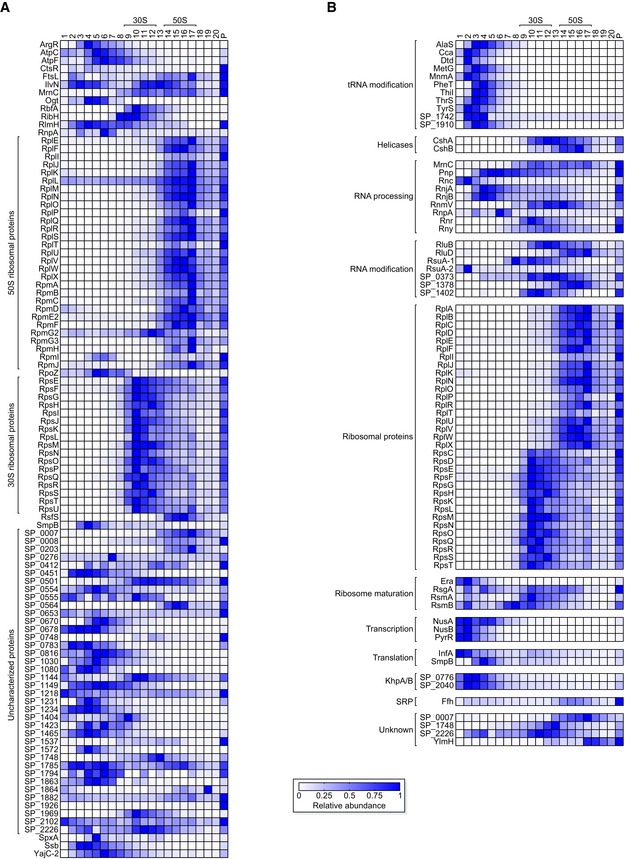
Grad‐seq predicts complexes AProfiles of 102 proteins < 20 kDa that show fast sedimentation, indicating involvement in complexes.BHeat map of migration profiles of 77 proteins with the UniProt keyword “RNA‐binding”. For clarity, they were assigned to functionally related classes of RBPs. Note that SP_2040 was added due to its interaction with SP_0776 in the KhpA/B heterodimer (Zheng *et al*, 2017; Winther *et al*, 2019), even though it does not have the keyword on UniProt.

To test this assumption, we focused our analysis on proteins < 20 kDa. Considering a moderately elongated shape, these proteins should sediment at < 3S (fractions ~ 1–3) unless in a complex (Erickson, 2009). Of 284 such proteins in total in our data, 102 showed top peaks in fraction 4 or higher (Dataset EV3), indicating complex formation (Fig 2A). This latter set contained many expected proteins, such as 46 known ribosomal proteins, RpoZ, SmpB, and RnpA. Further, the peaks of RbfA (ribosome‐binding factor A) and RsfS (ribosomal silencing factor) coincided with the 30S and 50S peaks, respectively, which agrees with their activities on disassembled subunits only (Shajani *et al*, 2011; Häuser *et al*, 2012).

Grad‐seq data also add confidence to domain‐based functional assignment of uncharacterized proteins. For example, SP_1969 is annotated as a member of the InterPro family IPR004398 (Mitchell *et al*, 2019) that also contains the 16S rRNA methyltransferase RsmD of *E. coli* (Lesnyak *et al*, 2007; Sergeeva *et al*, 2012; Appendix Fig S1). Grad‐seq showed SP_1969 peaking with the *S. pneumoniae* 30S proteins, suggesting that it has an RsmD‐like function in 30S subunit maturation. Another example is SP_0007, which, like its putative *E. coli* homolog Hsp15, contains an RNA‐binding S4 domain. Interestingly, both SP_0007 and its Gram‐positive homologs lack the disordered ~ 40 aa C‐terminus of *E. coli* Hsp15 (Appendix Fig S2) which increases the affinity of the latter to translationally inactive 50S subunits (Jiang *et al*, 2009). Nonetheless, *S. pneumoniae* SP_0007 peaked with the 50S proteins, supporting the prediction of a conserved role in 50S recycling. On the contrary, S4 domain protein SP_2226, whose *Bacillus subtilis* homolog YaaA was proposed to be involved in 50S assembly (Suzuki *et al*, 2014), co‐migrated primarily with the 30S proteins, therefore suggesting that it is involved in a different part of ribosomal biology than originally predicted. C‐termini of different lengths in *Streptococcus*,* Bacillus,* and *Staphylococcus* species (Appendix Fig S3) may alter the interactomes of these homologous proteins. Taken together, these examples illustrate how digital Grad‐seq data can, on the protein part, support functional predictions of uncharacterized pneumococcal proteins.

**Figure EV3:**
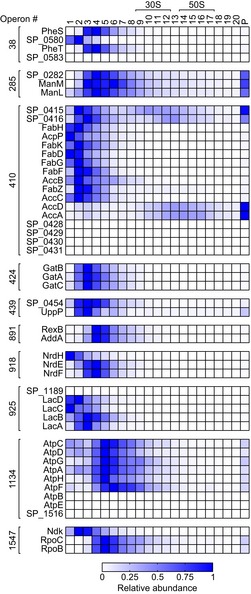
Grad‐seq profiles of proteins deriving from operons Exemplary operons [numbers according to Warrier *et al* (2018)] that provide evidence for predicted complexes.

Next, we interrogated the Grad‐seq proteomics data for co‐migrating proteins from 388 predicted operons of *S. pneumoniae* TIGR4 (Warrier *et al*, 2018; Dataset EV4), which often encode for proteins involved in the same complex (Wells *et al*, 2016). For the first time in the pneumococcus, this analysis proves higher‐order macromolecular assemblies such as glutamyl‐tRNA^Gln^ amidotransferase (GatBAC), ribonucleoside‐diphosphate reductase (NrdEF), galactose‐6‐phosphate isomerase (LacBA), and an intact 9‐subunit cytosolic ATP synthase *F*_1_ complex (AtpCDGAH) (Fig EV3). Surprisingly, although these proteins had been gradient‐fractionated from a cleared lysate, we observed co‐migrating proteins with predicted membrane localization. For example, the membrane‐associated, virulence‐related protein UppP (a.k.a. BacA) (Chalker *et al*, 2000) co‐migrated with SP_0454, a protein of unknown function with several trans‐membrane domains that are encoded in the same operon. While we cannot assess whether these putative membrane proteins remain properly folded, this illustrates the potential for predicting *in vivo* complexes beyond those formed by cytosolic proteins.

UniProt lists 78 *S. pneumoniae* TIGR4 proteins with the keyword “RNA‐binding”, 77 of which were detected in our gradient (Fig 2B). While tRNA‐modifying enzymes expectedly migrated in early fractions, others such as RNases showed no common profile. The RNA‐binding heterodimer KhpA/B, known as SP_0776 and SP_2040 in the strain used here, was recently reported to bind a diverse set of cellular transcripts, including tRNAs and sRNAs (Zheng *et al*, 2017). Here, we detected a stable KhpA/B complex with a peak in fractions 2–3, which coincided with tRNAs and primary sRNAs (i.e., those transcribed from independent genes) (Fig EV1C). Taken together, these profiles provide the first global information about which RBPs in Gram‐positive bacteria may exist within macromolecular assemblies.

### Identification of biochemically distinct classes of ncRNAs

**Figure 3:**
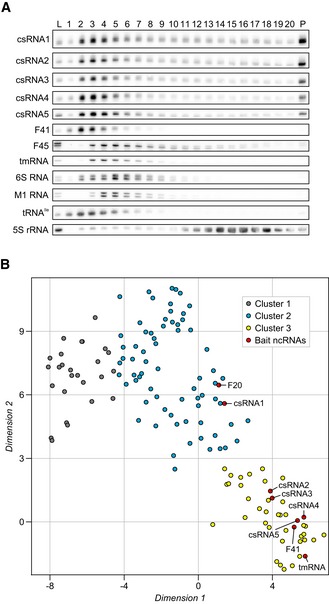
Grad‐seq identifies clusters of biochemically distinct ncRNAs ANorthern blots of ncRNAs. The different classes of ncRNAs show unique profiles on the gradient. Of note, the functionally related csRNAs co‐migrate. L, lysate (input control). P, pellet fraction.Bt‐SNE analysis of all 141 ncRNAs detected by Grad‐seq. Cluster 3 was of particular interest since it contained mostly ncRNAs present in low‐molecular‐weight fractions (Fig EV4F). ncRNAs selected for downstream pull‐down experiments are highlighted in red. Source data are available online for this figure.

**Figure EV4:**
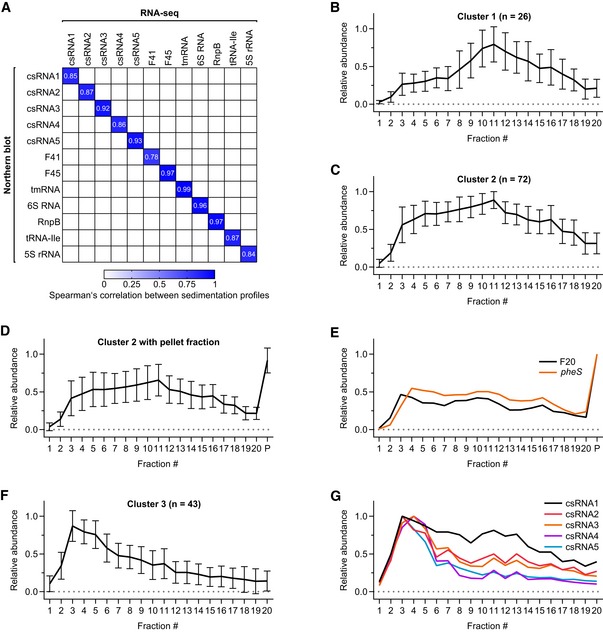
Grad‐seq profiles of ncRNAs AHeat map of Spearman's correlation between northern blot profiles (Fig 3A) and RNA‐seq profiles for selected ncRNAs. Two‐tailed *P*‐values for all correlations are 2.77 × 10^−17^ < *P < *2.60 × 10^−5^.B–FAverage ncRNA profiles for cluster 1 (B), cluster 2 (C), cluster 2 including the pellet fraction (D), and cluster 3 (F) show distinct sedimentation, separating them from each other (Fig 3B). Cluster 2 includes the riboswitch RNA F20, which has similar sedimentation to its downstream CDS *pheS* (E).GRNA‐seq profiles of the five csRNAs. Note that csRNA1 has a clear peak in fraction 3, albeit not as pronounced as the other csRNAs.Data information: Error bars show SD from the mean.

ncRNAs are particularly promising with respect to RBP discovery because the well‐studied ones usually require helper proteins for cellular function or stability (Gorski *et al*, 2017; Holmqvist & Vogel, 2018). Importantly, we observed migration patterns of many pneumococcal ncRNAs that were indicative of RNPs different from the major RNPs described above. Northern blot analysis of several such ncRNAs (Fig 3A) showed strong correlation of their in‐gradient migration with the digital RNA‐seq data (Fig EV4A). For example, the five competence‐regulating cia‐dependent small RNAs (csRNAs) (Halfmann *et al*, 2007) all showed profiles very different from the ubiquitous tmRNA, 6S RNA, and M1 RNA (Fig 3A).

A general assumption in Grad‐seq is that transcripts with similar in‐gradient distributions share interacting RBPs (Smirnov *et al*, 2017a). To define biochemically similar ncRNAs as potential baits for subsequent RBP recovery, we performed a t‐SNE analysis on the RNA‐seq data of gradient fractions 1–20 (Fig 3B, Appendix Fig S4). This yielded three distinct clusters of which cluster 1 contained 26 ncRNAs that generally showed low abundance in the LMW fractions and peaks around the ribosomal subunits (Fig EV4B). As expected, this cluster contained the 4.5S RNA component of the ribosome‐associated SRP, but also several ncRNAs with previously demonstrated roles in virulence, e.g., F48, F62, F65, and R8 (Mann *et al*, 2012). Of note, many of these latter ncRNAs originate from repetitive elements and show poor sequence conservation in closely related species, hampering the evaluation of potential overlooked ORFs that would explain their sedimentation in the ribosomal fraction. Cluster 2 contained 72 ncRNAs that generally exhibited broad distribution in the gradient (Fig EV4C), and most (~ 69%) of these were also much more abundant in the pellet than in the gradient fractions (Fig EV4D). A main peak in the pellet indicates 70S ribosome association, which in the exemplary case of the F20 ncRNA (a T‐box riboswitch) may be driven by the parental *pheS* mRNA (Fig EV4E).

Cluster 3 containing 43 ncRNAs with a tendency to peak before the 30S subunit (Fig EV4F) was the most interesting. This cluster contained not only ncRNAs of well‐characterized stable RBPs but also 21 primary sRNAs (Fig EV1C), including four of the five csRNAs. The csRNAs are post‐transcriptional repressors of *comC* mRNA encoding the precursor protein of the pneumococcal competence‐stimulating peptide (CSP). Without synthesis of CSP, competence and therefore DNA uptake from the environment cannot be induced (Schnorpfeil *et al*, 2013; Laux *et al*, 2015). Molecular details of csRNA‐mediated control and the involvement of RBPs are yet to be determined. To us, their co‐migration in the gradient indicated that they shared associated protein(s).

### Cbf1 is an sRNA‐interacting protein

To identify csRNA‐interacting proteins, we sought to co‐purify them from lysates using *in vitro*‐transcribed MS2‐tagged csRNAs as baits, i.e., using the same strategy that recently enabled us to identify a new global RBP (ProQ) in enteric bacteria (Smirnov *et al*, 2016). Several additional ncRNAs were included for comparison or controls: the functionally related csRNA1, which ended up in cluster 2, probably because its peak in fractions 3–4 was weaker than for the other csRNAs (Fig EV4G); riboswitch‐derived F20 from cluster 2; F41 sRNA from cluster 3, which also clearly peaks in the LMW fractions; and tmRNA, which is known to bind the SmpB protein (Fig 3B). For unclear reasons, attempts to use the MS2 aptamer, which successfully captured RBPs and RNA–RNA interactions from *Salmonella*,* E. coli,* and *Staphylococcus aureus* lysates (Said *et al*, 2009; Corcoran *et al*, 2012; Lalaouna *et al*, 2015, 2019; Smirnov *et al*, 2016; Tomasini *et al*, 2017), failed. While we did recover the MS2‐tagged RNAs after incubation with lysates, no protein co‐purified at all.

**Figure 4:**
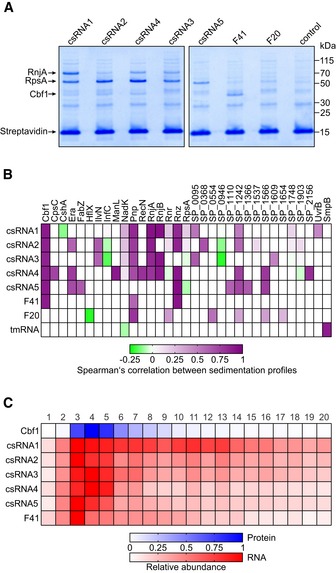
Identification of Cbf1 as a ncRNA‐binding protein ARepresentative gel images of pull‐downs performed with ncRNAs as baits (compare to Fig 3B). Cbf1 was identified as a specific binder of all csRNAs and F41, but not of riboswitch RNA F20.BHeat map showing Spearman's correlation coefficients between the sedimentation profiles of bait ncRNAs and proteins enriched in the pull‐downs. Ribosomal proteins except for S1 (RpsA) and proteins only co‐purified with tmRNA (except for SmpB) are omitted for clarity. Cbf1 is a recurring binding partner of the bait ncRNAs that also shows highly correlating sedimentation profiles in the gradient.CHeat map showing gradient profiles of Cbf1, csRNAs, and F41. Cbf1 co‐sediments with these sRNAs. Source data are available online for this figure.

Therefore, we adopted a protocol developed for eukaryotic RBPs in which the bait RNA is *in vitro* transcribed with a 5′ located, 14 nt capture tag (Treiber *et al*, 2017). Using this alternative procedure, we successfully enriched proteins for each of the tagged pneumococcal ncRNAs (Fig 4A). LC‐MS/MS‐based identification of these proteins (Appendix Fig S5A–H) followed by comparison of their in‐gradient distributions with those of the respective ncRNAs used as baits (Fig 4B) indicated strong enrichment of several degradosome components. Of these, RNases J1 and J2, as well as PNPase to some extent, correlated well with the respective ncRNA baits. Another recurring protein was the essential RNase Z, as pulled down by F41 and all csRNAs except csRNA3. However, the highest correlation of all enriched proteins was observed for Cbf1: This protein stably associated with all csRNAs and F41 (Fig 4C) but did not elute with tmRNA or riboswitch RNA F20 (Appendix Fig S5G and H).

**Figure 5:**
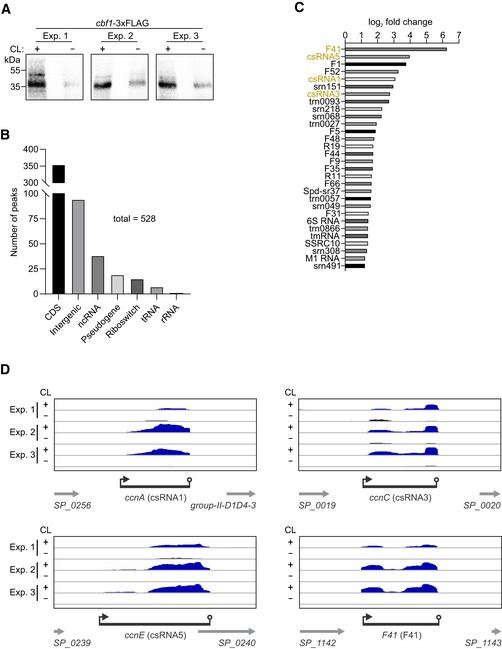
CLIP‐seq validates ncRNA binding of Cbf1 *in vivo* AAutoradiographs of radioactively labeled RNA fragments covalently bound to Cbf1 after *in vivo* UV crosslinking, immunoprecipitation, gel electrophoresis, and membrane transfer. Three independent experiments are shown that were subjected to sequencing. CL, UV crosslinking. Exp., experiment.BOrigins of the peaks found in the Cbf1 CLIP‐seq dataset.CEnrichment of the 30 ncRNAs found in the CLIP‐seq dataset. F41, csRNA1, csRNA3, and csRNA5 (highlighted in orange) are strongly enriched and were previously used to pull‐down Cbf1 (Fig 4A and B). If more than one peak was identified for a single ncRNA in the dataset, only the highest fold change is shown.DCLIP‐seq coverage plots of csRNA1 (*ccnA*), csRNA3 (*ccnC*), csRNA5 (*ccnE*), and F41 (*F41*) showing crosslinking‐dependent enrichment of peaks at the 3′ ends of the transcripts. Source data are available online for this figure.

To validate the proposed Cbf1–RNA interaction, we also performed the reverse experiment: UV CLIP‐seq using a *cbf1‐*3xFLAG strain. UV crosslinking resulted in a ~ 5–10‐fold overall enrichment of RNA compared to the non‐crosslinked control (Fig 5A). Deep sequencing of the bound RNA fragments yielded 528 peaks that passed our stringent cutoffs, i.e., a log_2_ fold change > 1 and an adjusted *P*‐value < 0.01 (Fig 5B and Dataset EV5). 354 (~ 67%) of these peaks derived from CDSs, whereas 94 (~ 18%) peaks could be attributed to intergenic sequences, which originated from unannotated antisense transcripts, intergenic transcripts, and UTRs. 38 (~ 7%) peaks mapped to annotated ncRNAs, 30 of which were unique (Fig 5C). Importantly, F41 was the most enriched ncRNA in the dataset, matching the strong enrichment of Cbf1 in the F41 RNA pull‐down (Fig 4A). As expected, we further found csRNA1, csRNA3, and csRNA5 to be among the most enriched ncRNAs (Fig 5C). csRNA2 and csRNA4 were not enriched, which could be due to an unfavorable constellation of crosslinking‐prone nucleotides in these transcripts. Other detected, mildly enriched ncRNAs included 6S RNA, tmRNA, and M1 RNA, all of which are abundant RNAs that tend to crosslink to RBPs, as already observed previously (Holmqvist *et al*, 2018). The identified peaks mostly mapped to the 3′ ends of the sRNAs, whereas there was little‐to‐no coverage toward 5′ ends (Fig 5D). The observed binding of Cbf1 at the 3′ ends of its targets supports its predicted role as a 3′→5′ exonuclease (see below). Taken together, Cbf1 was identified as a new ncRNA‐associated protein in the pneumococcus and was therefore selected for deeper investigation.

### Cbf1 processes and stabilizes csRNAs

Cbf1 (*cmp*‐binding factor 1) was first described as a host factor for replication of plasmid pT181 in *S. aureus* (Zhang *et al*, 1997). Subsequently, both *S. aureus* Cbf1 and its *B. subtilis* homolog YhaM were shown to be manganese‐dependent 3′→5′ exonucleases that are present in a wide range of Gram positives (Oussenko *et al*, 2002, 2005; Fang *et al*, 2009; Redko & Condon, 2010). Most recently, an RNA‐seq study in *Streptococcus pyogenes* concluded that YhaM acted to trim transcripts by a few nucleotides until it reaches a stem‐loop structure such as a Rho‐independent terminator (Lécrivain *et al*, 2018). However, specific physiological functions of Cbf1/YhaM proteins remain unknown.

**Figure 6:**
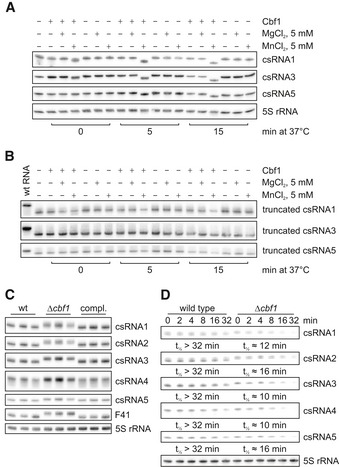
Cbf1 acts as an sRNA‐stabilizing 3′→5′ exonuclease A
*In vitro* RNase assay. *In vitro*‐transcribed RNAs were incubated in the presence or absence of Cbf1, MgCl_2_, and MnCl_2_. Reactions were stopped at the indicated time points and the RNA run on denaturing 10% PAGE followed by northern blotting. All csRNAs, but not 5S rRNA, are cleaved in the presence of Cbf1 and MnCl_2_.B
*In vitro* RNase assay with truncated RNAs. The setup of the experiment was the same as in A, the difference being that the *in vitro*‐transcribed RNAs were missing the single‐stranded uridine overhang 3′ of the Rho‐independent terminator. Wild‐type (wt) RNA used in A was loaded as reference. No cleavage can be observed in any of the conditions.CNorthern blots of denaturing 10% PAGE of total RNA extracted from wild type (wt), Δ*cbf1* and the complementation (compl.) strain. Upon deletion of *cbf1*, an upshift of the tested ncRNAs, but not of 5S rRNA is visible.D
*In vivo* RNA stability assays. After blocking transcription with rifampicin, RNA was collected at the indicated time points and visualized using northern blotting of 6% denaturing PAGE. Interestingly, knockout of *cbf1* increases stability of the tested RNAs. 5S rRNA was used as loading control. RNA half‐lives were calculated based on at least two independent experiments (Fig EV5B–F). Source data are available online for this figure.

We validated the predicted 3′→5′ RNase activity by incubating recombinant *S. pneumoniae* Cbf1 protein with *in vitro*‐transcribed ncRNAs (Fig 6A). Matching the *in vivo* observations in *S. pyogenes* (Lécrivain *et al*, 2018), Cbf1 trimmed the RNA substrates by several nucleotides, as judged by their faster migration in a gel. No shortening was observed with 5S rRNA (Fig 6A), which is known to be processed by RNase M5 (Bechhofer & Deutscher, 2019).

Cbf1‐mediated trimming required the presence of Mn^2+^ ions, thus recapitulating the manganese dependence of *B. subtilis* YhaM (Oussenko *et al*, 2002; Fang *et al*, 2009). At the concentration used, the trimming occurred fast, as evident from a slight shift immediately after Cbf1 was added (time point 0 min). After 5 and 15 min of incubation, the RNAs migrated as single, shorter bands, suggesting complete digestion. Importantly, Cbf1 did not process 3′‐truncated RNAs lacking the uridine stretch of their Rho‐independent terminators, confirming these single‐stranded 3′ overhangs as its targets (Fig 6B). We note that truncated csRNA1 showed reduced RNA levels after incubation with Cbf1. Probing of the 5′ end of this RNA revealed a ~ 45 nt RNA species specific for manganese‐dependent cleavage by Cbf1, which is not visible when probing for the 3′ end (Appendix Fig S6A and B). Subsequent 3′ RACE determined the 3′ nucleotide of this short species to be at the C at position 45 of the truncated csRNA1, which lies directly upstream of a uridine stretch (Appendix Fig S6C–E). The predicted secondary structure of this 45 nt species contains a weak stem‐loop upstream of its 3′ end, with a short single‐stranded overhang (Appendix Fig S6F). We speculate that removal of the uridine stretch from the full‐length RNA probably leaves a less protective 3′ end structure, which then allows Cbf1 to digest more of the RNA.

**Figure EV5:**
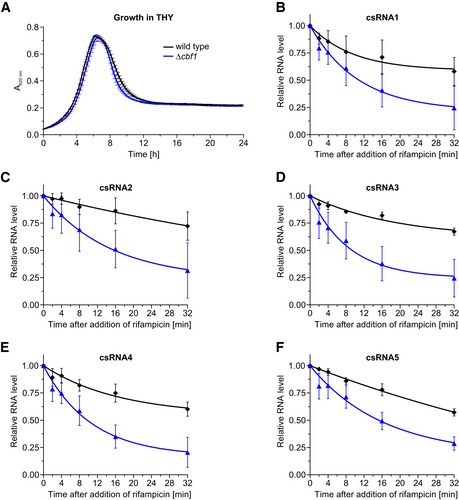
Growth and csRNA stability in the absence of *cbf1* AGrowth curves in rich medium. Deletion of *cbf1* has no influence on growth when compared to the wild type. The average cell density is based on three independent biological replicates.B–FDetermination of the stability of csRNAs based on rifampicin assays shown in Fig 6D. In the absence of *cbf1*, csRNAs become less stable when compared to the wild type. The relative RNA levels were calculated based on at least two independent experiments.Data information: Error bars show SD from the mean.

Next, we studied Cbf1‐dependent processing *in vivo*, probing total RNA from wild‐type and Δ*cbf1* strains on northern blots (Fig 6C). As expected, both the csRNAs and F41 showed slower migration in the knockout mutant, with their lengths being restored by chromosomal complementation of *cbf1*. Note that, in rich medium, the *cbf1* deletion does not affect bacterial growth (Fig EV5A). To address the consequence of trimming by Cbf1, we monitored the decay of target transcripts after shutting off transcription with rifampicin (Fig 6D). Intriguingly, the half‐lives of all csRNAs were diminished from > 32 min in the wild‐type strain to approximately 10–16 min in the *cbf1* knockout, suggesting a protective function of Cbf1 (Fig EV5B–F). Therefore, we interpret the Cbf1–RNA complexes detected by Grad‐seq as stable intermediates that persist after 3′ trimming and stabilize the associated sRNAs by protecting them from further degradation.

### Cbf1 is a negative regulator of competence

**Figure 7:**
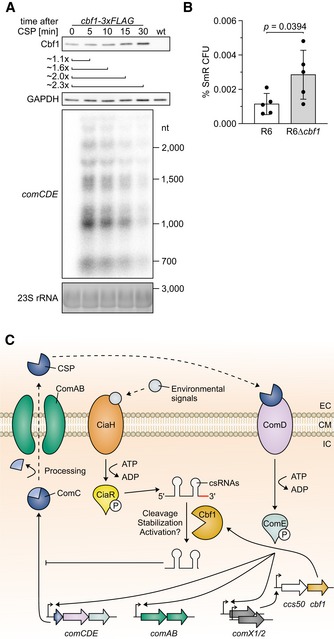
Cbf1 negatively regulates competence AWestern blot of Cbf1‐3xFLAG levels after CSP stimulation. After 15 min, ˜ 2‐fold higher Cbf1‐3xFLAG levels could be detected, confirming *cbf1* as a late‐competence gene as predicted by others (Peterson *et al*, 2004; Slager *et al*, 2019). CSP‐specific fold changes in Cbf1‐3xFLAG levels were calculated based on three independent experiments (Appendix Fig S7A). As control for the CSP induction, total RNA was extracted, run on an agarose gel, blotted, and probed for *comCDE*. A GAPDH‐specific antiserum and ethidium bromide staining were used as loading controls for the Western and northern blots, respectively.BPercentage of transformants using R6 and R6Δ*cbf1* in a spontaneous transformation assay. Bars represent the mean ± standard deviation percentage SmR CFU from 5 independent experiments. *P*‐value was calculated using an unpaired, two‐tailed *t*‐test.CModel of Cbf1‐dependent regulation of competence. The pneumococcal competence regulon is a quorum sensing system induced by activation of ComDE via CSP. Phosphorylated ComE induces transcription of the early competence gene loci *comAB*,* comCDE*,* comX1,* and *comX2*, leading to a positive feedback loop by production of ComC, which is processed into CSP and exported by ComAB. The paralogous alternative sigma factors ComX1 and ComX2 induce transcription of late‐competence gene loci, one of which is *ccs50/cbf1* (Slager *et al*, 2019). Cbf1 is able to cleave, stabilize, and possibly activate csRNAs, which are induced by CiaRH, which in turn is activated by a variety of factors (Gómez‐Mejia *et al*, 2018). csRNAs are able to post‐transcriptionally downregulate *comC*. EC, extracellular. CM, cell membrane. IC, intracellular. CSP, competence‐stimulating peptide. Source data are available online for this figure.

The observed stabilization of csRNAs links Cbf1 to the regulation of competence. Intriguingly, others have observed in a global transposon screen that disruption of *cbf1* reduces fitness in transformation conditions, compromises the colonization of the murine lung, and terminates the colonization of the murine nasopharynx (van Opijnen & Camilli, 2012). Moreover, a recent gene expression catalogue for *S. pneumoniae* strain D39 grown in many different infection‐relevant conditions predicts a specific upregulation of *cbf1* when competence is stimulated by CSP (Aprianto *et al*, 2018). To test this prediction, we measured *cbf1* expression after stimulating *S. pneumoniae* strain TIGR4 with CSP. We observed higher levels of both the Cbf1 protein (Fig 7A and Appendix Fig S7A; dependence on CSP stimulation shown in Appendix Fig S7B) and the *cbf1* mRNA (Appendix Fig S7C) under this condition, thus validating *cbf1* as a member of the competence regulon.

The established function of the csRNAs is that they repress the *comC* mRNA and thereby the synthesis of CSP, thus counteracting competence activation. If the Cbf1‐mediated csRNA stabilization were of physiological relevance, the absence of this protein should render *S. pneumoniae* more competent. We were unable to obtain transformants in the absence of exogenously added CSP with *S. pneumoniae* strain TIGR4, in contrast to strain R6, which is known to spontaneously develop competence when grown at pH 7.9–8.0 (Moscoso & Claverys, 2004). Hence, we constructed a knockout mutant strain in R6, R6Δ*cbf1*, and tested the predicted role of *cbf1* using a spontaneous competence assay. We indeed observed ~ 2.5‐fold higher transformation in the Δ*cbf1* strain as compared to the wild‐type strain R6 (Fig 7B) with all replicates showing the same positive trend (Appendix Fig S7D). This suggests a model, whereby Cbf1‐mediated csRNA stabilization contributes to a negative feedback loop that controls competence after CSP stimulation (Fig 7C).

## Discussion

Our Grad‐seq analysis of *S. pneumoniae* provides the first global census of major stable complexes formed by RNAs and proteins in a Gram‐positive bacterium. The results not only provide crucial knowledge for this important human pathogen, but should also be a valuable resource for other Gram‐positive species including the model bacteria *B. subtilis, Listeria monocytogenes*,* S. aureus,* and *S. pyogenes*. Establishing a use case for the Grad‐seq data, we identify a novel physiological function of the conserved exonuclease Cbf1 in trimming and thus stabilizing the csRNAs that are central regulators of competence. As discussed below, the data now permit conclusions as to what may be conserved major RNA–protein complexes and global RBPs outside the well‐characterized enterobacterial clade.

Although the pneumococcus expresses a great number of sRNAs with evidence for important physiological roles (Mann *et al*, 2012; Laux *et al*, 2015; Wilton *et al*, 2015), homologs of CsrA, Hfq, or ProQ are not present in this organism (Tettelin *et al*, 2001). A preliminary prediction from our cluster analysis and subsequent protein co‐purification experiments is that *S. pneumoniae* perhaps does not possess a general RBP analogous to Hfq or ProQ, the two RBPs that together associate with > 80% of the sRNAs in *E. coli* and *Salmonella* (Holmqvist & Vogel, 2018). Therefore, the csRNAs may be the first example of redundant sRNAs that regulate complex behavior without an RNA chaperone, i.e., different from the well‐studied quorum sensing‐related Qrr sRNAs in *Vibrio* spp. that crucially depend on Hfq (Feng *et al*, 2015). More generally, the *S. pneumoniae* sRNAs may rely upon more specialized RBPs such as the KhpA/B complex (Zheng *et al*, 2017) instead of a common RNA chaperone. Grad‐seq readily captures the KhpA/B complex, detecting nearly congruent peaks of KhpA and KhpB in gradient fractions 2–3 (Fig 2B). These narrow peaks in the LMW fractions differ from the profiles of Hfq and ProQ in *Salmonella* (Smirnov *et al*, 2016), suggesting that the KhpA/B complex stably associates with fewer cellular transcripts than those global RBPs.

Of more widely conserved RNA–protein constellations, 6S RNA and RNAP show overlapping peaks in the gradient (Fig 1E), supporting previous reports for such a complex in *B. subtilis* (Trotochaud & Wassarman, 2005; Cavanagh *et al*, 2012; Burenina *et al*, 2014). However, in light of the available copy numbers of the involved RNA and proteins in *S. pneumoniae*, their Grad‐seq‐based capture suggests that the 6S RNA‐RNAP complex may be the major cellular form of RNAP when it is not transcribing DNA. We propose that the 6S RNA–RNAP complex, two decades after its discovery in *E. coli* (Wassarman & Storz, 2000), should be considered on par with RNase P, SRP, tmRNA‐SmpB, and the ribosome when it comes to ubiquitous RNPs in bacteria.

We have discovered stable *in vivo* complexes of the 3′→5′ exonuclease Cbf1 (Figs 4 and 5) and shown that, contrary to expectation, the associated csRNAs are stabilized by Cbf1 (Figs 6D and EV5B–F). This stabilization, however, makes sense as one considers the documented physiological function of the csRNAs that—after their upregulation by CiaRH—act to post‐transcriptionally repress ComC synthesis (Laux *et al*, 2015; Gómez‐Mejia *et al*, 2018). We propose a model in which Cbf1 binds and trims the csRNAs by a few nucleotides and, through its continued association, shields them from other decay enzymes. If so, loss of Cbf1 function should decrease the post‐transcriptional inhibition of the competence regulon by the csRNAs. In support of this model, we find that a Δ*cbf1* strain is more competent (Fig 7B). Importantly, *cbf1* was proposed to constitute a ComX‐activated late‐competence gene (Peterson *et al*, 2004; Slager *et al*, 2019), which we validated here on both the RNA and protein levels (Fig 7A, Appendix Fig S7A–C). Alternatively, Cbf1‐dependent trimming could increase the activity of csRNAs, similar to microRNA, piRNA, and siRNA trimming in eukaryotes (Han *et al*, 2011; Liu *et al*, 2011; Marasovic *et al*, 2013; Tang *et al*, 2016). Overall, this finding is relevant for *in vivo* survival: Disruption of the *cbf1* gene renders the pneumococcus unable to infect the murine nasopharynx and strongly attenuates lung colonization (van Opijnen & Camilli, 2012).

Arguably, our model does not exclude possible csRNA‐independent functions of Cbf1 in competence regulation, which remain to be tested by combined inactivation of *cbf1* and multiple csRNAs. These regulations may indeed be more complex: Others have reported that competence is reduced (20% down) when *cbf1* is deleted in a different *S. pneumoniae* strain and exogenous CSP is added (Peterson *et al*, 2004). Further, in the related species *S. pyogenes*, Cbf1 (YhaM) acts on a large number of transcripts (Lécrivain *et al*, 2018), which we also observed here (Fig 5B). These observations notwithstanding, the evidence for a direct function of Cbf1 on the csRNAs is compelling. We have reconstituted *in vitro* the protective target trimming by Cbf1 and confirmed that, as in the original observations with *B. subtilis* YhaM (Oussenko *et al*, 2002; Fang *et al*, 2009), this reaction is Mn^2+^‐dependent (Fig 6A). Intriguingly, Cbf1 is the first 3′→5′ exonuclease in Gram‐positive bacteria with a protective effect on sRNAs, and the second when PNPase‐mediated stabilization of some Hfq‐dependent sRNAs in *E. coli* is counted (Cameron *et al*, 2018, 2019). This protection is also reminiscent of certain eukaryotic ncRNAs, which can be trimmed at their 3′ ends by PARN and TOE1, leading to enhanced stability (Berndt *et al*, 2012; Tseng *et al*, 2015; Son *et al*, 2018).

While our previous Grad‐seq analysis in *Salmonella* focused on complex formation of cellular RNA species, the depth of the present proteomics analysis for the *S. pneumoniae* gradient bears great potential for prediction and validation of protein–protein interactions as well. Importantly, Grad‐seq drafts a global landscape of the major protein complexes *in vivo* in a single experiment, without the massive epitope‐tagging of individual proteins in previous binary protein–protein interactome studies (Hu *et al*, 2009; Rajagopala *et al*, 2014). As such, our Grad‐seq proteomics data will help to add confidence to the available pneumococcal protein–protein interactions obtained in a yeast two‐hybrid screen (Wuchty *et al*, 2017). Illustrating potential, we find that the putative 16S rRNA methyltransferase homolog SP_1969, which might be involved in ribosome maturation and bacterial fitness (Lesnyak *et al*, 2007; Burakovsky *et al*, 2012; Sergeeva *et al*, 2012), co‐migrates with the 30S ribosome (Fig 2A). Analogously, the putative Hsp15 homolog SP_0007 co‐migrates with the 50S ribosome, supporting its possible role as a factor that recycles dead‐end 50S subunits (Korber *et al*, 2000; Jiang *et al*, 2009). In contrast, SP_2226 co‐migrated mostly with the 30S ribosome, even though its *B. subtilis* homolog YaaA was suggested to be a player in 50S assembly (Suzuki *et al*, 2014), indicating that SP_2226 could have alternative functions in the pneumococcus.

Taken together, our Grad‐seq study adds a new level of information to the increasing number of high‐throughput studies of the pneumococcus. It will provide an important link between predictions of protein–protein interactions (Wuchty *et al*, 2017) and the wealth of recent transcriptomics‐based studies (Acebo *et al*, 2012; Mann *et al*, 2012; Aprianto *et al*, 2018; Slager *et al*, 2018, 2019; Warrier *et al*, 2018; Sinha *et al*, 2019) in the quest to better understand the molecular underpinnings of pneumococcal physiology and virulence.

## Materials and Methods

### Bacteria and media

*Streptococcus pneumoniae* strains TIGR4 and R6 were streaked on tryptic soy agar plates containing 5% sheep blood (Oxoid) and grown overnight at 37°C and 5% CO_2_. Strains used are listed in Table EV1. The next day, pre‐cultures were grown in THY medium (to the exception of competence assays, see below) [Todd Hewitt broth (BD Bacto) supplemented with 0.5% yeast extract (Carl Roth)] to an OD_600 nm_ of 0.5 at 37°C without shaking. Subsequently, the pre‐culture was refreshed in THY to a starting OD_600 nm_ of 0.05 and grown at 37°C without shaking to a final OD_600 nm_ of 0.5.

### Glycerol gradient fractionation

400 ml of *S. pneumoniae* TIGR4 wild type was grown to an OD_600 nm_ of 0.5, cooled down in an ice‐water bath for 15 min, and then harvested by centrifugation for 20 min at 4°C and 4,000 *g*. The cells were washed three times in ice‐cold 1× TBS, resuspended in 500 μl ice‐cold 1× lysis buffer A [20 mM Tris–HCl, pH 7.5, 150 mM KCl, 1 mM MgCl_2_, 1 mM DTT, 1 mM PMSF, 0.2% Triton X 100, 20 U/ml DNase I (Thermo Fisher Scientific), 200 U/ml RNase inhibitor]. Mechanical lysis was performed using the FastPrep‐24 instrument (MP Biomedicals) in 2‐ml tubes with lysing matrix E (MP Biomedicals) at 6 m/s for 30 s. To remove insoluble debris and the beads, the lysate was cleared by centrifugation for 30 min at 4°C and 16,100 *g*. Of the cleared lysate, 10 μl was mixed with 1 ml TRIzol (Thermo Fisher Scientific) for the RNA input control and 20 μl was mixed with 20 μl 5× protein loading buffer for the protein input control.

400 μl of the cleared lysate was then layered on top of a linear 10–40% (w/v) glycerol gradient (in 1× lysis buffer A without DNase I or RNase inhibitor), which was formed in an open‐top polyallomer tube (Seton) using the Gradient Station model 153 (Biocomp). The gradient was centrifuged for 17 h at 4°C and 100,000 *g* (23,700 rpm) using a Beckman Coulter SW40Ti rotor, followed by manual fractionation into 20 590 μl fractions and measurement of the A_260 nm_ of each fraction. 90 μl of each fraction and 40 μl of the pellet were mixed with 30 μl of 5× protein loading buffer for protein analysis and stored at −20°C.

The remaining 500 μl of each fraction was used for RNA isolation by addition of 50 μl of 10% SDS (25 μl for the pellet) and 600 μl of acidic phenol/chloroform/isoamyl alcohol (P/C/I; 300 μl for the pellet). The fractions were then vortexed for 30 s and let rest at room temperature for 5 min before separating the phases by centrifugation for 15 min at 4°C and 16,100 *g*. The aqueous phases were collected, and 1 μl of GlycoBlue (Thermo Fisher Scientific) and 1.4 ml of ice‐cold ethanol/3 M NaOAc, pH 6.5 (30:1) were added and precipitated for at least 1 h at −20°C. The RNA was collected by centrifugation for 30 min at 4°C and 16,100 *g* and washed with 350 μl ice‐cold 70% ethanol, followed by centrifugation for 15 min at 4°C and 16,100 *g*. The lysate RNA sample stored in TRIzol was purified according to the manufacturer's protocol, except that the precipitation was performed using the mentioned ethanol mix. After drying of the RNA pellet, it was dissolved in 40 μl DEPC‐treated H_2_O and DNase‐digested by addition of 5 μl DNase I buffer with MgCl_2_ (Thermo Fisher Scientific), 0.5 μl RNase inhibitor, 4 μl DNase I (Thermo Fisher Scientific), and 0.5 μl DEPC‐treated H_2_O, followed by incubation for 45 min at 37°C. The DNase‐treated RNA was purified by the addition of 150 μl DEPC‐treated H_2_O and 200 μl acidic P/C/I as described above. The purified, DNase‐treated RNA was dissolved in 35 μl DEPC‐treated H_2_O and stored at −80°C.

### RNA gel electrophoresis and northern blotting

Equal volumes of the gradient RNA samples were separated by denaturing 6% PAGE in 1× TBE and 7 M urea and stained with ethidium bromide. For northern blotting, unstained gels were transferred onto Hybond+ membranes (GE Healthcare Life Sciences) and probed with RNA‐specific radioactively labeled DNA oligonucleotides.

### Protein gel electrophoresis and Western blotting

Equal volumes of the gradient protein samples were separated by 12% SDS–PAGE and stained with Coomassie. For Western blotting of Cbf1‐3xFLAG, unstained gels were transferred to PVDF membranes (GE Healthcare Life Sciences) and probed with an anti‐FLAG primary antibody (Sigma, cat# F1804, clone M2) and an anti‐mouse secondary antibody (Thermo Fisher Scientific, cat# 31430). As loading controls, the membranes were stripped and probed for glyceraldehyde‐3‐phosphate dehydrogenase (GAPDH) using a rabbit antiserum. Immunoreactive serum to *S. pneumoniae* GAPDH (GenBank AAK76079) was raised in rabbit against a synthetic peptide (DPIVSSDIVGMS) corresponding to amino acids 275–286. Synthetic peptide production, immunization, and validation by ELISA were done by Innovagen (Lund, Sweden), following a 41‐day immunization protocol including two booster injections. It was further used in previous studies (Codemo *et al*, 2018; Pathak *et al*, 2018). Detection of the GAPDH‐specific serum was performed using an anti‐rabbit secondary antibody (Thermo Fisher Scientific, cat# 31460).

### RNA‐seq

For RNA‐seq, 5 μl of the gradient samples was diluted in 45 μl DEPC‐treated H_2_O. 10 μl of the resulting 1:10 dilution was mixed with 10 μl of a 1:100 dilution of the ERCC spike‐in mix 2 (Thermo Fisher Scientific) and subjected to library preparation for next‐generation sequencing (Vertis Biotechnologie). Briefly, the RNA samples were fragmented using ultrasound (four pulses of 30 s at 4°C) followed by 3′ adapter ligation. Using the 3′ adapter as primer, first‐strand cDNA synthesis was performed using M‐MLV reverse transcriptase. After purification, the 5′ Illumina TruSeq sequencing adapter was ligated to the 3′ end of the antisense cDNA. The resulting cDNA was PCR‐amplified to about 10–20 ng/μl using a high‐fidelity DNA polymerase followed by purification using the Agencourt AMPure XP Kit (Beckman Coulter Genomics). The cDNA samples were pooled with ratios according to the RNA concentrations of the input samples, and a size range of 200–550 bp was eluted from a preparative agarose gel. This size‐selected cDNA pool was finally subjected to sequencing on an Illumina NextSeq 500 system using 75 nt single‐end read length.

### RNA‐seq data analysis

Read trimming and clipping were done with cutadapt (Martin, 2011). Further analysis steps (read filtering, read mapping, nucleotide‐wise coverage calculation, and genome feature‐wise counting) were done using READemption (Förstner *et al*, 2014) (v0.4.3; https://doi.org/10.5281/zenodo.250598) and the short read mapper segemehl (Hoffmann *et al*, 2014) (v0.2.0‐418). The *S. pneumoniae* TIGR4 genome version was NC_003028.3. Grad‐seq‐specific data analysis was performed with the tool GRADitude (Di Giorgio S, Hör J, Vogel J, Förstner KU, unpublished, https://foerstner-lab.github.io/GRADitude/). For the downstream analysis, only transcripts with a sum of ≥ 100 reads in all fractions within the gradient were considered. For each fraction, read counts were normalized by calculating size factors following the DESeq2 approach (Anders & Huber, 2010) generated from the ERCC spike‐in read counts added previously (see above). To remove left‐over disturbances in the data, the size factors were then manually adjusted by multiplication based on quantified northern blots: 1.15 (fraction 3), 1.15 (fraction 4), 0.85 (fraction 5), 0.95 (fraction 6), 0.85 (fraction 7), and 1.25 (fraction 15). To allow comparison of all transcript counts, they were scaled to the maximum value.

Based on these normalized values, two analyses were performed: one containing all the detectable transcripts, and one containing only the detectable ncRNAs. For the ncRNAs, t‐SNE dimension reduction (van der Maaten & Hinton, 2008) and k‐means clustering (Lloyd, 1982) were performed using the Python package scikit‐learn (Pedregosa *et al*, 2011). t‐SNE was performed excluding the pellet fraction using all the default parameters provided by the sklearn.manifold.TSNE class. The only exception was the perplexity parameter, set to 35 for Fig 3B and to 25 for Appendix Fig S4 instead of the default of 30. k‐means clustering was performed using the class sklearn.cluster.KMeans, grouping the ncRNAs into three clusters. Subsequently, the ncRNAs in the t‐SNE plot were colored according to their cluster number. Appendix Fig S4 shows stronger separation of the clusters by adding information of the k‐means analysis to the read count table before the t‐SNE dimensionality reduction. A script representing the analysis workflow, including Unix Shell calls and Python scripts, and documentation have been deposited at Zenodo (https://doi.org/10.5281/zenodo.3475892).

### Sample preparation for mass spectrometry

For mass spectrometry (MS), the gradient protein samples (diluted in 1.25× protein loading buffer) were homogenized using ultrasound [five cycles of 30 s on followed by 30 s off, high power at 4°C (Bioruptor Plus, Diagenode)]. Insoluble material was then removed by centrifugation for 15 min at 4°C and 16,100 *g*. 20 μl of the cleared protein sample was mixed with 10 μl of UPS2 spike‐in (Sigma‐Aldrich) diluted in 250 μl 1.25× protein loading buffer. The samples were subsequently reduced in 50 mM DTT for 10 min at 70°C and alkylated with 120 mM iodoacetamide for 20 min at room temperature in the dark. The proteins were precipitated in four volumes of acetone overnight at −20°C. Pellets were washed four times with acetone at −20°C and dissolved in 50 μl 8 M urea, 100 mM ammonium bicarbonate.

Digestion of the proteins was performed by the addition of 0.25 μg Lys‐C (Wako) for 2 h at 30°C, followed by dilution to 2 M urea by the addition of 150 μl 100 mM ammonium bicarbonate, pH 8 and overnight digestion with 0.25 μg trypsin at 37°C. Peptides were desalted using C‐18 Stage Tips (Rappsilber *et al*, 2003). Each Stage Tip was prepared with three disks of C‐18 Empore SPE Disks (3M) in a 200‐μl pipette tip. Peptides were eluted with 60% acetonitrile/0.3% formic acid, dried in a laboratory freeze‐dryer (Christ), and stored at −20°C. Prior to nanoLC‐MS/MS, the peptides were dissolved in 2% acetonitrile/0.1% formic acid.

### NanoLC‐MS/MS analysis

NanoLC‐MS/MS analysis was performed similarly to Cossa *et al* (2020) using an Orbitrap Fusion (Thermo Fisher Scientific) equipped with a PicoView Ion Source (New Objective) and coupled to an EASY‐nLC 1000 (Thermo Fisher Scientific). Peptides were loaded on capillary columns (PicoFrit, 30 cm × 150 μm ID, New Objective) self‐packed with ReproSil‐Pur 120 C18‐AQ, 1.9 μm (Dr. Maisch) and separated with a 140‐min linear gradient from 3 to 40% acetonitrile and 0.1% formic acid at a flow rate of 500 nl/min. Both MS and MS/MS scans were acquired in the Orbitrap analyzer with a resolution of 60,000 for MS scans and 15,000 for MS/MS scans. HCD fragmentation with 35% normalized collision energy was applied. A Top Speed data‐dependent MS/MS method with a fixed cycle time of 3 s was used. Dynamic exclusion was applied with a repeat count of 1 and an exclusion duration of 60 s; singly charged precursors were excluded from selection. Minimum signal threshold for precursor selection was set to 50,000. Predictive AGC was used with a target value of 2 × 10^5^ for MS scans and 5 × 10^4^ for MS/MS scans. EASY‐IC was used for internal calibration.

### Grad‐seq MS data analysis

Raw MS data files were analyzed with MaxQuant version 1.5.7.4 (Cox & Mann, 2008). Database search was performed using Andromeda (integrated into MaxQuant) against the UniProt database for *S. pneumoniae* TIGR4 (UP000000585, organism identifier: STRPN), a database containing the UPS2 spike‐in and a database containing common contaminants. The search was performed with tryptic cleavage specificity with three allowed miscleavages. Protein identification was under control of a false discovery rate of 1% on both protein and peptide levels. In addition to the MaxQuant default settings, the search was performed against the following variable modifications: protein N‐terminal acetylation, Gln to pyro‐Glu formation (N‐terminal Gln), and oxidation of Met. For protein quantitation, the LFQ intensities were used (Cox *et al*, 2014). Proteins with less than 2 identified razor/unique peptides were dismissed.

Normalization of the proteins across the fractions was performed using the UPS2 spike‐in. For this, only spike‐in proteins with detectable intensities in all fractions were used. The spike‐in proteins showing the highest variance (median average deviation of log_10_ intensities > 1.5× lQR) were eliminated. Following this, for each spike‐in protein, the median log_10_ intensity was subtracted from the log_10_ intensities of each fraction. The fraction‐wise median of the resulting values was then subtracted from the log_10_ intensities for each bacterial protein in the corresponding fractions. Finally, all log_10_ intensities smaller than the 5% quantile of all intensities in the dataset were replaced by the value of the 5% quantile of all intensities in the dataset.

### Protein pull‐down using RNA as bait

To pull down proteins using RNA as bait, a modified version of a published protocol (Treiber *et al*, 2017, 2018) was used. PCR templates for *in vitro* transcription with T7 RNAP were generated with a 39 nt 5′ overhang: GTTTTTTT*TAATACGACTCACTATA* ***G*** ***GG*** **AGACCTAGCCT** (italicized nucleotides belong to the T7 promoter, bold nucleotides represent the 14 nt 5′ tag, the underlined nucleotide is the transcription start site). Primers used are listed in Table EV2. *In vitro* transcription was performed using the TranscriptAid kit (Thermo Fisher Scientific) according to the manufacturer's instructions. The resulting RNA was purified using denaturing 6% PAGE in 1× TBE and 7 M urea. An RNA oligonucleotide representing only the 14 nt tag (GGGAGACCUAGCCU) was used as negative control.

For the pull‐down, 100 μl magnetic streptavidin beads (Dynabeads M‐270, Thermo Fisher Scientific) were washed 3× with 1 ml of lysis buffer B (50 mM Tris–HCl, pH 8, 150 mM KCl, 1 mM MgCl_2_, 5% glycerol, 0.05% Tween‐20). The rest of the protocol was performed at 4°C. The washed beads were coupled to 4 μg of a 3′‐biotinylated, 2′‐O‐methyl‐modified RNA adaptor complementary to the 14 nt tag of the bait RNAs (AGGCUAGGUCUCCC‐biotin) for 1 h. The adaptor‐coupled beads were washed twice with 1 ml of lysis buffer B, resuspended in 1 ml of lysis buffer B, and split into two tubes with 500 μl each. One tube was used to couple 10 μg per 100 nt of bait RNA overnight, and the other was stored for pre‐clearing.

To prepare the lysate for the pull‐down, 100 OD_600 nm_ of cells were lysed and cleared in 500 μl lysis buffer C (lysis buffer B with 1 mM DTT and 1 mM PMSF) as described above. To pre‐clear the lysate from content that binds the beads unspecifically, the stored beads were incubated with the lysate for 3.5 h. The beads were subsequently removed by centrifugation for 10 min at 16,100 *g*. The bait RNA‐coupled beads were washed twice with lysis buffer B and incubated with the pre‐cleared lysate supernatant for 2 h to capture interacting proteins of the bait RNAs. To get rid of unspecific binders, the beads were washed with 1 ml each of wash buffer A (lysis buffer C with 300 mM KCl in total), wash buffer B (lysis buffer C with 0.1% Triton X‐100), and lysis buffer C.

The beads were resuspended in 35 μl of 1× LDS sample buffer (Thermo Fisher Scientific) containing 50 mM DTT and boiled for 5 min at 95°C to elute the bound proteins. Following alkylation as described above, the pull‐down samples were run on a 4–12% Bolt Bis‐Tris plus gel (Thermo Fisher Scientific) using MES buffer (Thermo Fisher Scientific). After staining with SimplyBlue Coomassie (Thermo Fisher Scientific), each lane of the gel was either cut into 11 pieces or specific prominent bands were cut. The gel pieces were prepared for LC/MS‐MS by destaining with 30% acetonitrile in 100 mM ammonium bicarbonate, pH 8 followed by shrinking with 100% acetonitrile and drying in a vacuum concentrator (Eppendorf). 0.1 μg trypsin was added per gel piece and digestion performed overnight at 37°C in 100 mM ammonium bicarbonate, pH 8. The supernatant was removed, and the peptides were extracted from the gel pieces with 5% formic acid. Finally, the supernatant was pooled with the extracted peptides.

### Pull‐down nanoLC‐MS/MS analysis

NanoLC‐MS/MS analyses were performed similarly to Braun *et al* (2018) on an LTQ‐Orbitrap Velos Pro (Thermo Fisher Scientific) equipped with a PicoView Ion Source (New Objective) and coupled to an EASY‐nLC 1000 (Thermo Fisher Scientific). Peptides were loaded on capillary columns (PicoFrit, 30 cm × 150 μm ID, New Objective) self‐packed with ReproSil‐Pur 120 C18‐AQ, 1.9 μm (Dr. Maisch) and separated with a 30‐min linear gradient from 3 to 30% acetonitrile and 0.1% formic acid at a flow rate of 500 nl/min. MS scans were acquired in the Orbitrap analyzer with a resolution of 30,000 at m/z 400, and MS/MS scans were acquired in the Orbitrap analyzer with a resolution of 7,500 at m/z 400 using HCD fragmentation with 30% normalized collision energy. A TOP5 data‐dependent MS/MS method was used; dynamic exclusion was applied with a repeat count of 1 and an exclusion duration of 30 s; singly charged precursors were excluded from selection. Minimum signal threshold for precursor selection was set to 50,000. Predictive AGC was used with a target value of 1 × 10^6^ for MS scans and 5 × 10^4^ for MS/MS scans. Lock mass option was applied for internal calibration in all runs using background ions from protonated decamethylcyclopentasiloxane (m/z 371.10124).

### Pull‐down MS data analysis

Analysis of the pull‐down MS data was performed as described for the Grad‐seq MS data, except that the search against the UPS2 spike‐in database was skipped.

### UV CLIP‐seq sample preparation and sequencing

Sample preparation for UV crosslinking and immunoprecipitation followed by RNA‐seq (CLIP‐seq) followed a previously published protocol (Holmqvist *et al*, 2018) with a few changes. For each biological replicate, 800 ml of the *cbf1*‐3xFLAG strain was grown to an OD_600 nm_ of 0.5. Half of the culture was irradiated in 50 ml fractions at 254 nm in a 22 × 22 cm plastic tray at 0.8 J. After collection of the cells by centrifugation at 4°C, the pellets were frozen in liquid nitrogen and stored at −80°C. Cells were thawed on ice, resuspended in 800 μl NP‐T buffer (50 mM NaH_2_PO_4_, 300 mM NaCl, 0.05% Tween‐20, pH 8), and transferred to tubes containing 1 ml of glass beads. Following lysis for 10 min at 30 Hz using a mixer mill (Retsch MM400), the lysates were cleared twice by centrifugation for 15 min at 16,100 *g* and 4°C and mixed with one volume of NP‐T buffer containing 8 M urea. Finally, the lysates were incubated for 5 min at 65°C with shaking at 900 rpm, put on ice for 2 min and diluted 10× in ice‐cold NP‐T buffer, and put back on ice.

To perform the immunoprecipitation, 30 μl of anti‐FLAG magnetic beads was washed 3× with 800 μl NP‐T buffer, added to the lysate, and rotated for 1 h at 4°C. The beads were collected by centrifugation at 1,000 *g* and 4°C, and washed twice with 2 ml of high‐salt buffer (NP‐T buffer containing 1 M NaCl) and twice with 2 ml of NP‐T buffer. Subsequently, the beads were resuspended in 100 μl NP‐T buffer containing 1 mM MgCl_2_ and 2.5 U benzonase (Sigma) followed by incubation for 10 min at 37°C with shaking at 800 rpm. Then, the beads were put on ice for 2 min and washed once with 500 μl high‐salt buffer and twice with 500 μl CIP buffer (100 mM NaCl, 50 mM Tris–HCl, pH 7.4, 10 mM MgCl_2_) followed by resuspension in 100 μl CIP buffer containing 10 U of calf intestinal alkaline phosphatase (NEB) and incubation for 30 min at 37°C with shaking at 800 rpm. The beads were washed once with 500 μl high‐salt buffer and twice with 500 μl PNK buffer (50 mM Tris–HCl, pH 7.4, 10 mM MgCl_2_, 0.1 mM spermidine) followed by resuspension in 100 μl PNK buffer containing 1 μl T4 polynucleotide kinase (Thermo Fisher Scientific) and 1 μl γ‐^32^P‐ATP for 30 min at 37°C. Then, 10 μl 1 mM ATP was added and the beads were incubated for 5 min at 37°C, followed by two washes with 1 ml of NP‐T buffer and resuspension in 10 μl protein loading buffer. The labeled RNA–protein complexes were eluted from the beads by incubation for 5 min at 95°C, which was repeated once.

15 μl of the eluted samples was separated using 12% SDS–PAGE followed by transfer to a nitrocellulose membrane (GE Healthcare Life Sciences). Marker sizes of the protein ladder were highlighted using a radioactive marker, and the autoradiogram of the blot was subsequently used as a template to cut out the labeled complexes and their corresponding controls. The membrane pieces were then cut into smaller pieces, transferred to LoBind tubes (Eppendorf), and incubated with 200 μl PK buffer [50 mM Tris–HCl, pH 7.4, 75 mM NaCl, 6 mM EDTA, 1% SDS, 10 U SUPERaseIN (Thermo Fisher Scientific), and 0.4 mg proteinase K (Thermo Fisher Scientific)] for 1 h at 37°C with shaking at 800 rpm. Then, 100 μl PK buffer containing 9 M urea was added and the incubation continued for 1 h. The membrane pieces were briefly centrifuged and the supernatant extracted with one volume of P/C/I. The purified RNA fragments were resuspended in 10 μl DEPC‐treated H_2_O.

Library preparation for next‐generation sequencing was performed by the CoreUnit SysMed at the University of Würzburg, Germany. The protocol used is the same as published before (Holmqvist *et al*, 2018). Sequencing was also performed by the CoreUnit SysMed on an Illumina NextSeq 500 system using 2 × 75 nt paired‐end sequencing.

### CLIP‐seq data analysis

CLIP‐seq data analysis was performed as described previously (Chihara *et al*, 2019). Briefly, read 1 and read 2 files containing the paired‐end reads were quality and adapter trimmed via Cutadapt (Martin, 2011), version 1.17, using a cutoff Phred score of 20 in NextSeq mode, and reads without any remaining bases were discarded. Putative PCR duplicates were discarded by collapsing the reads using FastUniq (Xu *et al*, 2012). After trimming, all reads longer than 11 nt were aligned to the *S. pneumoniae* TIGR4 genome (NC_003028.3) using READemption (Förstner *et al*, 2014) (version 0.4.5) and segemehl (Hoffmann *et al*, 2014) (version 0.2.0) with an accuracy cutoff of 80%. Only reads uniquely mapping to the genome were considered for all subsequent analyses.

Size factors for normalization of the CLIP‐seq data were calculated based on positional coverage files generated via READemption. In brief, positions present in both crosslinked and non‐crosslinked library pairs were isolated, as described previously (Holmqvist *et al*, 2018). Positions with read counts less than 6 standard deviations from 0 were filtered from both crosslinked and non‐crosslinked libraries. The distribution of ratios between the paired crosslinked and non‐crosslinked samples presented a clear bimodal distribution, with modes corresponding to background (high counts in both libraries) and crosslink‐enriched positions, which were separated by k‐means clustering. Size factors were then calculated from the background positions present across all replicates against a geometric mean pseudo‐reference, as introduced in DESeq (Anders & Huber, 2010).

We applied PEAKachu (https://github.com/tbischler/PEAKachu) (version 0.1.0) for peak calling in a similar way as described previously (Holmqvist *et al*, 2018). The tool was run in paired‐end and paired‐replicates mode using BAM files for the respective pairs of crosslinked and control libraries as input. The maximum fragment size was set to 50, and the *S. pneumoniae* TIGR4 annotation (Warrier *et al*, 2018) was used to map overlapping features to called peaks. For normalization, “manual” mode was selected together with previously calculated size factors (see above). Peak calling via the adaptive approach is performed in two consecutive steps. In the first step, initial peaks are defined via heuristic decomposition of read clusters computed by the blockbuster algorithm (Langenberger *et al*, 2009) based on pooled read alignments from all crosslinked libraries. In this step, PEAKachu applies a set of parameters for which default values were used. In the second step, PEAKachu runs DESeq2 (Love *et al*, 2014) to test each peak for significant enrichment of normalized read counts in the crosslinked compared to the control libraries. Initial peaks were tested for significance using the following parameter values: mad‐multiplier 1.0, fold change 1.0, and adjusted *P*‐value 0.01. Normalized coverage plots representing the numbers of mapped reads per nt were generated by PEAKachu for each strand to facilitate data visualization in a genome browser.

### Purification of recombinant Cbf1

The purification of recombinant Cbf1 was performed by the Recombinant Protein Expression core unit at the Rudolf Virchow Center in Würzburg, Germany. Briefly, to purify recombinantly expressed Cbf1, the CDS was cloned into pet21b+, which adds a C‐terminal His‐tag to the sequence (N‐terminal His‐tagging did not allow purification of Cbf1). After transformation into *E. coli* BL21, cells were grown to an OD_600 nm_ of 0.5 and induced with 0.5 mM IPTG for 3 h at 37°C. After centrifugation, the cell pellet was dissolved in 20 mM HEPES, pH 8, 250 mM NaCl, 0.4 mM PMSF, and 1.5 U/ml DNase I and subjected to affinity purification using Protino Ni‐NTA Agarose (Macherey Nagel). Following elution, the purity was tested on a Superdex 16/600 column (GE Healthcare Life Sciences). Purified Cbf1 was stored in 100 μl aliquots of 20 mM HEPES, pH 8, 250 mM NaCl, and 10% glycerol at −80°C (~ 1.35 mg/ml).

### *In vitro* RNase assay

To test the activity of Cbf1 on target RNAs, 400 ng *in vitro*‐transcribed RNAs (~ 9–14 pmol, depending on the RNA) were digested with 3.5 μg Cbf1 (~ 100 pmol) in a total volume of 20 μl [50 mM Tris–HCl, pH 8, 100 mM KCl; according to Fang *et al* (2009)]. Primers used are listed in Table EV2. Reactions contained either no divalent cations or 5 mM of MgCl_2_ or MnCl_2_. Samples (10% of the reaction volume) were taken immediately after addition of Cbf1 (time point 0 min) and then, after shifting the reaction to 37°C, after 5 and 15 min. Collection was performed on ice in RNA loading buffer and immediately frozen in order to stop the reaction. The samples were analyzed by northern blotting using denaturing 10% PAGE.

### 3′ RACE

Identification of 3′ ends via 3′ rapid amplification of cDNA ends (RACE) was performed as described before (Fröhlich *et al*, 2012) with few modifications. First, a Cbf1 *in vitro* RNase assay with truncated csRNA1 was performed as described above, except that the total reaction volume was scaled up to 200 μl containing 4 μg of RNA. 50 μl of the reaction (1 μg of RNA) was taken at the indicated time points and stopped by addition of 150 μl acidic P/C/I and 200 μl of H_2_O. As negative control, 1 μg of untreated RNA was used. After P/C/I extraction, the RNA was dephosphorylated using 10 U of calf intestinal alkaline phosphatase (NEB) in 1× NEB buffer 3 in 25 μl for 1 h at 37°C. The RNA was then P/C/I‐extracted and precipitated in the presence of 1 μl GlycoBlue (Thermo Fisher Scientific) and 250 pmol of RNA adapter E1. Ligation of the adapter was performed in a 20 μl reaction containing 20 U T4 RNA ligase (NEB), 1× T4 RNA ligase buffer, and 10% DMSO overnight at 16°C. After ligation, the RNA was P/C/I‐extracted and reverse transcribed for 5 min at 50°C followed by 60 min at 55°C using 200 U SuperScript III Reverse Transcriptase (Thermo Fisher Scientific) and 39 pmol of adapter E1‐specific oligo E3RACE in a 20 μl reaction containing 1× FS buffer, 2 mM dNTPs, and 5 mM DTT. The template RNA was digested by addition of 1 μl RNase H (NEB) and incubation at 37°C for 30 min. 1 μl of the cDNA was then used as template for a Taq PCR using E3RACE and JVO‐15297 as primers. The PCR products were run on a 3.5% agarose gel, the bands cut from the gel and cloned using the StrataClone PCR cloning kit (Agilent Technologies) following the manufacturer's instructions. For the 5‐ and 15‐min time points, the inserts of 20 positive clones each were amplified by PCR using the primers M13 and M13rev and analyzed by Sanger sequencing. Primers used are listed in Table EV2.

### Rifampicin RNA stability assays

The *in vivo* stability of RNAs was determined by growing bacterial cultures in a water bath until they reached an OD_600 nm_ of 0.5. Four OD_600 nm_ were collected as the non‐treated 0‐min sample. Then, 500 μg/ml rifampicin was added to stop transcription and four OD_600 nm_ samples were collected after 2, 4, 8, 16, and 32 min. Each sample was immediately mixed with 0.2 volumes of stop‐mix (95% ethanol, 5% phenol) and snap‐frozen in liquid nitrogen. Following RNA isolation using the hot phenol method and DNase treatment, 5 μg of RNA was analyzed by northern blotting and quantified using ImageJ (Schneider *et al*, 2012). 5S rRNA was used as loading control.

### CSP induction assay

To determine the influence of CSP induction, the *cbf1‐3xFLAG* strain was grown in a water bath until it reached an OD_600 nm_ of 0.5. For determination of protein levels, 1 ml of culture was collected as the non‐treated 0‐min sample and immediately snap‐frozen in liquid nitrogen. For RNA purification, instead 4 ml of culture were mixed with 0.2 volumes of stop‐mix and snap‐frozen in liquid nitrogen. Stimulation was then performed by addition of CSP‐2 (AnaSpec) to a final concentration of 100 ng/ml. For the control, the same volume of sterile H_2_O was added instead. After 5, 10, 15 and 30 min of stimulation, samples were collected the same way as for the 0 min time point. Protein samples were thawed on ice and diluted in 200 μl protein loading buffer, and equal volumes (10 μl) were subjected to PAGE and Western blotting. RNA isolation was performed using the hot phenol method followed by DNase treatment. 15 μg of RNA was loaded on a 1.2% agarose gel, run, and stained with ethidium bromide to visualize 16S and 23S rRNAs as loading control. Finally, capillary blotting on a Hybond+ membrane was performed and the membranes probed with RNA‐specific radioactively labeled DNA oligonucleotides.

### RT–qPCR

For the determination of mRNA levels via RT–qPCR, RNA was extracted as described for the rifampicin RNA stability assays. Following PCR‐based analysis of potential gDNA contamination, RT–qPCR was carried out using the Power SYBR Green RNA‐to‐CT 1‐Step kit (Thermo Fisher Scientific) and a CFX96 system (Bio‐Rad). *gyrA* was used as the control gene. Data were analyzed using the comparative ΔΔ*C*_T_ method (Livak & Schmittgen, 2001). Primers used are listed in Table EV2.

### Strain construction

Primers used are listed in Table EV2. All PCRs were carried out using Phusion Flash High‐Fidelity PCR Master Mix (Thermo Fisher Scientific) following manufacturer's instructions. Strains TIGR4Δ*cbf1*::Sp, R6Δ*cbf1*::Sp, and TIGR4 *cbf1‐*3xFLAG‐Sp were constructed by homologous recombination in the genome of a cassette composed of a spectinomycin (Sp) resistance gene flanked with ~ 1,000 bp homologous to the regions bordering the region of interest. Cassettes were assembled by PCR assembly via overlapping regions in the primers used for amplifying individual fragments. Briefly, the upstream (up) and downstream (dwn) of TIGR4/R6 *cbf1* were amplified using primers cbf1DF/cbf1DJ1R (up) and cbf1DJ2F/cbf1DR (dwn), and TIGR4 or R6 gDNA as the template. The spectinomycin resistance (Sp) gene, along with its promoter and terminator, was amplified from plasmid pSP72::Sp (courtesy of P. Mellroth) using primers cbf1DJ1F and cbf1DJ2R. The final up‐Sp‐dwn cassette was amplified using primers cbf1DF/cbf1DR (TIGR4) and cbf1DF2/cbf1DR2 (R6) and transformed into competent TIGR4/R6 cells. The *cbf1‐*3xFLAG strain was constructed by assembling *cbf1* from TIGR4 gDNA, amplified with primers cbf1VF/cbf13xFLAGR, with the Sp‐dwn region of TIGR4Δ*cbf1*::Sp strain amplified with primers AbRFLAGF/cbf1DR. The final product was amplified using primers cbf1_nestedF/cbf1_nestedR and transformed into competent TIGR4 cells.

The *cbf1* deletion was complemented in TIGR4Δ*cbf1*::Sp using plasmid pJWV25 which integrates into the chromosome at the *bga* locus by homologous recombination between sequences flanking the insert (Eberhardt *et al*, 2009). Briefly, *cbf1* was cloned into pJWV25 under control of the zinc‐inducible promoter P_*czcD*_ using ligase‐independent cloning (Li *et al*, 2011). *cbf1* was amplified from TIGR4 gDNA with primers cbf1_pGG8F/cbf1_pGG7R, and pJWV25 was amplified using primers pGG7F/pGG8R. Products were digested with DpnI and co‐transformed into *E. coli* XL‐GOLD Ultracompetent cells (Stratagene) following manufacturer's instructions and selected on LB plates supplemented with 100 μg/ml ampicillin. The resulting vector, pGG8A, was purified using the QIAprep Spin Miniprep Kit (QIAGEN), and 300 ng of plasmid DNA was transformed into competent TIGR4Δ*cbf1*::Sp. All PCR products described above were verified on agarose gel and, when necessary, excised, and purified using the Wizard SV Gel and PCR Clean‐Up System (Promega). Transformations were performed as described below for induced competence using 100 ng/ml CSP‐1 (R6) or 100 ng/ml CSP‐2 (TIGR4). Transformant colonies were isolated on blood agar plates containing the appropriate antibiotics at the following concentration: spectinomycin, 200 μg/ml; tetracycline, 2 μg/ml (pGG8A). All strains constructed were confirmed by PCR followed by sequencing at Eurofins (Germany).

### Competence assays

For spontaneous competence assays, pre‐cultures of R6 (Ottolenghi & Hotchkiss, 1962) and R6Δ*cbf1*::Sp were grown in C+Y (Lacks & Hotchkiss, 1960), pH 8 at 37°C without shaking to an OD_620 nm_ of 0.5. The pre‐cultures were refreshed in the same medium to a starting OD_620 nm_ of 0.05 and grown at 37°C without shaking to an OD_620 nm_ of 0.13. 50 μl aliquots were diluted 1:10 in C+Y pre‐warmed at 30°C and incubated for 30 min at 30°C. 100 ng/ml of a 524‐bp PCR product encompassing the SmR‐*rpsL* allele (see below) was added, and incubation was continued at 30°C for 60 min, followed by 90 min at 37°C. Samples were serially diluted and plated on blood agar plates containing 150 μg/ml streptomycin to enumerate transformant colony‐forming units (CFU) and on blood agar plates without antibiotics to enumerate total viable CFU. Results were expressed as the percentage of total CFU resistant to streptomycin. The *rpsL* PCR product used in transformation experiments was amplified using primer pair rpsLF/101‐rpsLR, gDNA from an *S. pneumoniae* strain carrying a point mutation in the *rpsL* gene conferring resistance to streptomycin (Muschiol *et al*, 2017) as the template and Phusion Flash High‐Fidelity PCR Master Mix (Thermo Fisher Scientific) following manufacturer's instructions. The product was purified using the Wizard SV Gel and PCR Clean‐Up System (Promega) and visualized on a 1% agarose gel run in 1× TAE and stained with GelRed (Biotium).

## Author contributions

BH‐N, GG, JH, and JV conceived and designed the study. GG, JH, and L‐MH performed experiments. KUF, JH, and SDG analyzed RNA‐seq and MS data. AS and JTV performed MS and analyzed MS data. BH‐N and JV supervised the project. JH and JV wrote the manuscript.

## Conflict of interest

The authors declare that they have no conflict of interest.

## Supporting information



Appendix

Expanded View Figures PDF

Table EV1

Table EV2

Dataset EV1

Dataset EV2

Dataset EV3

Dataset EV4

Dataset EV5

Source Data for Appendix

Review Process File

Source Data for Figure 1

Source Data for Figure 3

Source Data for Figure 4

Source Data for Figure 5

Source Data for Figure 6

Source Data for Figure 7

## Data Availability

RNA‐seq data: GEO GSE145605 (https://www.ncbi.nlm.nih.gov/geo/query/acc.cgi?acc=GSE145605) MS data: PRIDE PXD015835 and PXD015842 (http://www.ebi.ac.uk/pride/archive/projects/PXD015835 and http://www.ebi.ac.uk/pride/archive/projects/PXD015842) Code used: Zenodo 250598 and 3475892 (https://doi.org/10.5281/zenodo.250598 and https://doi.org/10.5281/zenodo.3475892)
