# Supplementary material for: Grad‐seq in a Gram‐positive bacterium reveals exonucleolytic sRNA activation in competence control
Source: EMBO J. 2020 Mar 30;39(9):e103852. doi: 10.15252/embj.2019103852 (PMC7196914; doi:10.15252/embj.2019103852)

APPENDIX

Table of contents

Page 2. **Appendix Figure Legends**

Page 5. **References**

Page 6. **Appendix Figure S1.** Multiple sequence alignment of SP_1969.

Page 7. **Appendix Figure S2.** Multiple sequence alignment of SP_0007.

Page 8. **Appendix Figure S3.** Multiple sequence alignment of SP_2226.

Page 9. **Appendix Figure S4.** t-SNE analysis of ncRNAs detected by Grad-seq.

Page 10. **Appendix Figure S5.** MS results of the sRNA pull-downs.

Page 11. **Appendix Figure S6.** 3’ RACE of Cbf1 RNase assay on truncated csRNA1.

Page 12. **Appendix Figure S7.** Cbf1 expression after CSP stimulation.

APPENDIX FIGURE LEGENDS

Appendix Figure S1. Multiple sequence alignment of SP_1969.

Multiple sequence alignment of SP_1969 was performed by searching for homologs using PHMMER (Potter *et al*, 2018) followed by multiple sequence alignment using Clustal Omega (Madeira *et al*, 2019) and visualization using Jalview (Waterhouse *et al*, 2009). Residues with ≥50% identity are highlighted in a blue gradient. STRPN, *Streptococcus pneumoniae*. STRMT, *Streptococcus mitis*. STRSY, *Streptococcus suis*. STRA5, *Streptococcus agalactiae*. STRP1, *Streptococcus pyogenes*. LACLA, *Lactococcus lactis*. PAEPS, *Paenibacillus polymyxa*. BACSU, *Bacillus subtilis*. BACCR, *Bacillus cereus*. BACAN, *Bacillus anthracis*. STAEQ, *Staphylococcus epidermidis*. STAA8, *Staphylococcus aureus*. LISMO, *Listeria monocytogenes*. ENTFA, *Enterococcus faecalis*. ENTFC, *Enterococcus faecium*. LACPL, *Lactobacillus plantarum*. LEUCJ, *Leuconostoc carnosum*. CLOPE, *Clostridium perfringens*. CLOBH, *Clostridium botulinum*. 9LACT, *Aerococcus viridans*. ECOLI, *Escherichia coli*.

Appendix Figure S2. Multiple sequence alignment of SP_0007.

Multiple sequence alignment of SP_0007 was performed by searching for homologs using PHMMER (Potter *et al*, 2018) followed by multiple sequence alignment using Clustal Omega (Madeira *et al*, 2019) and visualization using Jalview (Waterhouse *et al*, 2009). Residues with ≥50% identity are highlighted in a blue gradient. STRPN, *Streptococcus pneumoniae*. STRMT, *Streptococcus mitis*. STRSY, *Streptococcus suis*. STRA5, *Streptococcus agalactiae*. STRP1, *Streptococcus pyogenes*. LACLA, *Lactococcus lactis*. PAEPS, *Paenibacillus polymyxa*. BACSU, *Bacillus subtilis*. BACCR, *Bacillus cereus*. BACAN, *Bacillus anthracis*. STAEQ, *Staphylococcus epidermidis*. STAA8, *Staphylococcus aureus*. LISMO, *Listeria monocytogenes*. ENTFA, *Enterococcus faecalis*. ENTFC, *Enterococcus faecium*. LACPL, *Lactobacillus plantarum*. LEUCJ, *Leuconostoc carnosum*. CLOPE, *Clostridium perfringens*. CLOBH, *Clostridium botulinum*. 9LACT, *Aerococcus viridans*. ECOLI, *Escherichia coli*.

Appendix Figure S3. Multiple sequence alignment of SP_2226.

Multiple sequence alignment of SP_2226 was performed by searching for homologs using PHMMER (Potter *et al*, 2018) followed by multiple sequence alignment using Clustal Omega (Madeira *et al*, 2019) and visualization using Jalview (Waterhouse *et al*, 2009). Residues with ≥50% identity are highlighted in a blue gradient. Note that no *E. coli* homolog could be identified for SP_2226. STRPN, *Streptococcus pneumoniae*. STRMT, *Streptococcus mitis*. STRSY, *Streptococcus suis*. STRA5, *Streptococcus agalactiae*. STRP1, *Streptococcus pyogenes*. LACLA, *Lactococcus lactis*. PAEPS, *Paenibacillus polymyxa*. BACSU, *Bacillus subtilis*. BACCR, *Bacillus cereus*. BACAN, *Bacillus anthracis*. STAEQ, *Staphylococcus epidermidis*. STAA8, *Staphylococcus aureus*. LISMO, *Listeria monocytogenes*. ENTFA, *Enterococcus faecalis*. ENTFC, *Enterococcus faecium*. LACPL, *Lactobacillus plantarum*. LEUCJ, *Leuconostoc carnosum*. CLOPE, *Clostridium perfringens*. CLOBH, *Clostridium botulinum*. 9LACT, *Aerococcus viridans*.

Appendix Figure S4. t-SNE analysis of ncRNAs detected by Grad-seq.

t-SNE analysis of all 141 ncRNAs detected by Grad-seq. The same data as in Fig 3B are shown, except that cluster information from the k-means clustering was added to the analysis (see materials and methods).

Appendix Figure S5. MS results of the sRNA pull-downs.

A–H. Log_{10} LFQ intensities of the corresponding bait sRNA + the control are plotted against the log_2 ratio between the corresponding sRNA versus the control. Proteins only detected in the pull-down samples were imputed with values close to the baseline in the control (ctrl_imputed) to allow calculation of the ratio. Proteins only detected in the control were not given pseudocounts in the pull-down samples and therefore omitted. Cut-offs for proteins displayed in Fig 3B (highlighted in yellow) were set to 5.5 for the log_{10} LFQ intensities and to 4 for the log_2 ratios (indicated by dotted lines). Cbf1 (highlighted in red) is one of the most abundant and enriched proteins specific for csRNA1–5 (A–E) and F41 (F), whereas it was not pulled down by the riboswitch RNA F20 (G) or tmRNA (H). The positive control tmRNA enriched its specific binding protein

SmpB (highlighted in blue). Proteins not considered specifically enriched are shown in gray.

Appendix Figure S6. 3’ RACE of Cbf1 RNase assay on truncated csRNA1.

A. Overexposure of Fig 5B, truncated csRNA1. Probing at the 3’ end of the RNA reveals slight unspecific degradation in the presence of Cbf1, independent of the presence of metal ions.

B. Same membrane as in (A), but probed for the 5’ end of truncated csRNA1. A ~45 nt RNA species can be detected that is released by manganese-dependent cleavage by Cbf1 (arrows).

C. 3.5% agarose gel showing the product of RT-PCR of truncated csRNA1 after cleavage by Cbf1. Note that the full-length product visible in the negative control and at 0 min includes a T7 promoter and the 3’ primer used for the RT, which increase the length of the PCR product. Due to the presence of a stem-loop structure at the 3’ end of the full-length RNA, ligation of the adapter was probably disfavored on this species, explaining the fading of this band for the 5 and 15 min time points.

D, E. After gel purification from the gel shown in (C), cloning and Sanger sequencing of the cleavage products after 5 (D) and 15 (E) min of incubation, most of the resulting 3’ ends were found to be at position 45 of truncated csRNA1. Note that the two G nucleotides present at the 5’ end had to be introduced during the *in vitro* transcription of the RNA and are not present in the wild-type csRNA1.

F. Secondary structure prediction of the 45 nucleotide species identified by 3’ RACE. The prediction was performed using RNAfold (Lorenz *et al*, 2011).

Appendix Figure S7. Cbf1 expression after CSP stimulation.

A. Quantitative analysis of western blots of Cbf1-3xFLAG expression after CSP stimulation. Data is based on three independent biological replicates.

B. Western blot of Cbf1-3xFLAG without stimulation. To exclude the observed increase in Cbf1-3xFLAG expression after CSP induction (Fig 5A) was due to cell growth during the time course, the experiment was repeated without CSP stimulation.

Only a minor increase in protein level was detected after 30 min. Fold changes were calculated based on three independent experiments. Additionally, total RNA was extracted and an agarose northern blot was run showing *comCDE* levels after mock stimulation with H_2O. A GAPDH-specific antiserum and ethidium bromide staining were used as loading controls for the western and northern blots, respectively.

C. qPCR of *cbf1* levels after 10 min of CSP stimulation. An increase in mRNA levels of ~2-fold was observed. 5S rRNA was used as internal control. The mean fold change based on five independent biological replicates with three technical replicates each is shown.

D. Percentage of transformants using R6 and R6Δ*cbf1* in a spontaneous transformation assay. The same data as in Fig 7B are shown, except that the data points belonging to the same replicate are connected by a line. Percentage SmR CFU is based on 5 independent experiments.

Error bars show SD from the mean. *p*-values were calculated using unpaired, two-tailed t-tests.

REFERENCES

Lorenz R, Bernhart SH, Höner Zu Siederdissen C, Tafer H, Flamm C, Stadler PF, Hofacker IL (2011) ViennaRNA Package 2.0. *Algorithms Mol Biol* 6: 26

Madeira F, Park YM, Lee J, Buso N, Gur T, Madhusoodanan N, Basutkar P, Tivey ARN, Potter SC, Finn RD *et al* (2019) The EMBL-EBI search and sequence analysis tools APIs in 2019. *Nucleic Acids Res* 47: W636-W641

Potter SC, Luciani A, Eddy SR, Park Y, Lopez R, Finn RD (2018) HMMER web server: 2018 update. *Nucleic Acids Res* 46: W200-W204

Waterhouse AM, Procter JB, Martin DMA, Clamp M, Barton GJ (2009) Jalview Version 2--a multiple sequence alignment editor and analysis workbench. *Bioinformatics* 25: 1189-1191

Appendix Fig S1

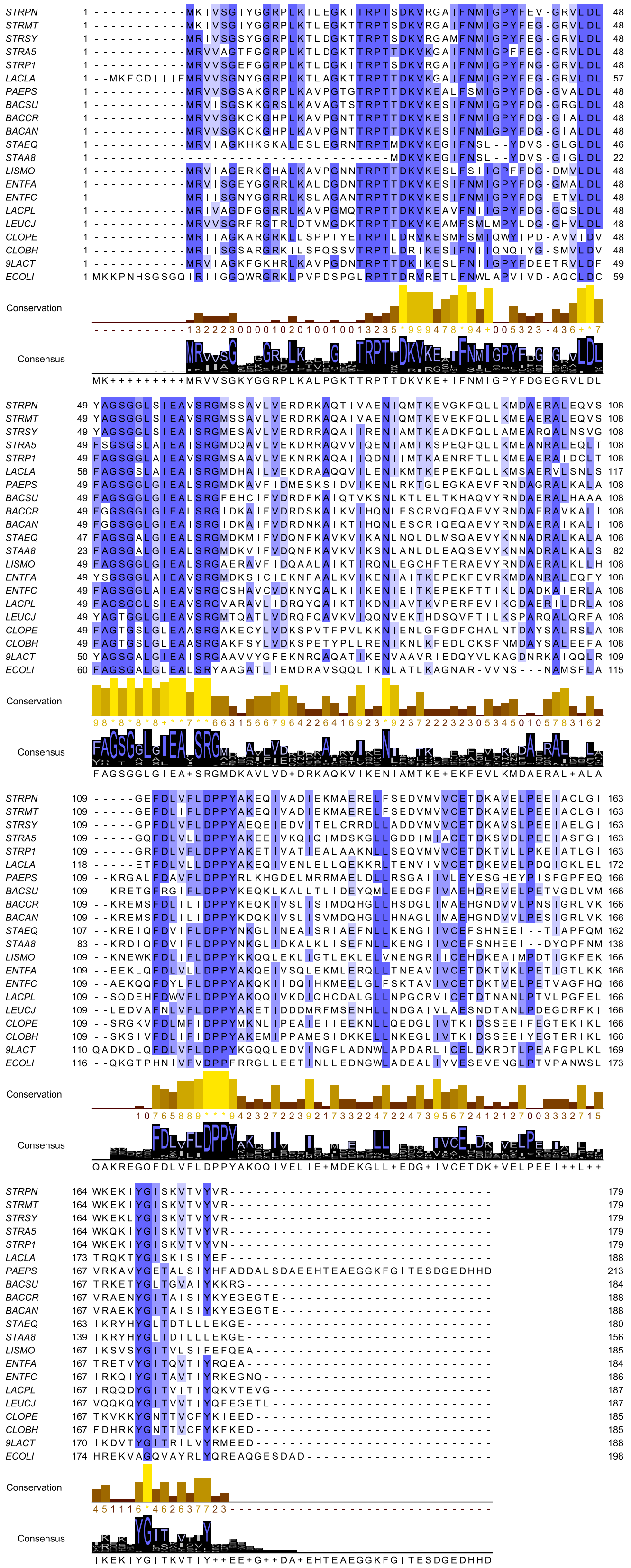

Appendix Fig S2

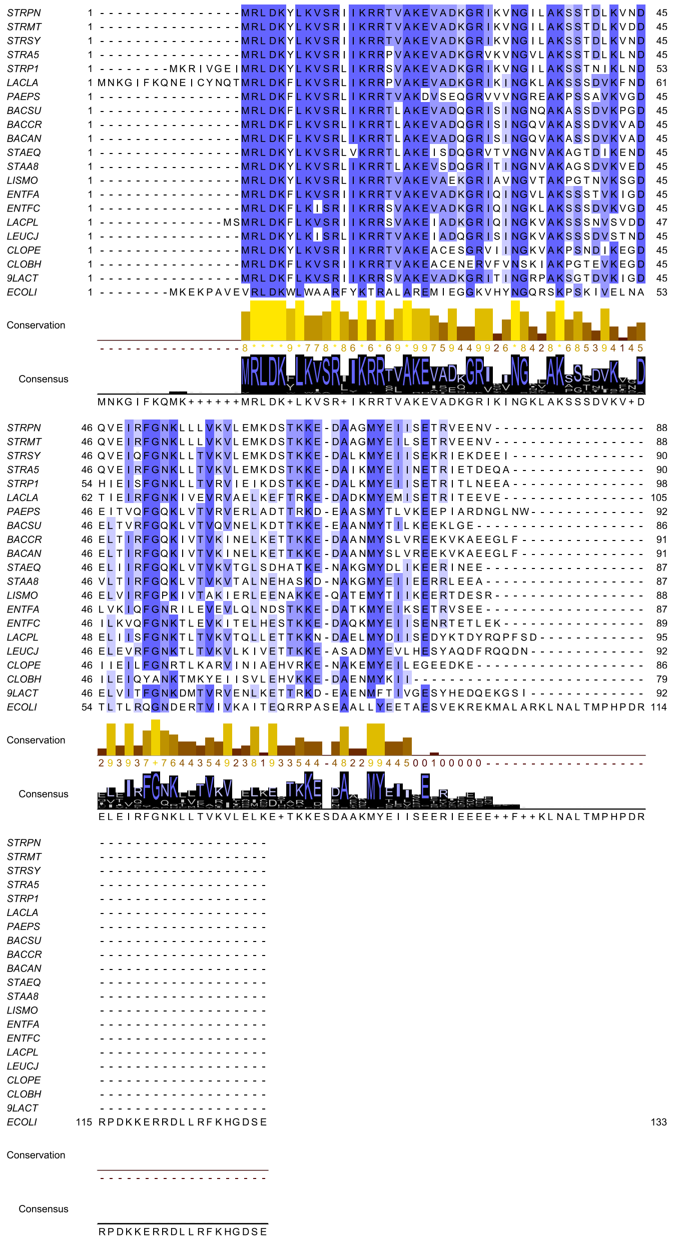

Appendix Fig S3

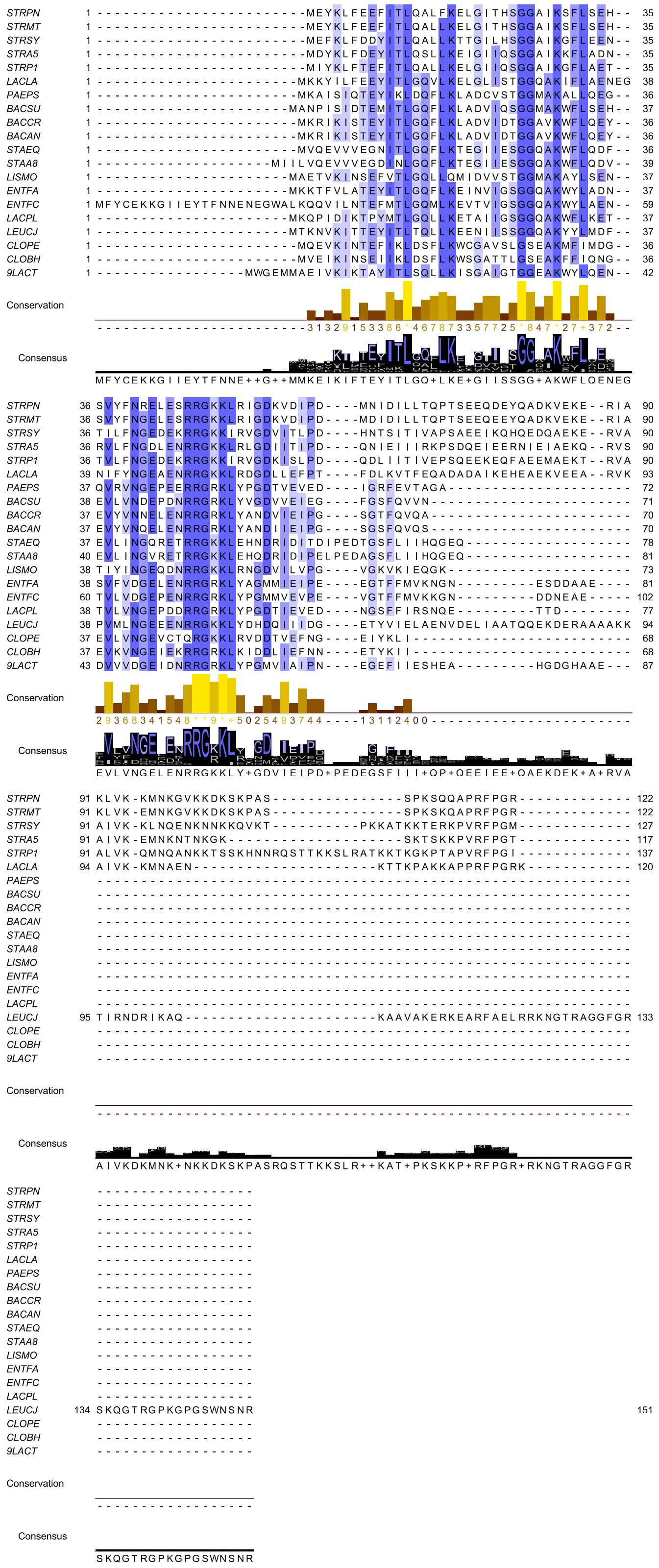

Appendix Fig S4

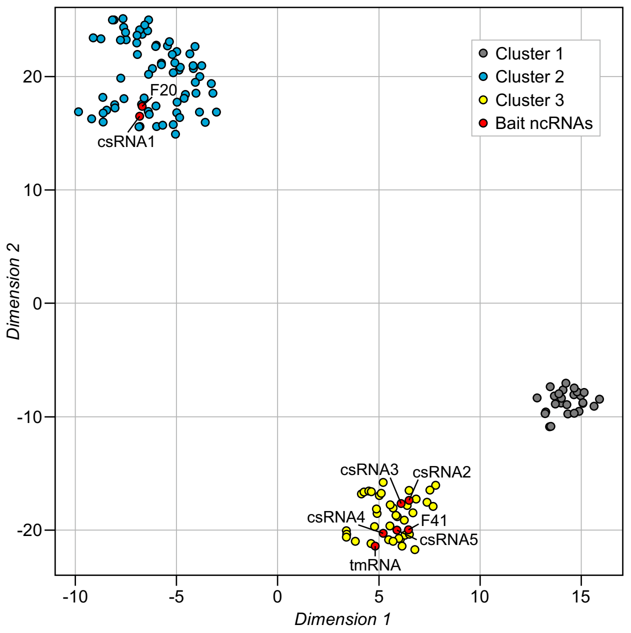

Appendix Fig S5

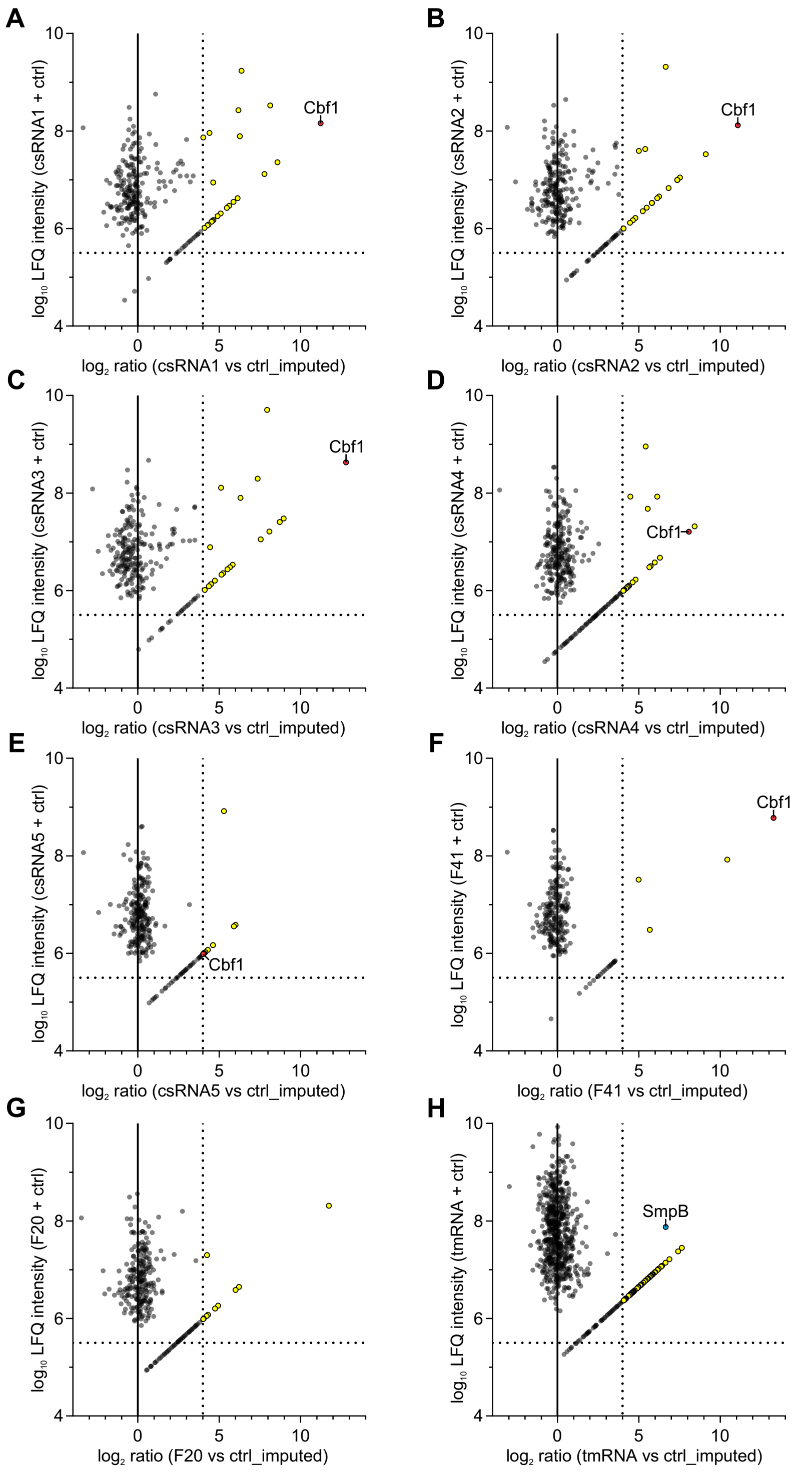

Appendix Fig S6

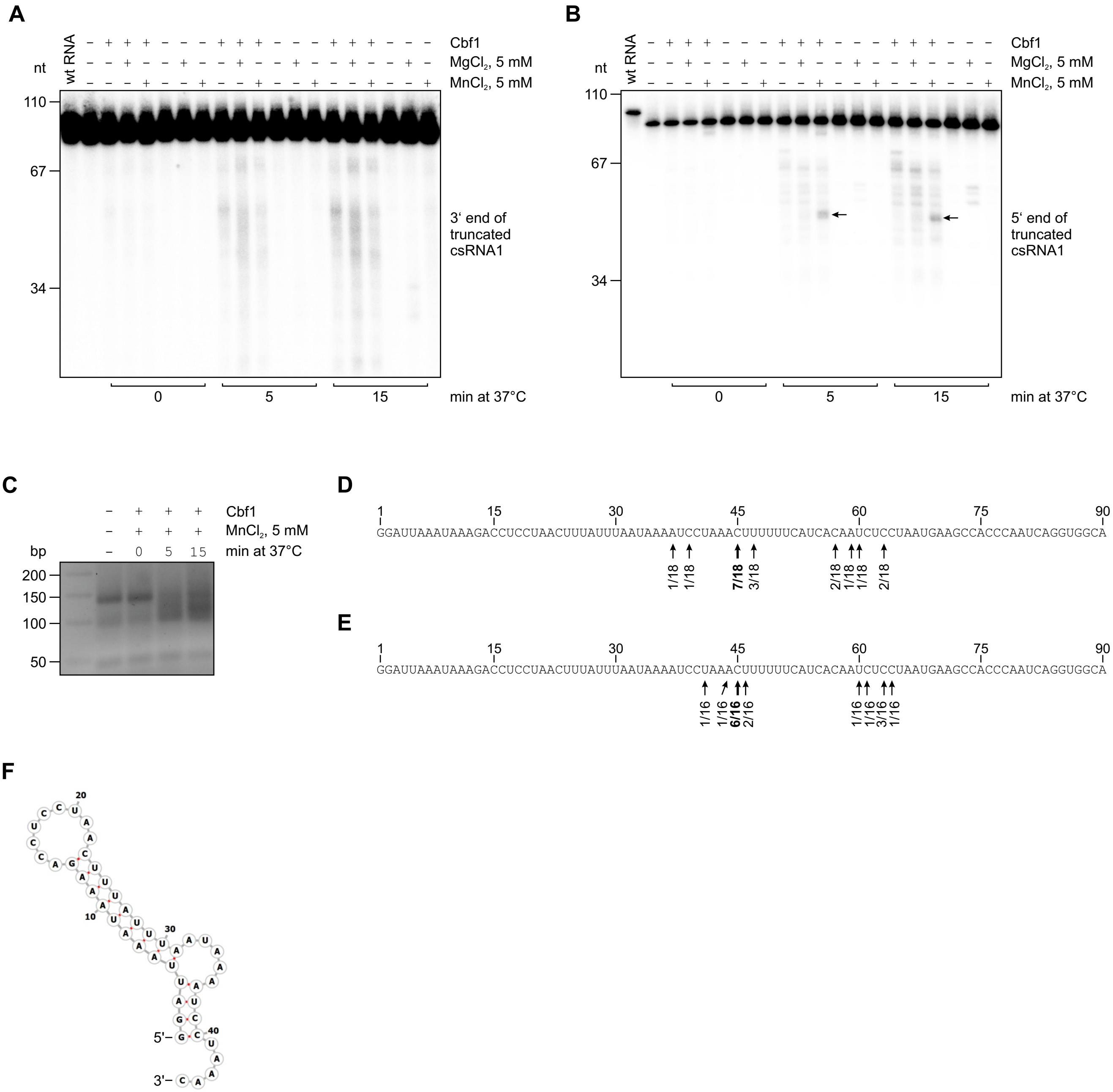

Appendix Fig S7

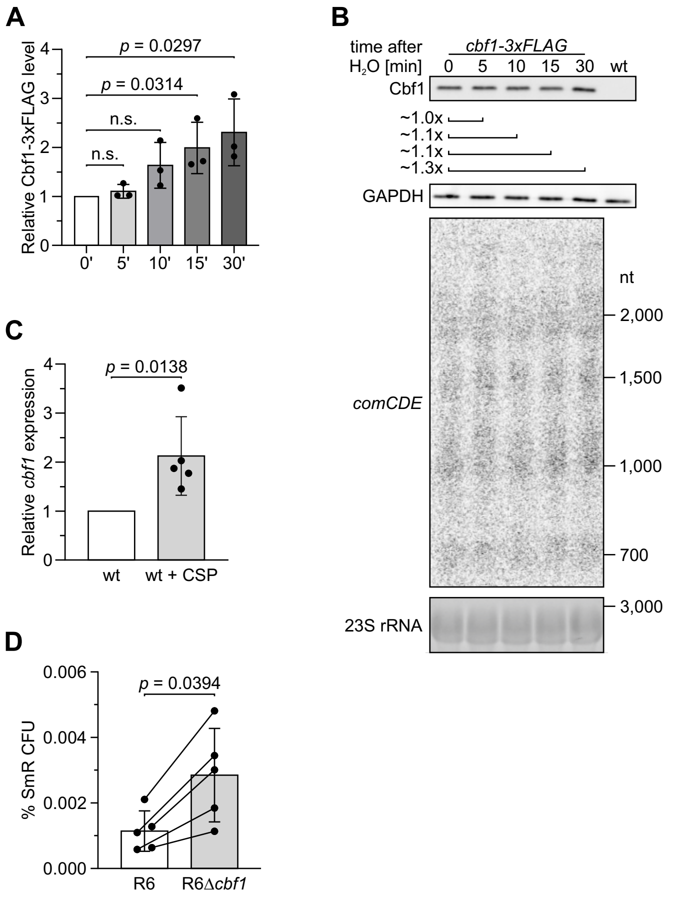

Supplement: Supplementary file 1 — Appendix [file EMBJ-39-e103852-s001.pdf]
