# Supplementary material for: Grad‐seq in a Gram‐positive bacterium reveals exonucleolytic sRNA activation in competence control
Source: EMBO J. 2020 Mar 30;39(9):e103852. doi: 10.15252/embj.2019103852 (PMC7196914; doi:10.15252/embj.2019103852)
Supplement: Supplementary file 2 — Expanded View Figures PDF [file EMBJ-39-e103852-s002.pdf]

Expanded View Figures

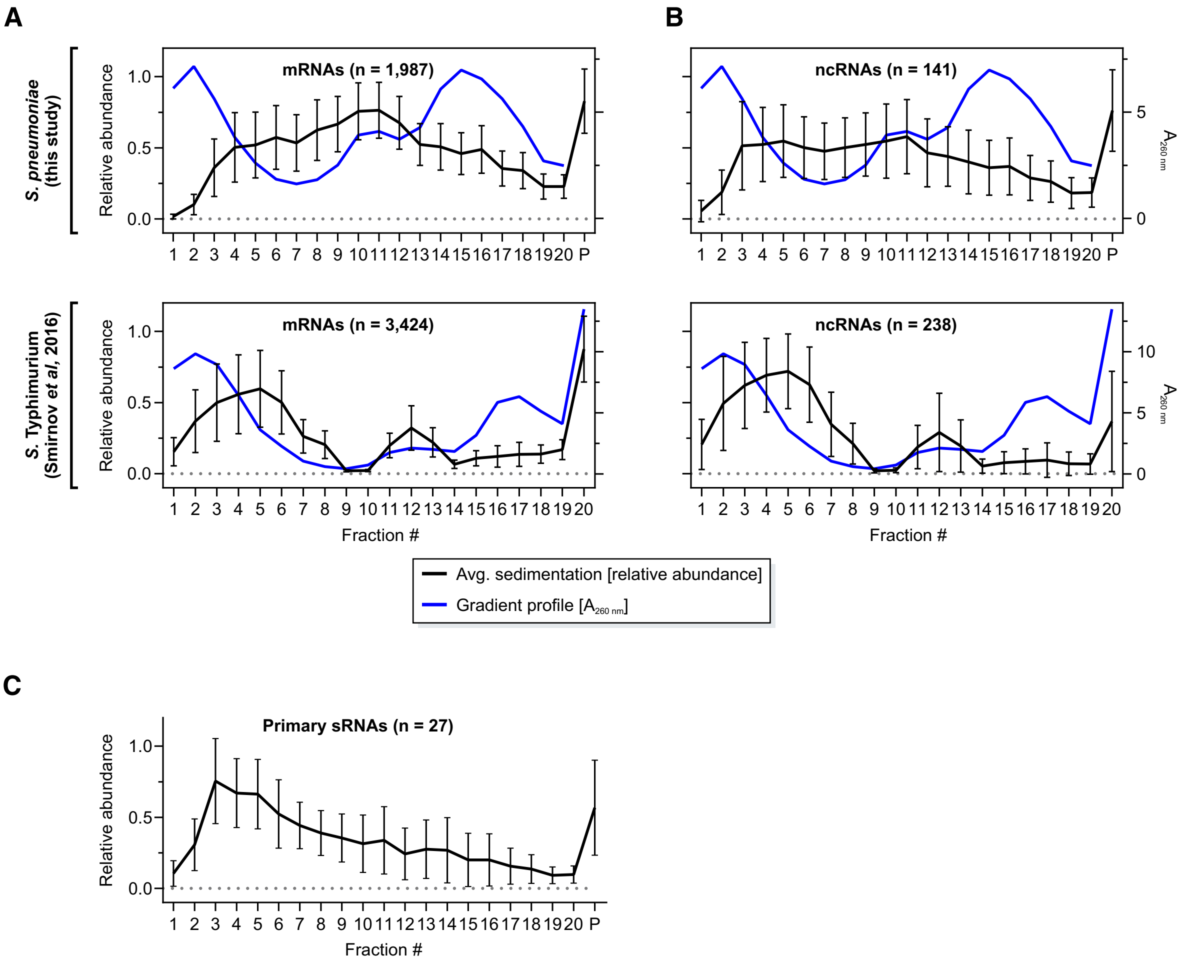

Figure EV1. Average Grad-seq transcript profiles.

A, B Average mRNA (A) and ncRNA (B) profiles of *Streptococcus pneumoniae* (upper panels, this study) and, for comparison, *S.* Typhimurium [lower panels, data from Smirnov *et al* (2016)]. As a reference, UV profiles of the gradients were added in blue [Fig 1B and Smirnov *et al* (2016)].

C Average profile of high confidence, independently transcribed sRNAs.

Data information: Error bars show SD from the mean.

 © 2020 The Authors

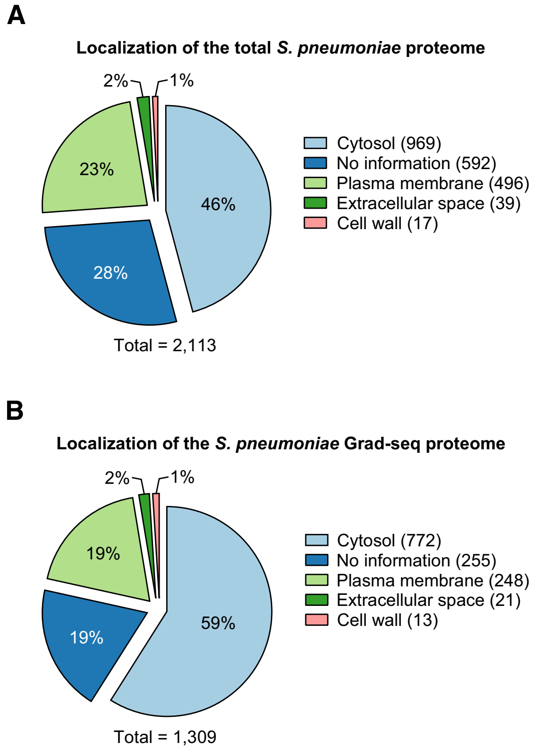

Figure EV2. Localization of detected proteins.

A, B Predicted localization of the total (A) and the Grad-seq (B) proteome of *Streptococcus pneumoniae*. Note that the numbers for (B) do not fit with Dataset EV2 because some proteins have more than one localization assigned. Predicted localizations were downloaded from BioCyc (Karp *et al*, 2019).

© 2020 The Authors

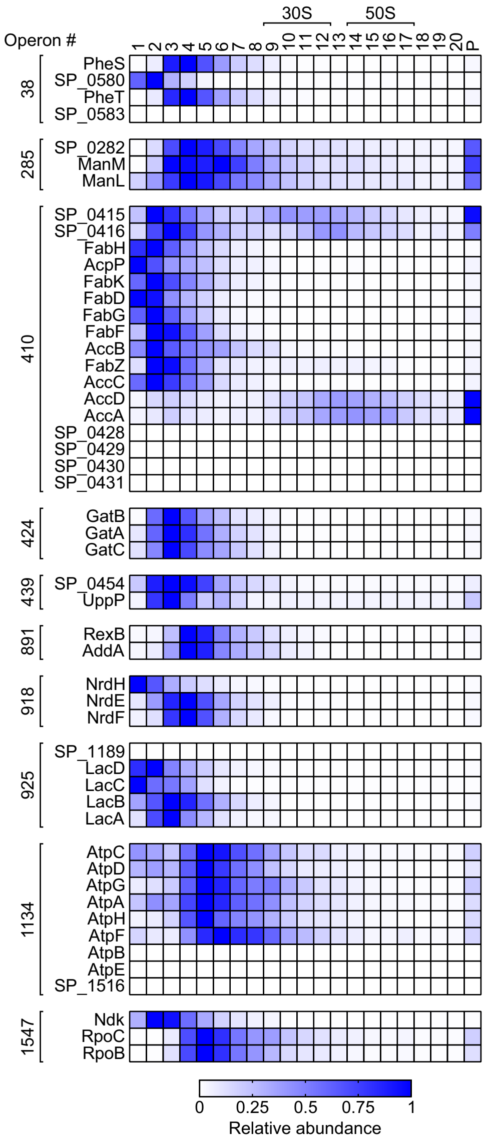

Figure EV3. Grad-seq profiles of proteins deriving from operons.
Exemplary operons [numbers according to Warrier *et al* (2018)] that provide evidence for predicted complexes.

© 2020 The Authors

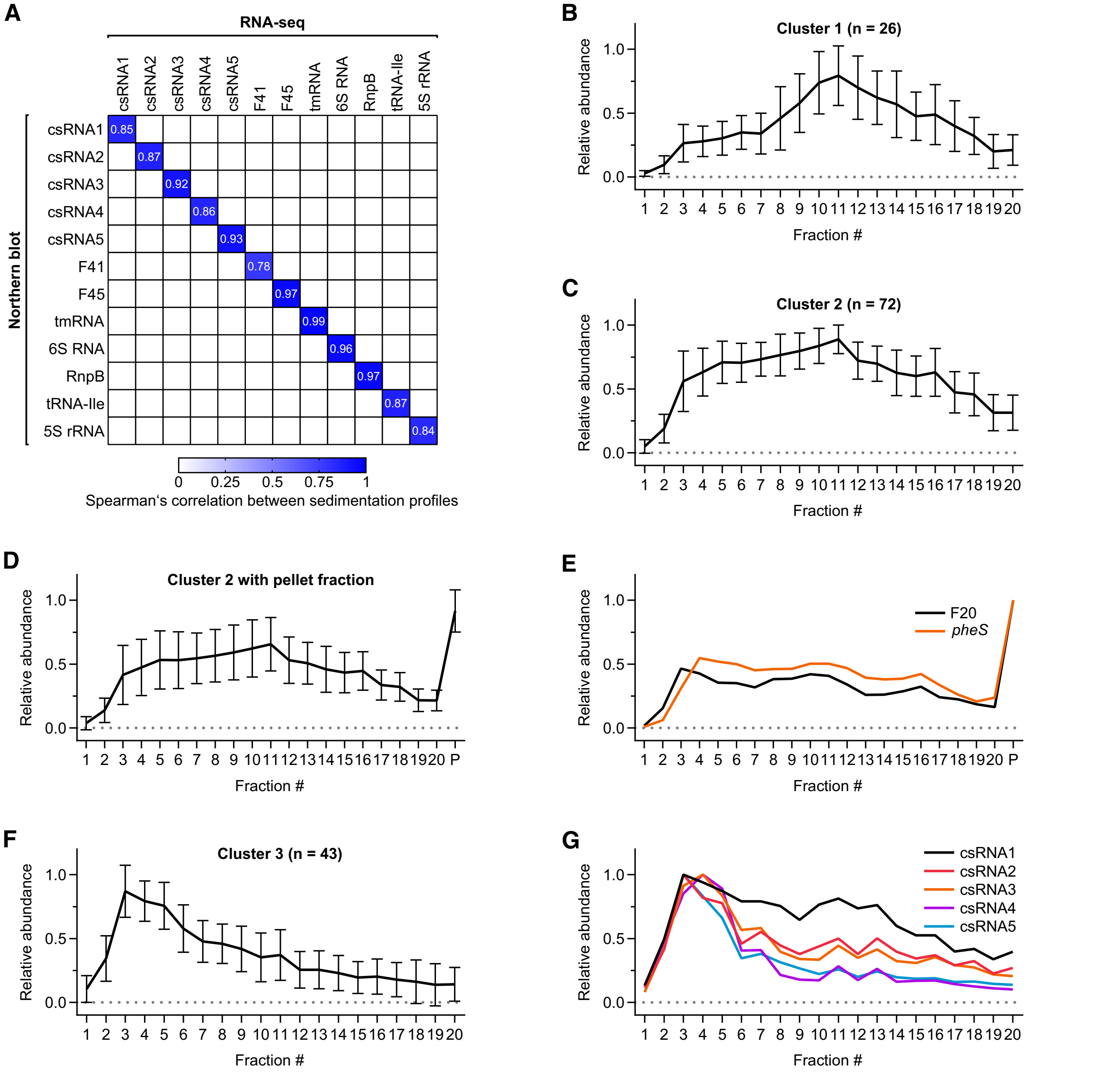

Figure EV4. Grad-seq profiles of ncRNAs.

A Heat map of Spearman's correlation between northern blot profiles (Fig 3A) and RNA-seq profiles for selected ncRNAs. Two-tailed *P*-values for all correlations are $2.77 \times 10^{-17} < P < 2.60 \times 10^{-5}$.

B–F Average ncRNA profiles for cluster 1 (B), cluster 2 (C), cluster 2 including the pellet fraction (D), and cluster 3 (F) show distinct sedimentation, separating them from each other (Fig 3B). Cluster 2 includes the riboswitch RNA F20, which has similar sedimentation to its downstream CDS *pheS* (E).

G RNA-seq profiles of the five csRNAs. Note that csRNA1 has a clear peak in fraction 3, albeit not as pronounced as the other csRNAs.

Data information: Error bars show SD from the mean.

© 2020 The Authors

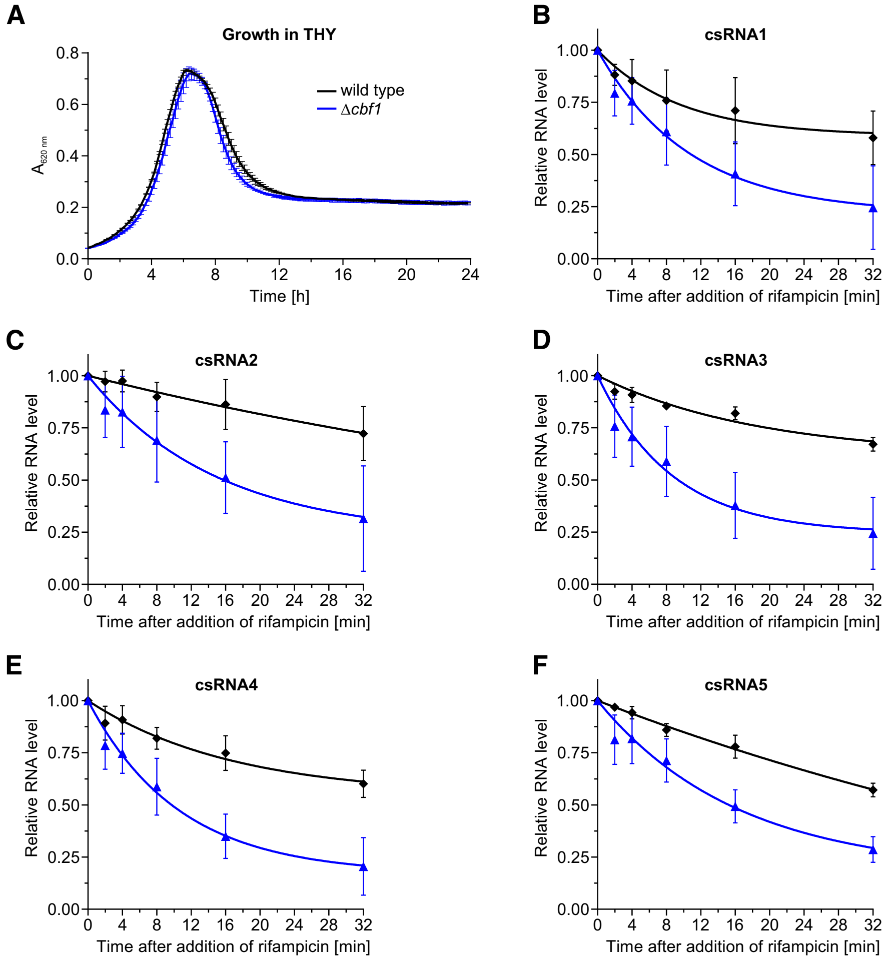

Figure EV5. Growth and csRNA stability in the absence of *cbf1*.

A Growth curves in rich medium. Deletion of *cbf1* has no influence on growth when compared to the wild type. The average cell density is based on three independent biological replicates.

B–F Determination of the stability of csRNAs based on rifampicin assays shown in Fig 6D. In the absence of *cbf1*, csRNAs become less stable when compared to the wild type. The relative RNA levels were calculated based on at least two independent experiments.

Data information: Error bars show SD from the mean.

 © 2020 The Authors
