# Supplementary figures and images for: Grad‐seq in a Gram‐positive bacterium reveals exonucleolytic sRNA activation in competence control
Source: EMBO J. 2020 Mar 30;39(9):e103852. doi: 10.15252/embj.2019103852 (PMC7196914; doi:10.15252/embj.2019103852)

Appendix FigS6A

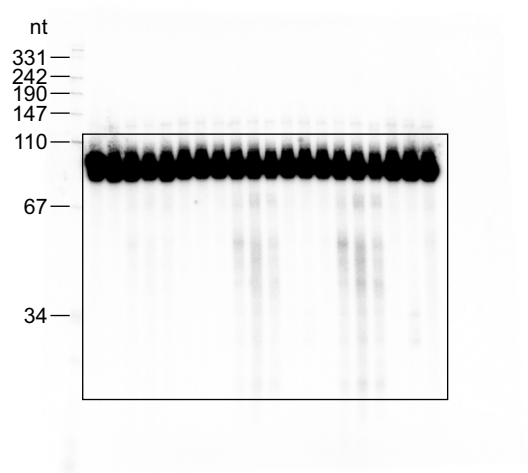

Appendix FigS6B

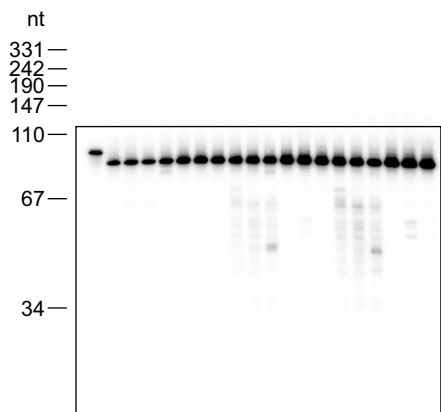

Appendix FigS6C

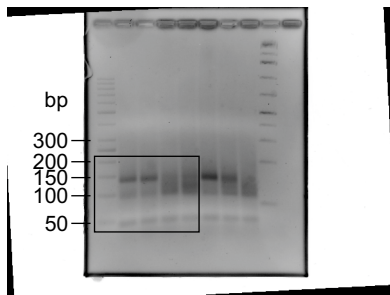

Supplement: Supplementary file 10 — Source Data for Appendix [file EMBJ-39-e103852-s016.zip › SourceDataForAppendixFigureS6A-C.pdf]

Appendix FigS7B

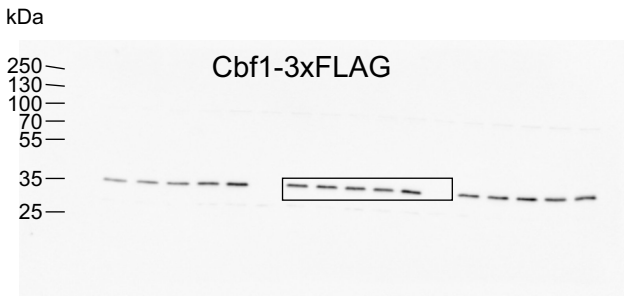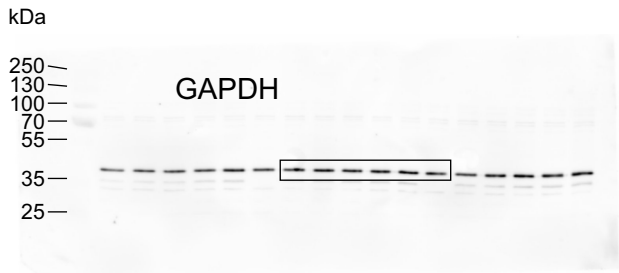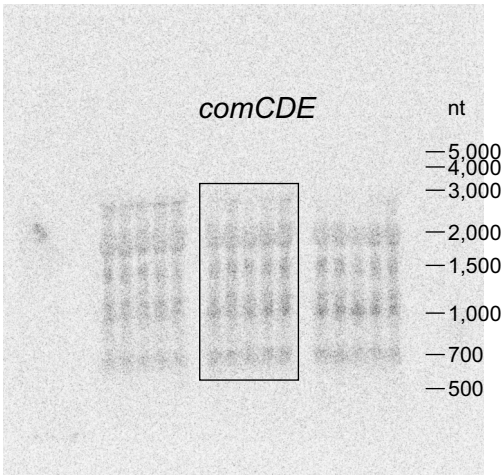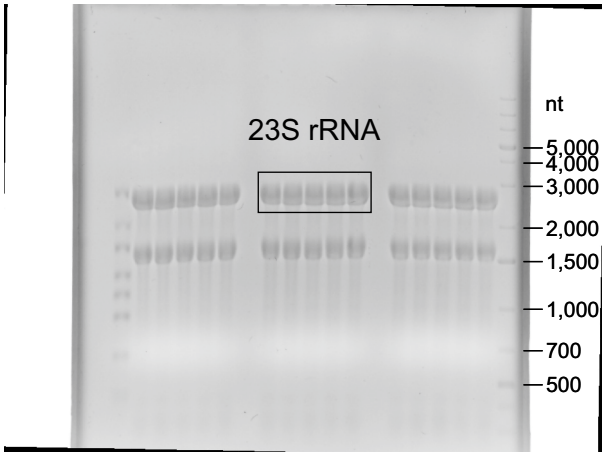

Supplement: Supplementary file 10 — Source Data for Appendix [file EMBJ-39-e103852-s016.zip › SourceDataForAppendixFigureS7B.pdf]

Fig1C

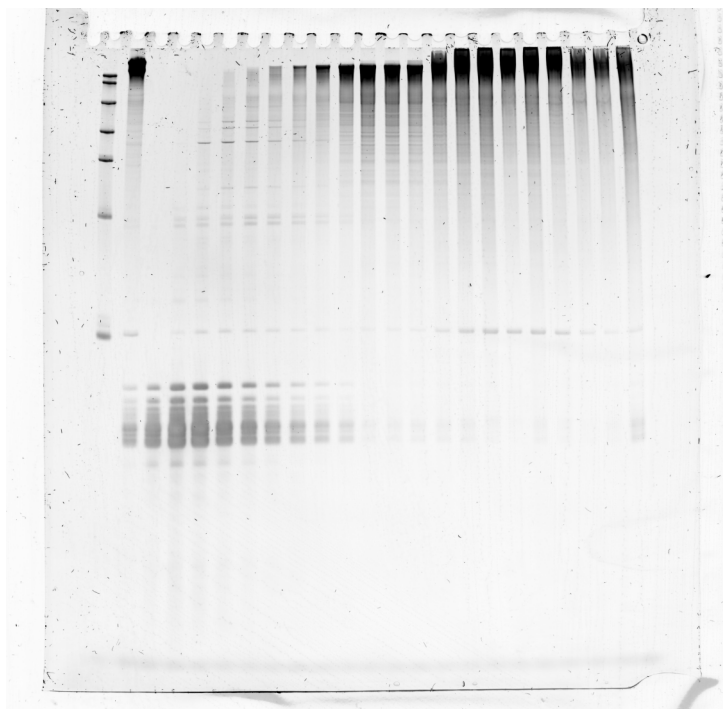

Fig1D

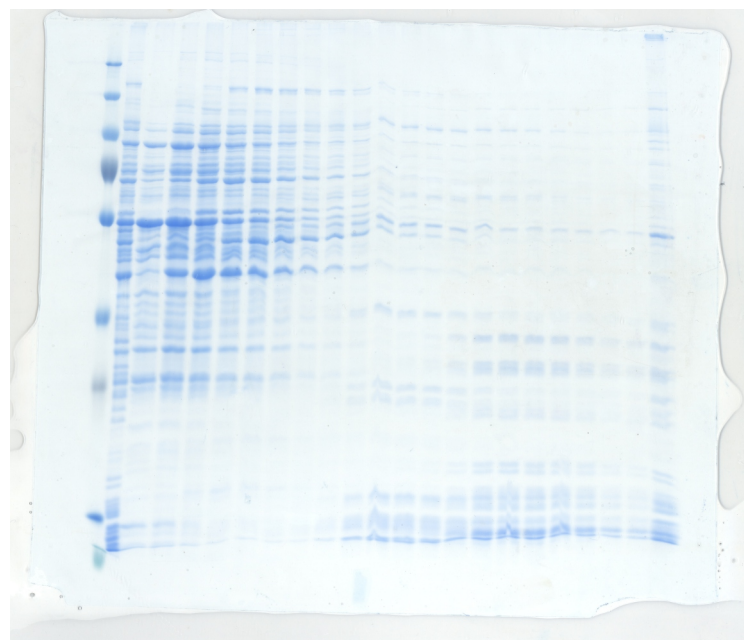

Supplement: Supplementary file 12 — Source Data for Figure 1 [file EMBJ-39-e103852-s010.pdf]

Fig3A

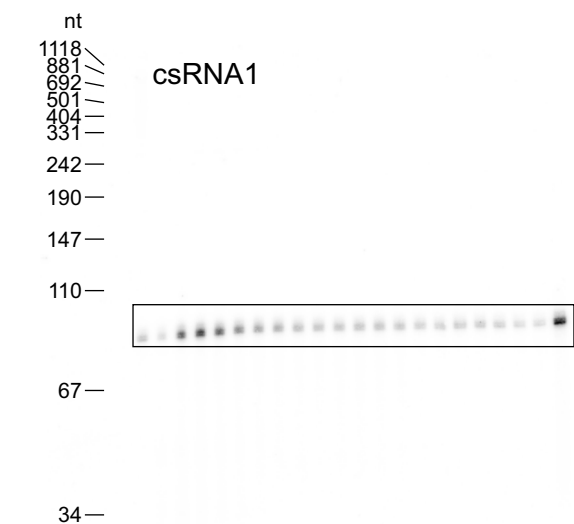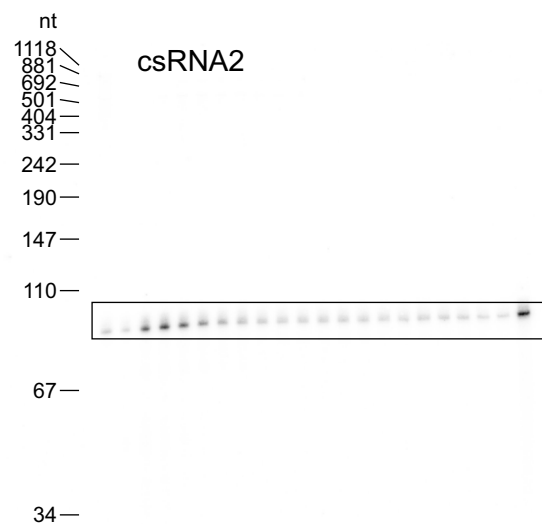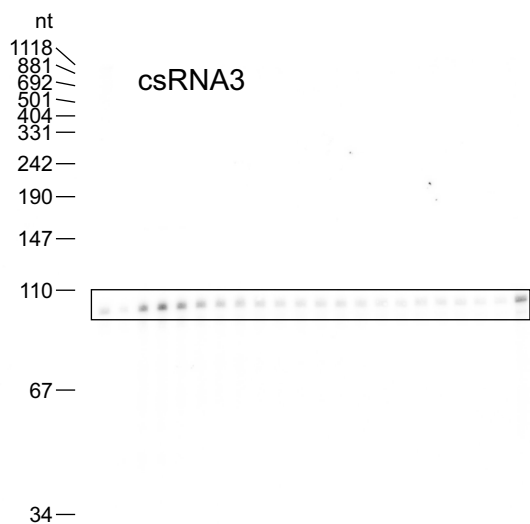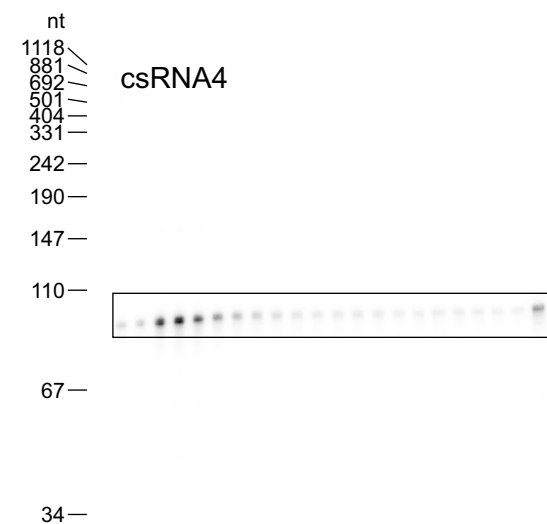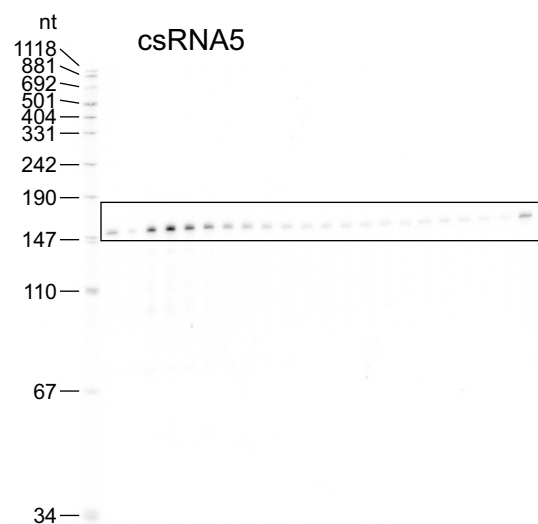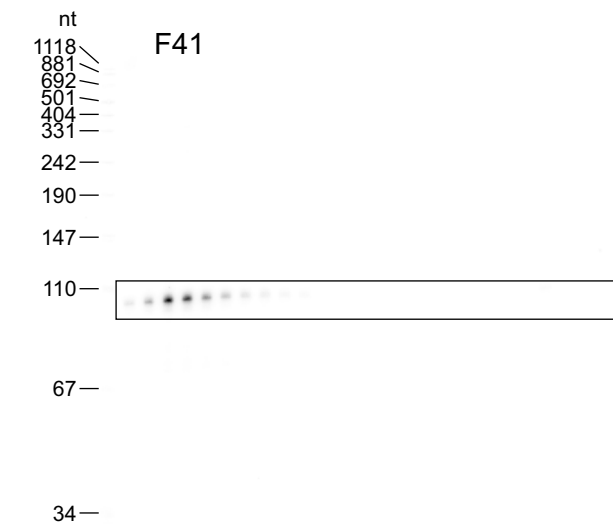

Fig3A (continued)

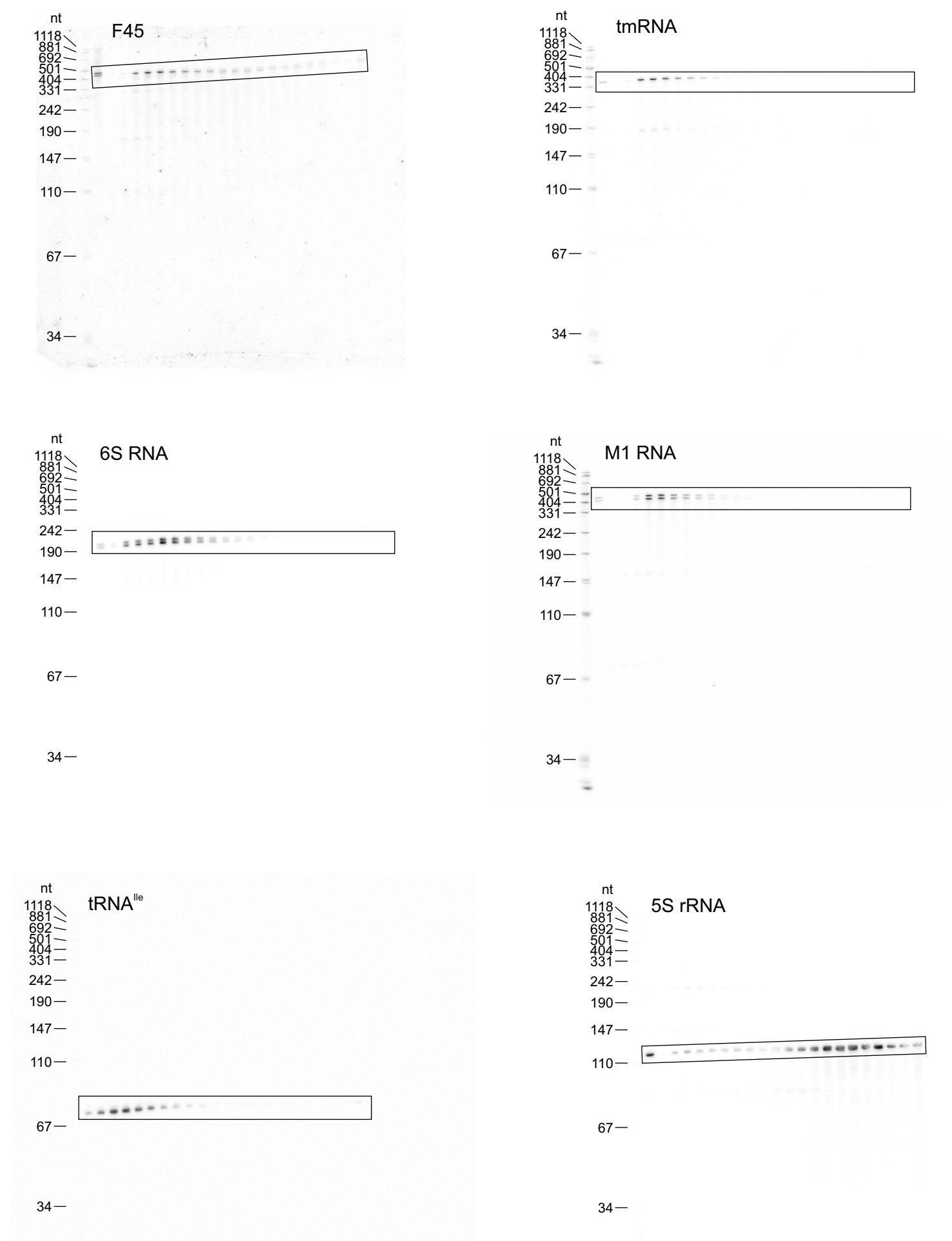

Supplement: Supplementary file 13 — Source Data for Figure 3 [file EMBJ-39-e103852-s011.pdf]

Fig4A

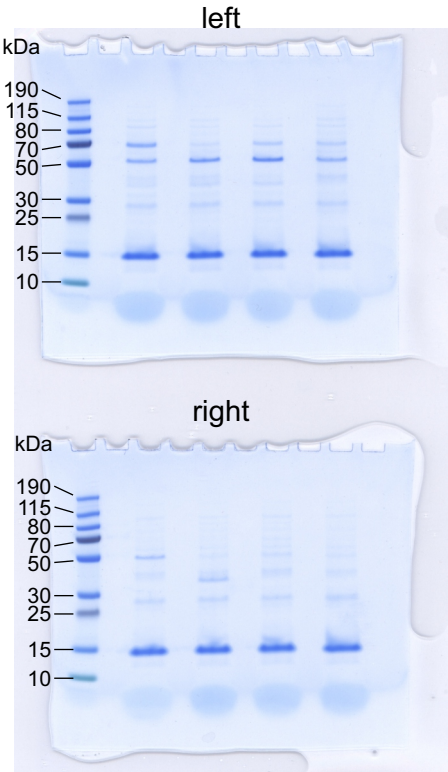

Supplement: Supplementary file 14 — Source Data for Figure 4 [file EMBJ-39-e103852-s012.pdf]

Fig5A

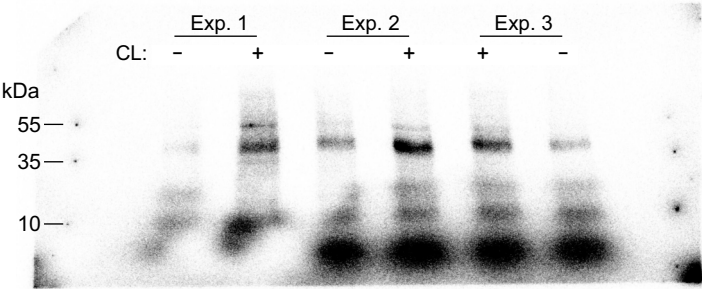

Supplement: Supplementary file 15 — Source Data for Figure 5 [file EMBJ-39-e103852-s013.pdf]

Fig6A

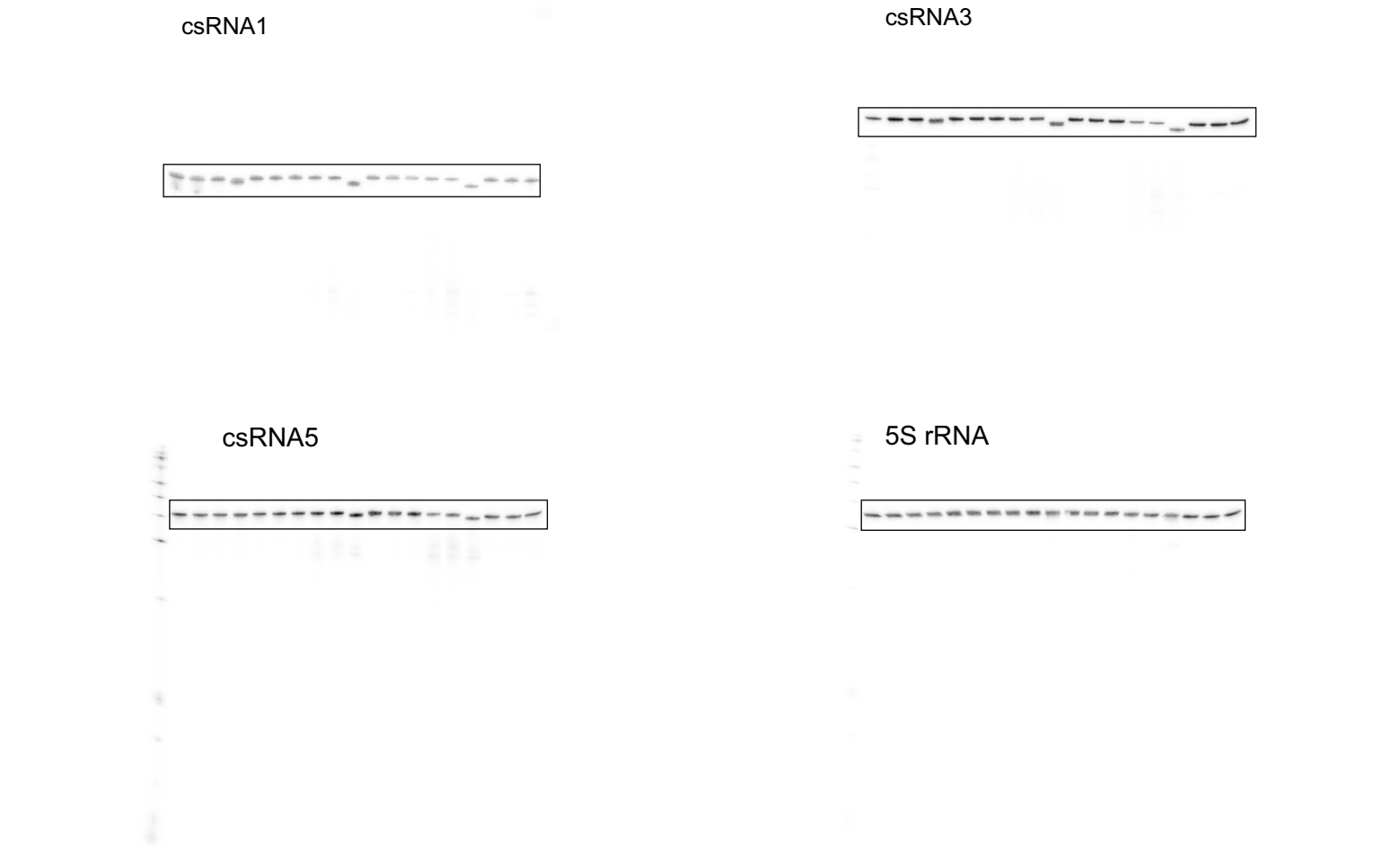

Fig6B

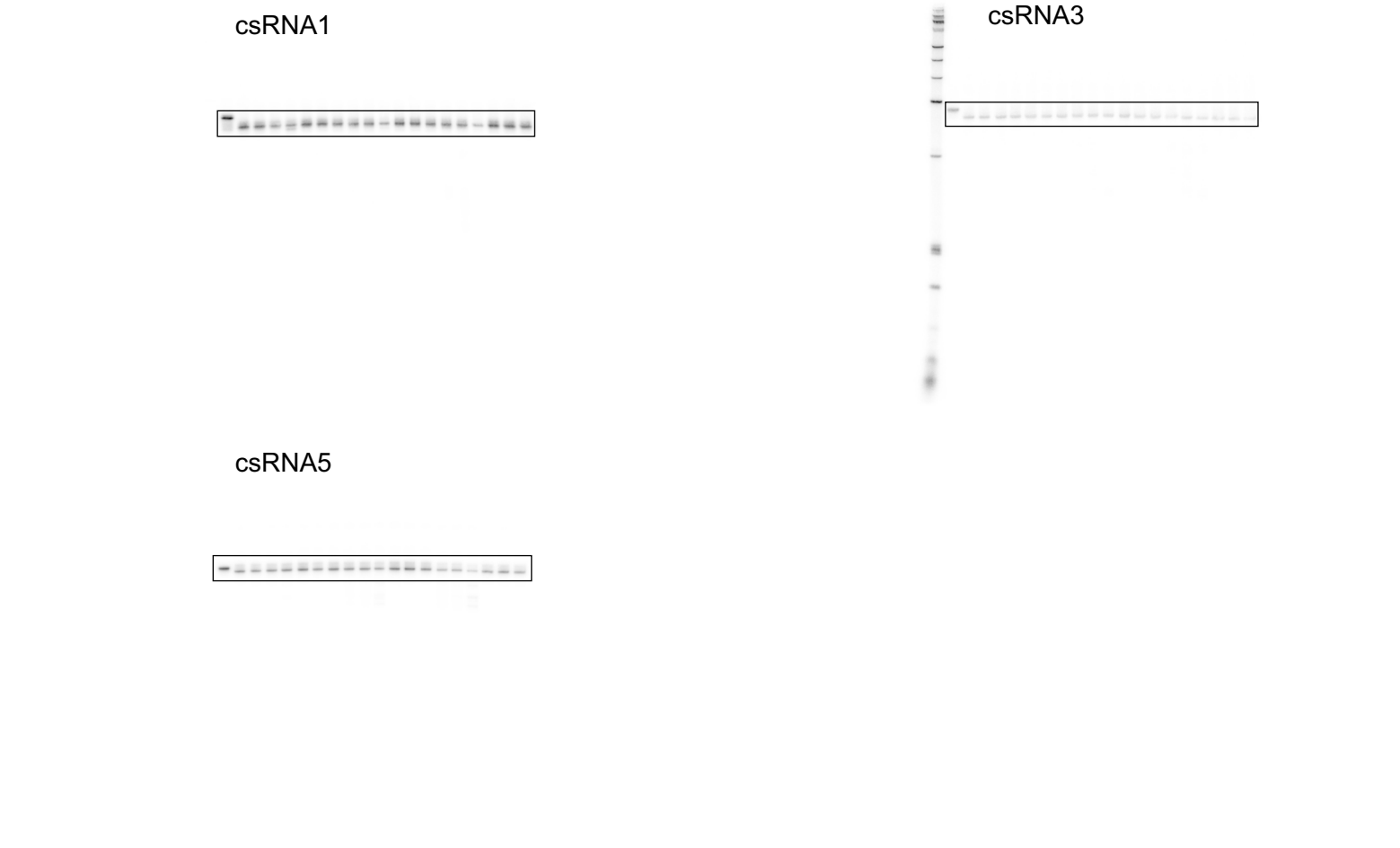

Fig6C

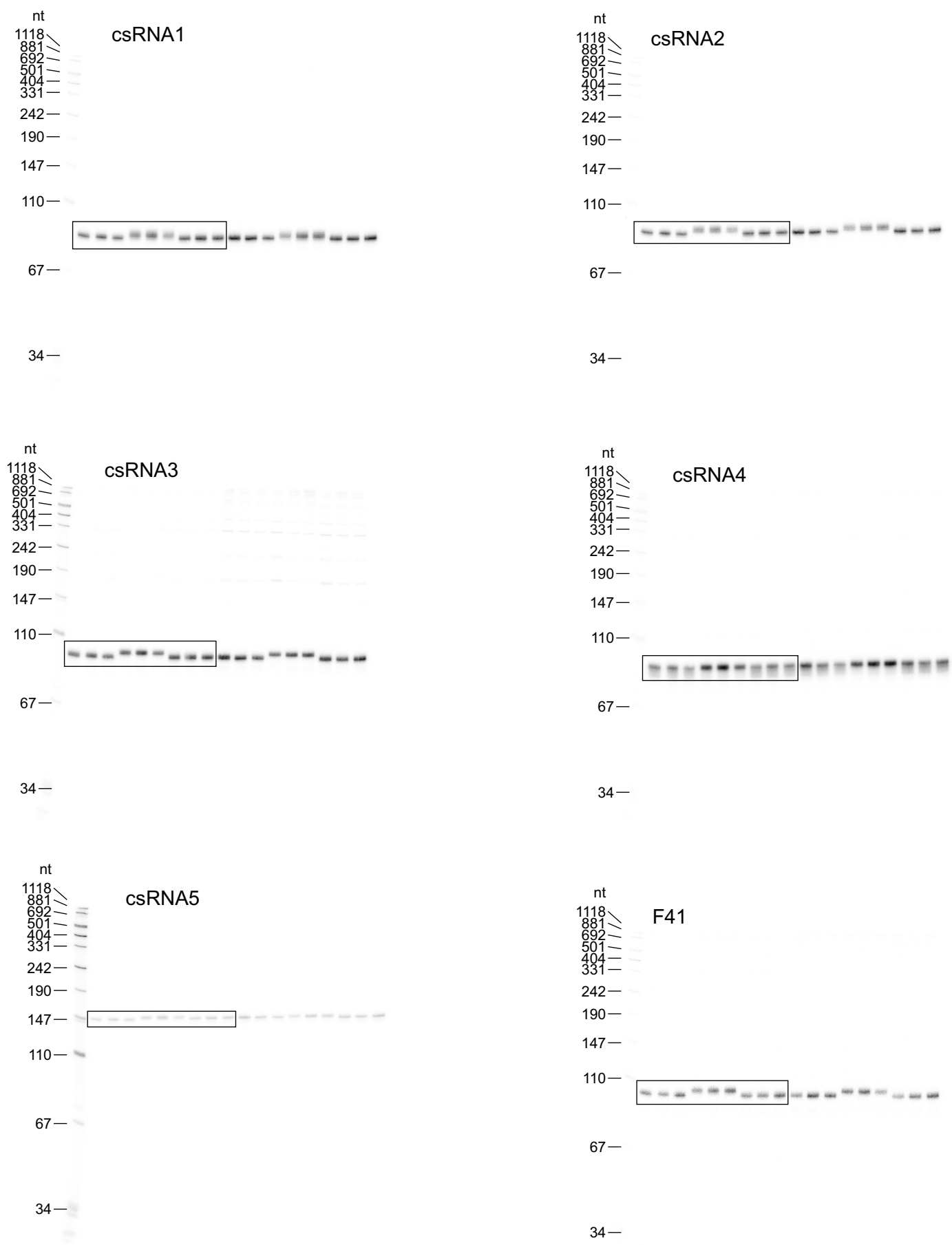

Fig6C (continued)

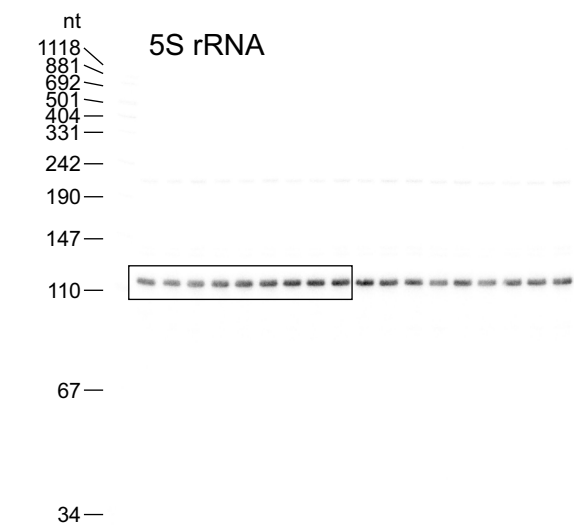

Fig6D

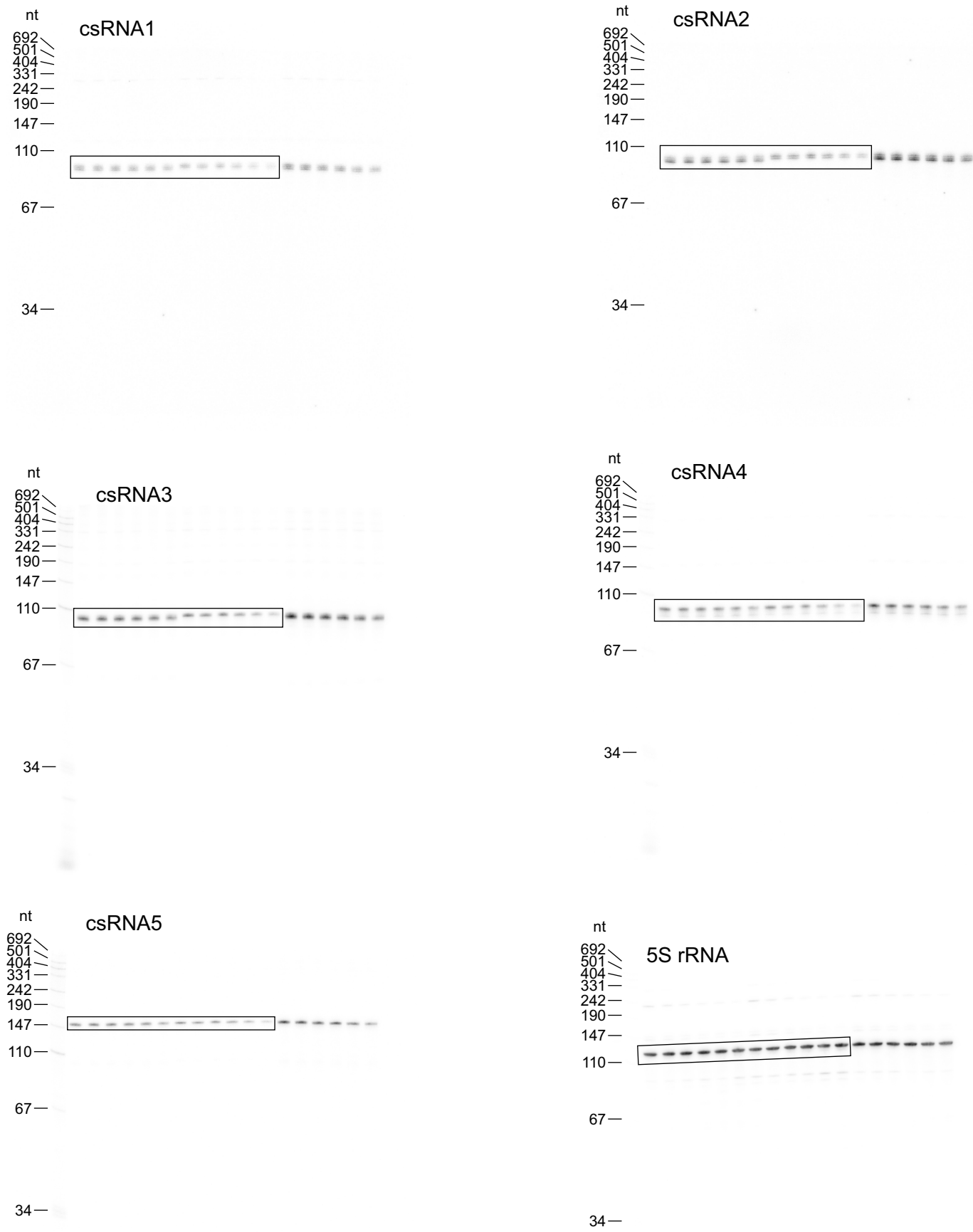

Supplement: Supplementary file 16 — Source Data for Figure 6 [file EMBJ-39-e103852-s014.pdf]

Fig7A

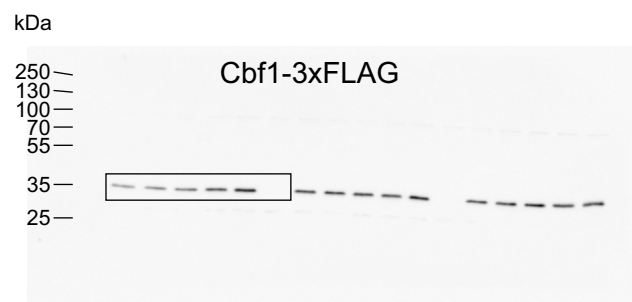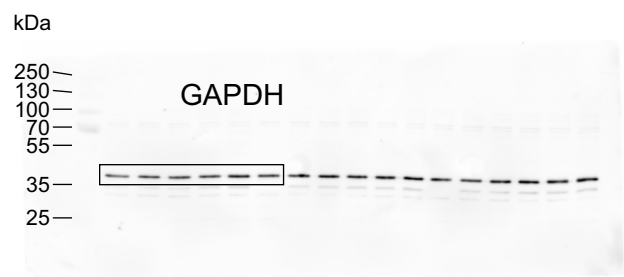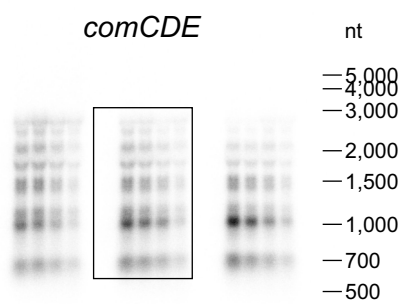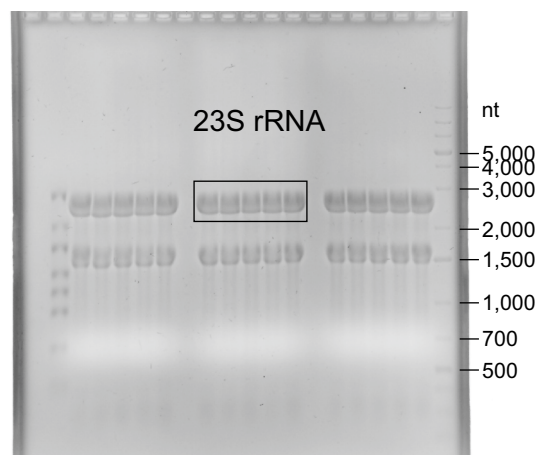

Supplement: Supplementary file 17 — Source Data for Figure 7 [file EMBJ-39-e103852-s015.pdf]
