# Supplementary material for: Grad‐seq in a Gram‐positive bacterium reveals exonucleolytic sRNA activation in competence control
Source: EMBO J. 2020 Mar 30;39(9):e103852. doi: 10.15252/embj.2019103852 (PMC7196914; doi:10.15252/embj.2019103852)
Supplement: Supplementary file 11 — Review Process File [file EMBJ-39-e103852-s017.pdf]

THE EMBO JOURNAL

Grad-seq in a Gram-positive bacteriumreveals exonucleolytic sRNA activation incompetence control

Jörg Vogel, Jens Hör, Genevieve Garriss, Silvia Di Giorgio, Lisa-Marie Hack, Jens Vanselow, Konrad Förstner, Andreas Schlosser and Birgitta Henriques-Normark

Review timeline:

Submission date:	28th October 2019
Editorial Decision:	27th November 2019
Revision received:	1st February 2020
Editorial Decision:	27th February
Revision received:	3rd March 2020
Accepted:	6th March 2020

Editor: Stefanie Boehm

Transaction Report:

(Note: With the exception of the correction of typographical or spelling errors that could be a source of ambiguity, letters and reports are not edited. The original formatting of letters and referee reports may not be reflected in this compilation.)

1st Editorial Decision 27th November 2019

Thank you for submitting your manuscript reporting Grad-seq of Streptococcus pneumoniae to The EMBO Journal. We have now received three referee reports on your study, which are included below for your information.

As you will see, the reviewers are overall positive and acknowledge the interest and quality of the study. Nonetheless they also raise several concerns that would need to be addressed in a revised manuscript. In particular, referee #1 and #3 find that it would be important to demonstrate that Cbf1 also stably interacts with csRNA in vivo. In addition, both referee #1 and #2 raise several more specific and technical issues that should be addressed, including providing additional controls and experimental details. Should you be able to adequately address the concerns each of the referees raises, then we would be happy to consider this study further for publication. Therefore I would like to invite you to prepare and submit a revised manuscript. Please note that it is our policy to allow only a single round of major revision and that it is therefore important to clarify all key concerns raised at this stage.

REFEREE REPORTS

Referee #1:

Bacterial sRNAs play an important role in regulating gene expression. This type of post-transcriptional regulation has been best characterized in Gram-negative bacteria. In these organisms sRNA-target interactions are frequently mediated by RNA-binding proteins such as Hfq and ProQ. However, our understanding of post-transcriptional regulation in Gram-positive bacteria is lagging far behind. In this manuscript the authors have employed Grad-seq to identify S. pneumoniae RNP complexes, focussing mostly on putative RNP complexes containing sRNAs. The authors present a

wealth of interesting data in the main text and accompanying supplementary tables, which should be a very useful resource for many groups working on Gram-positive bacteria. Additionally, they present convincing data demonstrating the robustness of the Grad-seq approach in identifying RNP complexes in Gram-positive bacteria. One of the key findings of their work was the discovery of S. pneumoniae RNP complexes containing sRNAs in association with the 3'-5' exonuclease Cbf1. Intuitively, one would think that recruitment of such nucleases would lead to destruction of the RNA, however, the authors show that Cbf1's enzymatic activity actually help to stabilize these sRNAs. The authors have done some really nice work demonstrating the enzymatic activity of Cbf1 in vitro and showed that deletion of Cbf1 decreases the half-lives of several sRNAs, consistent with the idea that Cbf1 is important for sRNA stability. Finally, the authors presented evidence that Cbf1 is involved in regulating competence in S. pneumoniae, highlighting the physiological relevance of their findings. Overall, it is a very interesting story, well written and the work is of a high standard.

Comments:

Page 4. The authors report the distribution of 141 sRNAs in the glycerol gradients and found that many form higher-order complexes. The sRNAs in many Gram-positive bacteria are poorly annotated and in some bacterial strains overlap with protein coding genes, which can complicate the interpretation of the sequencing data as it is not always clear if the signal originates from the sRNA or mRNA. How reliable is the sRNAs annotation in S. pneumoniae? Do the independently transcribed sRNAs show a similar distribution? Perhaps it would be useful to comment on this.

To confirm that the Cbf1 protein detected in fractions 3-5 indeed represents an RNP complex, it would be good to repeat the experiment using normal lysates and lysates treated with RNases, such as benzonase. Analysis of the fractions by Western (to detect Cbf1) and Northern blotting (to detect presence/absence of csRNAs) would suffice.

Page 5. The authors briefly mention their results obtained with the KhpA/B proteins. They state that it was detected in fractions 2-3 of the gradients. It would be helpful if the authors could add these results to Figure 1E, perhaps in the panel showing the GlyQ/S co-sedimentation with tRNA glycine. Or at least reference Figure S3 here.
I am not sure that based on the sedimentation behaviour of this heterodimer that the authors can conclude that these results "support" the proposed association with sRNAs and tRNAs, especially considering sRNAs sedimented throughout the gradient. Of course, such an association could easily be tested by performing an IP on these gradient fractions followed by Northern blot analyses. However, as it is not a main focus of the paper I would recommend rephrasing or removing this statement.

Page 6: "This yielded three distinct clusters .... (Fig. S4A). Should this not refer to Figure 2B or Figure S4B?

Page 6: "For unclear reasons, attempts to use the MS2 aptamer, which successfully captured RBPs from Salmonella lysates (Corcoran et al, 2012, Said et al, 2009, Smirnov et al, 2016)..." I would recommend also referencing the paper describing the MAPS approach developed by the Massé lab (Lalaouna and Massé, Mol Cell, 2015) and that they successfully applied it to E. coli. The Romby group has also shown it works in S. aureus.

Figure 3. Both the Grad-seq and the csRNA pull-down results suggest that the exonuclease Cbf1 could form a relatively stable complex with the (trimmed) csRNA transcripts. Presumably if the Cbf1 would simply rapidly process the csRNA and subsequently dissociate it would not have been found co-sedimenting with the bulk of the csRNAs? This begs the question whether stable binding of Cbf1 to its sRNA substrates after trimming is also important for controlling sRNA stability or function. Since the authors have recombinant Cbf1 protein and in vitro transcribed RNAs, perhaps they could do EMSAs to get an idea of binding constants and perform co-IP experiments with the FLAG-tagged Cbf1 followed by Northern blot analyses to confirm that it (stably) also interacts with these csRNAs in vivo. If from these experiments it can be concluded that stabilization/activation of the csRNAs not only involves 3' end trimming by Cbf1 but also stable binding of the exonuclease, then I think this would really strengthen the paper as I do not believe such a dual role for an exonuclease has been described before.

© European Molecular Biology Organization

Have the authors tried CLIP or RIP-seq with the Cbf1-FLAG strain? I will obviously not ask them to do this but it would be interesting to know if these csRNAs are the main targets of Cbf1.

Page 6: "of these were also much more abundant in the pellet than in the gradient fractions (Fig. S4D)". Perhaps also refer here to Figure 2A as it is also very clear from the Northern blots.

Figure 4D. It would be useful to quantify the Northern blot data in Figure 4D to make the data easier to interpret. Furthermore, the shift of the csRNAs observed in the knock-out strain is not very clear in this image. Presumably this is the result of different gel percentage? Perhaps it would be useful to comment on this in the legend of Figure 4D.

Figure 5A and S6A. Why is there such a noticeable difference between the biological replicates showing the changes in Cbf1-3xFLAG protein levels upon addition of CSP? The results in Fig. S6A do not look very convincing to me. I am also not sure if Ponceau S staining is an accurate way to quantify total protein loading in each well. Considering the changes in protein levels upon CSP-stimulation are between 1.2- to 2-fold, I think this experiment deserves more robust quantification of the data. Perhaps the authors could probe these membranes with antibodies recognizing an abundant chaperone and use that as a loading control. What are the additional bands in Figure S6A and are the samples next to it replicates of the same experiment? If the increase in Cbf1-3xFLAG is based on three biological replicates, perhaps the authors could show all three and produce a bar plot similar to Fig. S6B?

Page 9: "While the S. pneumoniae strain R6 spontaneously develops competence when grown at pH 7.9-8.0"...
Perhaps start this sentence with Because?

Page 13:"Arguably, our model does not exclude possible csRNA-independent functions of Cbf1 in competence regulation, which remain to be tested by combined inactivation of cbf1 and multiple csRNAs". I appreciate that the authors pointed this out. Presumably now that they have a FLAG-tagged Cbf1 they will try to do CLIP to test binding to other targets? It would be interesting to know if Cbf1 has other substrates.

Although I think the data in Figure 6 and associated Supplementary figures is very interesting, it is a bit descriptive and interrupts the flow of the paper. I would recommend making this Figure 2 and the text could be placed before the section describing the identification of biochemically distinct classes of ncRNAs.

Figure 6. Very minor point, but it would be useful to indicate in which fractions the 30S and 50S subunits were detected.

Discussion section

As the authors implied, the observation that the activity of an exonuclease stabilizes a non-coding RNA is intriguing and perhaps somewhat counterintuitive. However, the idea that exonucleases help to stabilize non-coding RNAs is not new as there are a number of papers in the eukaryotic RNA field that have shown that proper 3' end processing of ncRNAs, such as small nucleolar RNAs, is not only important for their maturation but also for their stability and therefore their function. Perhaps the authors can comment on any parallels with data from eukaryotic model organisms?

Referee #2:

Review of the manuscript « Grad-seq in a Gram-positive bacterium reveals exonucleolytic sRNA activation in competence control" by Hör et al.

In this work, the authors are describing the use of a previously developed technique, called Grad-seq, allowing the investigation of RNA and protein complexes in the bacterial pathogen Streptococcus pneumoniae. By using Grad-seq, they were able to demonstrate the function of the exonuclease Cbf1 in stabilization of sRNAs involved in the repression of competence. This is a powerful technique that can also help defining which cellular proteins, such as metabolic complexes

or secretion systems, are in complex in vivo. The findings described here are interesting and put the focus on Cbf1 in the regulation of an important bacterial function.

Major comments:
There is no description nor reference of the t-SNE analysis performed for Figure 2B. Please add details in Results and Material and Methods sections since this is quite central to the downstream experiments described in the manuscript.

In the Corcoran, 2012 and Said, 2009 papers, the MS2-tagged sRNAs were expressed in vivo and succeeded to co-purify in vivo assembled RNA-proteins complexes, which contrasts to the attempt described in the 2nd paragraph of page 7. The authors should discuss this discrepancy.

In Fig 4B, as noted by the authors the csRNA1 seems to be sensitive to Cbf1 cleavage in the presence of MnCl2: there is a clear lower band at time "0" and less RNA is visible after 5 and 15 min incubation. I suggest performing a 3'RACE experiment to determine the exact position at which the 3'-5' exo stops trimming. This would confirm the previous observation that secondary structures block the progression of Cbf1. Moreover, the description of in vitro stoichiometry of RNA and protein in the figure legend or methods section is necessary to appreciate the efficacy of the experiment.

Fig 4D uses a Δcbf1 mutant without any description of cells growth. A growth curve of the mutant compared to WT background should be added.

While the half-life is clearly reduced by the absence of Cbf1, the steady-state levels of csRNAs at time "0" do not seem to be significantly affected by the absence of Cbf1 in Fig 4B. This observation is contrasting with the proposed mechanism in Fig 5C where Cbf1 regulation of csRNAs by cleavage leads to ComC modulation.

Ideally, I would add a positive control related to competence in Fig 5A. For instance, Northern blots on comCDE or comAB, both of which should increase under conditions of CSP expression.

Fig 5B is not the most convincing with overlapping error bars. I would add a Northern blot showing the expression of comC mRNA or a Western showing ComC (CSP) protein. Both comC mRNA and ComC protein are expected to increase in conditions of absence of cbf1 product. This would help demonstrate the activation of the competence regulon.

The section described in Fig 6 could probably be moved to right after Fig 3 when the manuscript is still demonstrating the general interest of using Grad-seq. Thereafter, the manuscript would start focusing on the cell's competence, Cbf1 and csRNAs, instead of going back to the general usefulness of the Grad-seq technique.

Minor comments:
Please define csRNAs as there is no background on these specific sRNAs in the text.

Fig 5C shows ComC protein losing an unidentified portion before being transported outside the cell. This should be defined better.

Referee #3:

Previously, the authors' group developed gradient profiling by sequencing (Grad-seq) to analyze functional RNAs and RNA binding proteins in bacterial cells. This paper reports a successful application of this method to the study of sRNAs and their partner proteins in Gram-positive in Streptococcus pneumoniae, focusing on csRNAs that are post-transcriptional negative regulators of comC mRNA encoding the precursor protein of the competence-stimulating peptide (CSP). Through the Grad-seq analysis and tag-based pull-down assay, the authors identified the interactions between csRNAs and an exoribonuclease Cbf1. Then, they showed evidence that Cbf1 trims the 3' ends of csRNAs resulting in the stabilization of these RNAs. Furthermore, the authors showed that

the transformation ability of the S. pneumoniae cells significantly increases when the cbf1 gene is disrupted. Taken together, they propose a model in which Cbf1-mediated stabilization of csRNAs contributes to a negative feedback loop that controls competence after CSP stimulation.
The data seem to be convincing to support the conclusion and the paper is well written. I have no serious criticisms on this manuscript. My only comment is that the work/conclusion would be strengthened if the authors are able to pull-down csRNAs using tagged-Cbf1 protein as a bait.

© European Molecular Biology Organization

Re: EMBOJ-2019-103852
Grad-seq in a Gram-positive bacterium reveals exonucleolytic sRNA activation in competence control

Scope of revisions defined by the editor:

As you will see, the reviewers are overall positive and acknowledge the interest and quality of the study. Nonetheless they also raise several concerns that would need to be addressed in a revised manuscript. In particular, referee #1 and #3 find that it would be important to demonstrate that Cbf1 also stably interacts with csRNA in vivo. In addition, both referee #1 and #2 raise several more specific and technical issues that should be addressed, including providing additional controls and experimental details.

We would like to thank the reviewers for their insightful and constructive comments on our manuscript. We'll start with an overview of our changes to the manuscript and a detailed point-by-point discussion will follow below:

Overview of revisions:

- All reviewers asked for more evidence regarding the *in vivo* interaction of Cbf1 and ncRNAs. To address this, we have performed the requested UV CLIP-seq experiment to analyze the *in vivo* targetome of Cbf1. The results clearly bolster our previous findings, by identifying the csRNAs amongst the top-enriched transcripts and thus adding independent proof for their recognition by Cbf1 *in vivo*. These results are presented in a new Figure 5 and Dataset EV5.

- Two of the reviewers explicitly state that our study provides an important resource to the *S. pneumoniae* field and that it would be good to put more emphasis on the predicted protein-protein interactions as well. As suggested, we have re-organized the manuscript and moved Figure 6 and the accompanying paragraphs more to the front (now Figure 2).

- We have performed additional experiments, including 3'RACE, to get more insight into how RNA secondary structure blocks the progression of the Cbf1 3'-to-5' exonuclease.

- Lastly, based on the reviewers' comments, we performed or added (where already available) several requested control experiments.

Point-by-point reply:

Referee #1:

Bacterial sRNAs play an important role in regulating gene expression. This type of post-transcriptional regulation has been best characterized in Gram-negative bacteria. In these organisms sRNA-target interactions are frequently mediated by RNA-binding proteins such as Hfq and ProQ. However, our understanding of post-transcriptional regulation in Gram-positive bacteria is lagging far behind. In this manuscript the authors have employed Grad-seq to identify S. pneumoniae RNP complexes, focussing mostly on putative RNP complexes containing sRNAs. The authors present a wealth of interesting data in the main text and accompanying supplementary tables, which should be a very useful resource for many groups working on Gram-positive bacteria. Additionally, they

present convincing data demonstrating the robustness of the Grad-seq approach in identifying RNP complexes in Gram-positive bacteria. One of the key findings of their work was the discovery of S. pneumoniae RNP complexes containing sRNAs in association with the 3'-5' exonuclease Cbf1. Intuitively, one would think that recruitment of such nucleases would lead to destruction of the RNA, however, the authors show that Cbf1's enzymatic activity actually help to stabilize these sRNAs. The authors have done some really nice work demonstrating the enzymatic activity of Cbf1 in vitro and showed that deletion of Cbf1 decreases the half-lives of several sRNAs, consistent with the idea that Cbf1 is important for sRNA stability. Finally, the authors presented evidence that Cbf1 is involved in regulating competence in S. pneumoniae, highlighting the physiological relevance of their findings. Overall, it is a very interesting story, well written and the work is of a high standard.

Comments:

Page 4. The authors report the distribution of 141 sRNAs in the glycerol gradients and found that many form higher-order complexes. The sRNAs in many Gram-positive bacteria are poorly annotated and in some bacterial strains overlap with protein coding genes, which can complicate the interpretation of the sequencing data as it is not always clear if the signal originates from the sRNA or mRNA. How reliable is the sRNAs annotation in S. pneumoniae? Do the independently transcribed sRNAs show a similar distribution? Perhaps it would be useful to comment on this.

REPLY: Thank you for highlighting this important issue. For our biocomputational analysis, we used the annotation from a recently published study in *S. pneumoniae* strain TIGR4 (Warrier et al. 2018 PLoS Pathogens). This annotation does indeed contain different classes of ncRNAs such as riboswitches or sRNAs. We did not want to delete any entries because this would have complicated cross-comparison by others with past publications of ncRNA work in the TIGR4 strain. While we already strove to discriminate between ncRNAs and sRNAs in the original manuscript, have now added further statements of clarification (on pages 5 and 8). Moreover, we have added a figure showing an overview of the distribution of sRNAs of which we are confident that they are primary transcripts (Fig EV1C). This classification is based on our unpublished differential RNA-seq data for the identification of transcription start sites in *S. pneumoniae* TIGR4. Importantly, the distribution of these RNAs is very similar to the distribution found for cluster 3 of our global analysis (Fig EV4F). Conversely, cluster 3 contains the majority of the primary sRNAs shown in Fig EV1C.

To confirm that the Cbf1 protein detected in fractions 3-5 indeed represents an RNP complex, it would be good to repeat the experiment using normal lysates and lysates treated with RNases, such as benzonase. Analysis of the fractions by Western (to detect Cbf1) and Northern blotting (to detect presence/absence of csRNAs) would suffice.

REPLY: More evidence for an *in vivo* interaction between csRNAs and Cbf1 was requested by all reviewers, and there are several ways to approach this. We followed the suggestion by this reviewer (see below) and reviewer #3, and performed UV CLIP-seq, because this method also identifies binding regions in the RNA. The UV CLIP-seq experiment were added as a new Figure 5, with accompanying text on pages 9-10. Importantly, the sRNAs F41, csRNA1, csRNA3 and csRNA5 were amongst the top enriched transcripts. The CLIP-seq data further show that binding of Cbf1 takes place at the 3' ends of the RNAs, as expected (Figure 5D).

The above-suggested gradients with/without RNase treatment are certainly an exciting approach. As it happens, we are putting the finishing touches to a manuscript describing how we

have used this type of experiment to search globally for new RBPs, and we would like to keep the novelty for that story.

Page 5. The authors briefly mention their results obtained with the KhpA/B proteins. They state that it was detected in fractions 2-3 of the gradients. It would be helpful if the authors could add these results to Figure 1E, perhaps in the panel showing the GlyQ/S co-sedimentation with tRNA glycine. Or at least reference Figure S3 here.
I am not sure that based on the sedimentation behaviour of this heterodimer that the authors can conclude that these results "support" the proposed association with sRNAs and tRNAs, especially considering sRNAs sedimented throughout the gradient. Of course, such an association could easily be tested by performing an IP on these gradient fractions followed by Northern blot analyses. However, as it is not a main focus of the paper I would recommend rephrasing or removing this statement.

REPLY: We agree that KhpA/B is a very interesting RBP complex. It is now in a main figure, together with other RBPs in Figure 2B. In addition, we have re-phrased the sentence about possible interactions (page 7).

Page 6: "This yielded three distinct clusters .... (Fig. S4A). Should this not refer to Figure 2B or Figure S4B?

REPLY: We thank the reviewer for spotting this mistake. It's been corrected to Figure EV4B.

Page 6: "For unclear reasons, attempts to use the MS2 aptamer, which successfully captured RBPs from Salmonella lysates (Corcoran et al, 2012, Said et al, 2009, Smirnov et al, 2016)..." I would recommend also referencing the paper describing the MAPS approach developed by the Massé lab (Lalaouna and Massé, Mol Cell, 2015) and that they successfully applied it to E. coli. The Romby group has also shown it works in S. aureus.

REPLY: We agree that mention of these additional papers is warranted. We have added them on page 9.

Figure 3. Both the Grad-seq and the csRNA pull-down results suggest that the exonuclease Cbf1 could form a relatively stable complex with the (trimmed) csRNA transcripts. Presumably if the Cbf1 would simply rapidly process the csRNA and subsequently dissociate it would not have been found co-sedimenting with the bulk of the csRNAs? This begs the question whether stable binding of Cbf1 to its sRNA substrates after trimming is also important for controlling sRNA stability or function. Since the authors have recombinant Cbf1 protein and in vitro transcribed RNAs, perhaps they could do EMSAs to get an idea of binding constants and perform co-IP experiments with the FLAG-tagged Cbf1 followed by Northern blot analyses to confirm that it (stably) also interacts with these csRNAs in vivo. If from these experiments it can be concluded that stabilization/activation of the csRNAs not only involves 3' end trimming by Cbf1 but also stable binding of the exonuclease, then I think this would really strengthen the paper as I do not believe such a dual role for an exonuclease has been described before.

REPLY: As described further above, we have opted to perform UV CLIP-seq in order to add further support for an *in vivo* interaction of Cbf1 and the csRNAs.

Have the authors tried CLIP or RIP-seq with the Cbf1-FLAG strain? I will obviously not ask them to do this but it would be interesting to know if these csRNAs are the main targets of Cbf1.

REPLY: See above. Our CLIP-seq data indeed show that the csRNAs are amongst the main ncRNA targets of Cbf1.

Page 6: "of these were also much more abundant in the pellet than in the gradient fractions (Fig. S4D)". Perhaps also refer here to Figure 2A as it is also very clear from the Northern blots.

REPLY: Perhaps the reviewer means cluster 3 instead of 2? The sentence in question refers to cluster 2, whereas Figure 2A (now Figure 3A) mostly shows ncRNAs included in cluster 3.

Figure 4D. It would be useful to quantify the Northern blot data in Figure 4D to make the data easier to interpret. Furthermore, the shift of the csRNAs observed in the knock-out strain is not very clear in this image. Presumably this is the result of different gel percentage? Perhaps it would be useful to comment on this in the legend of Figure 4D.

REPLY: This is an excellent observation. The shift in the gel is less visible in previous Figure 4D as it is in 4C (now Figure 6C/D) because 6% PAA instead of 10% PAA was used in this gel. We want to point out, however, that the shift is still visible in this gel. We have amended the figure legend and added plots showing the decay based on rifampicin assays to Figure EV5B-F.

Figure 5A and S6A. Why is there such a noticeable difference between the biological replicates showing the changes in Cbf1-3xFLAG protein levels upon addition of CSP? The results in Fig. S6A do not look very convincing to me. I am also not sure if Ponceau S staining is an accurate way to quantify total protein loading in each well. Considering the changes in protein levels upon CSP-stimulation are between 1.2- to 2-fold, I think this experiment deserves more robust quantification of the data. Perhaps the authors could probe these membranes with antibodies recognizing an abundant chaperone and use that as a loading control. What are the additional bands in Figure S6A and are the samples next to it replicates of the same experiment? If the increase in Cbf1-3xFLAG is based on three biological replicates, perhaps the authors could show all three and produce a bar plot similar to Fig. S6B?

REPLY: We thank the reviewer for pointing this out, we should have clarified this in the first place. Figure 5A (now Figure 7A) shows results with CSP induction. Figure S6A (now Appendix Figure S7B) is not a biological replicate, it is the same experiment without CSP induction, in order to exclude the possibility that the observed upregulation of Cbf1 is caused by continued cell growth. We have clarified this on page 12.

In addressing a request by reviewer #2, we have repeated the experiment and in doing so included GAPDH as a loading control. In addition, we have included a northern blot showing *comCDE* transcript levels as a positive control and added the suggested bar chart to Appendix Figure S7A.

The additional bands observed in the uncropped image are unspecific bands of the FLAG antibody that typically appear when we reuse the antibody (which we routinely do in our lab).

Page 9: "While the S. pneumoniae strain R6 spontaneously develops competence when grown at pH 7.9-8.0"...
Perhaps start this sentence with Because?

REPLY: We re-phrased the sentence to clarify the intended meaning (page 12).

Page 13:"Arguably, our model does not exclude possible csRNA-independent functions of Cbf1 in competence regulation, which remain to be tested by combined inactivation of cbf1 and multiple csRNAs". I appreciate that the authors pointed this out. Presumably now that they have a FLAG-tagged Cbf1 they will try to do CLIP to test binding to other targets? It would be interesting to know if Cbf1 has other substrates.

REPLY: As described further above, we performed and included UV CLIP-seq data. Yes, it does appear that Cbf1 has other substrates (especially mRNAs), and we hope that the data provided here will inspire others to follow up on some of these additional candidates.

Although I think the data in Figure 6 and associated Supplementary figures is very interesting, it is a bit descriptive and interrupts the flow of the paper. I would recommend making this Figure 2 and the text could be placed before the section describing the identification of biochemically distinct classes of ncRNAs.

REPLY: Reviewer #2 had similar advice, so we have decided to rearrange this part of the manuscript, moving the protein complexes up and Figure 6 (now Figure 2) more to the front.

Figure 6. Very minor point, but it would be useful to indicate in which fractions the 30S and 50S subunits were detected.

REPLY: We have added small pointers to the 30S and 50S subunits in what are now Figures 2 and EV3.

Discussion section

As the authors implied, the observation that the activity of an exonuclease stabilizes a non-coding RNA is intriguing and perhaps somewhat counterintuitive. However, the idea that exonucleases help to stabilize non-coding RNAs is not new as there are a number of papers in the eukaryotic RNA field that have shown that proper 3' end processing of ncRNAs, such as small nucleolar RNAs, is not only important for their maturation but also for their stability and therefore their function. Perhaps the authors can comment on any parallels with data from eukaryotic model organisms?

REPLY: Excellent point. Our previous discussion was focused on bacteria, hence limited to the similarities to PNPase in Gram-negative organisms. We now also mention eukaryotic microRNA, piRNA, siRNA as well as snoRNA processing (page 14-15).

Referee #2:

Review of the manuscript « Grad-seq in a Gram-positive bacterium reveals exonucleolytic sRNA activation in competence control" by Hör et al.

In this work, the authors are describing the use of a previously developed technique, called Grad-seq, allowing the investigation of RNA and protein complexes in the bacterial pathogen Streptococcus pneumoniae. By using Grad-seq, they were able to demonstrate the function of the exonuclease Cbf1 in stabilization of sRNAs involved in the repression of competence. This is a powerful technique that can also help defining which cellular proteins, such as metabolic complexes or secretion systems, are in complex in vivo. The findings described here are interesting and put the focus on Cbf1 in the regulation of an important bacterial function.

Major comments:
There is no description nor reference of the t-SNE analysis performed for Figure 2B. Please add details in Results and Material and Methods sections since this is quite central to the downstream experiments described in the manuscript.

REPLY: This is indeed important information; we have added these details to the M&M section. While revising this section, we have also amended the t-SNE plot (Figure 3B in the revised manuscript). The previous version included information from the k-means clustering (that we used to obtain the cluster numbers for coloring) in order to obtain a sharper separation. However, we think that for better comparison with, for example, the previous *Salmonella* Grad-seq maps (Smirnov et al., PNAS, 2016), it will be better to show the t-SNE plot without this extra information. The original version of the t-SNE is now shown in Appendix Figure S4 and the M&M section explains the difference between these two types of presentation.

In the Corcoran, 2012 and Said, 2009 papers, the MS2-tagged sRNAs were expressed in vivo and succeeded to co-purify in vivo assembled RNA-proteins complexes, which contrasts to the attempt described in the 2nd paragraph of page 7. The authors should discuss this discrepancy.

REPLY: It is true that in these earlier two mentioned papers we used plasmid-based expression of the tagged RNAs. However, we subsequently switched to an *in vitro* approach (Smirnov et al. 2016 PNAS) for higher throughput. Both variants have been successful in recovering RBPs from cell lysates. In the present study, we decided to take the more streamlined *in vitro* approach, which in the mentioned PNAS paper successfully identified ProQ as a global RBP. We have clarified this on page 9.

In Fig 4B, as noted by the authors the csRNA1 seems to be sensitive to Cbf1 cleavage in the presence of $MnCl_2$: there is a clear lower band at time "0" and less RNA is visible after 5 and 15 min incubation. I suggest performing a 3'RACE experiment to determine the exact position at which the 3'-5' exo stops trimming. This would confirm the previous observation that secondary structures block the progression of Cbf1. Moreover, the description of in vitro stoichiometry of RNA and protein in the figure legend or methods section is necessary to appreciate the efficacy of the experiment.

REPLY: We agree that the observation that truncated csRNA1 can get reduced in total quantity by Cbf1 is interesting. As already suggested in the main text, a Cbf1-mediated removal of the 6 Us (see panel B of what is now Figure 6) could lead to a less stable 3’ structure. Indeed, based on *in silico* structure predictions, 3 of the 6 Us are part of the terminator stem and pair with A or G, indicating that there might be structural breezing in the truncated RNA, which would then allow for digestion by Cbf1. YhaM, the *B. subtilis* homolog of Cbf1, was shown to be able to completely degrade unstructured regions of RNA until it reaches a stem loop structure (Oussenko et al. 2002 J Bacteriol). Using a fully linear RNA substrate, YhaM was even able to completely digest the whole RNA.

The probe used in our initial northern blot detection of csRNA1 binds in the 3’ region (which is the only region that significantly differs from csRNA2), so we might have missed shorter digestion products where Cbf1 stopped degradation at an upstream stem loop structure. To investigate this in more detail, we re-probed that blot with an oligo specific for the 5’ end of csRNA1. Intriguingly, this revealed a shorter species whose presence depends on Cbf1. We also performed the suggested 3’ RACE experiments, which determined the 3’ end of csRNA1 to be the C at position 45 of the truncated RNA. This C is directly upstream of a U stretch, comparable to the wild-type 3’ end. Secondary structure predictions indicate a weak stem loop forms upstream of position 45, which might be enough to stop processing by Cbf1. We added this information on page 11 and added Appendix Figure S5 to show the results.

Regarding stoichiometry, we do not really know how much of our purified Cbf1 protein is actually active, so we can only provide the total amount (in µg) of it that was used in the *in vitro* RNase experiment. However, we have now added molar values to the M&M section as well.

Fig 4D uses a Δcbf1 mutant without any description of cells growth. A growth curve of the mutant compared to WT background should be added.

REPLY: This is an important suggestion. The *cbf1* deletion does not influence growth when compared to the wild type. We added this experiment to Figure EV5A and mention it on page 11.

While the half-life is clearly reduced by the absence of Cbf1, the steady-state levels of csRNAs at time "0" do not seem to be significantly affected by the absence of Cbf1 in Fig 4B. This observation is contrasting with the proposed mechanism in Fig 5C where Cbf1 regulation of csRNAs by cleavage leads to ComC modulation.

REPLY: The regulon involving csRNAs is very complex and regulated by different cues. While we agree that it is somewhat surprising to see no change in steady-state levels when stability is decreased, this does not mean that there is no influence on the function of the sRNAs. One explanation would be that the cell is possibly trying to counteract the decreased stability by higher transcription of the less-functional RNAs, which results in similar steady-state levels. As speculated in our model, another option could be that Cbf1 not only affects the stability but also the function of the csRNAs. We denoted this as “Activation?” in Figure 7C. Further, we added a sentence to the discussion section comparing the possible activation by Cbf1 with eukaryotic systems (page 14). Please note that we observed something similar with several ProQ-dependent sRNAs in *Salmonella*: their stability changed, but not their steady-state levels (Smirnov et al., PNAS, 2016).

It is fair to say that there are still numerous gaps to fill in our knowledge about the regulation and molecular mechanism of the csRNAs and the whole competence regulatory loop. This will require a raft of additional experiments to determine transcription rates, which other decay

enzymes may be involved, and how the putative pairing of the csRNAs to their targets impacts their own stability. While we are generally interested in understanding the full complexity of this regulatory loop, we think it would be well beyond the scope of the current manuscript, which is focused on the *S. pneumoniae* complexome and RBPs.

Ideally, I would add a positive control related to competence in Fig 5A. For instance, Northern blots on comCDE or comAB, both of which should increase under conditions of CSP expression.

REPLY: As discussed for a similar comment by reviewer #1, we repeated this experiment and added the requested northern blot control for *comCDE*. As expected, the expression of the early competence operon *comCDE* rapidly increases 5 min into induction and then decreases again over time. This is in agreement with many past studies, as well as recent RNA-seq results (e.g. Slager et al. 2019 J Bacteriol). Note that this behavior cannot be observed without CSP stimulation (Appendix Figure S7B) and that the polycistronic *comCDE* mRNA seems to be heavily processed, revealing many different bands.

Fig 5B is not the most convincing with overlapping error bars. I would add a Northern blot showing the expression of comC mRNA or a Western showing ComC (CSP) protein. Both comC mRNA and ComC protein are expected to increase in conditions of absence of cbf1 product. This would help demonstrate the activation of the competence regulon.

REPLY: While there are overlapping error bars, it is important to note that all replicates of the competence assay show the same positive trend. It is the variance between replicates that led to the overlapping error bars. We therefore added an alternative version of Figure 5B (now Figure 7B) to Appendix Figure S7D, where we connected the data points for each of the replicates and added a sentence stating this fact on page 12.

We agree that it would be interesting to find out more about the involvement of Cbf1 and the csRNAs in the competence regulon. However, we think that a detailed RNA-centric analysis of this complex regulon warrants an independent study, whereas we would like the focus of the present paper to be on Grad-seq and the complexome and RBPs of the pneumococcus.

The section described in Fig 6 could probably be moved to right after Fig 3 when the manuscript is still demonstrating the general interest of using Grad-seq. Thereafter, the manuscript would start focusing on the cell's competence, Cbf1 and csRNAs, instead of going back to the general usefulness of the Grad-seq technique.

REPLY: See our reply to reviewer #1: we agree and have moved Figure 6 more towards the front of the paper.

Minor comments:
Please define csRNAs as there is no background on these specific sRNAs in the text.

REPLY: Thank you for noticing this; information about the csRNAs has been added to page 8.

Fig 5C shows ComC protein losing an unidentified portion before being transported outside the cell. This should be defined better.

REPLY: We have changed Figure 5C (now Figure 7C) to emphasize that ComC gets processed before it is exported.

Referee #3:

Previously, the authors' group developed gradient profiling by sequencing (Grad-seq) to analyze functional RNAs and RNA binding proteins in bacterial cells. This paper reports a successful application of this method to the study of sRNAs and their partner proteins in Gram-positive in Streptococcus pneumoniae, focusing on csRNAs that are post-transcriptional negative regulators of comC mRNA encoding the precursor protein of the competence-stimulating peptide (CSP). Through the Grad-seq analysis and tag-based pull-down assay, the authors identified the interactions between csRNAs and an exoribonuclease Cbf1. Then, they showed evidence that Cbf1 trims the 3' ends of csRNAs resulting in the stabilization of these RNAs. Furthermore, the authors showed that the transformation ability of the S. pneumoniae cells significantly increases when the cbf1 gene is disrupted. Taken together, they propose a model in which Cbf1-mediated stabilization of csRNAs contributes to a negative feedback loop that controls competence after CSP stimulation. The data seem to be convincing to support the conclusion and the paper is well written. I have no serious criticisms on this manuscript. My only comment is that the work/conclusion would be strengthened if the authors are able to pull-down csRNAs using tagged-Cbf1 protein as a bait.

REPLY: We thank the reviewer for their very supportive evaluation and suggestion for performing an RNA pull-down with Cbf1. As mentioned above, we performed UV CLIP-seq and added the results to the manuscript.

2nd Editorial Decision 27th February

Thank you for submitting your revised manuscript for our consideration, it has now been seen once more by the original referees (please see comments below). I am pleased to say that the referees find that their concerns have been satisfactorily addressed and now support publication. I would therefore like to ask you to now address some editorial issues that are listed in detail below in a final revised version. Please make any changes to the manuscript text in the attached document only using the "track changes" option. Once these minor issues are resolved, we will be happy to formally accept the manuscript for publication.

REFEREE REPORTS

Referee #1:

The authors have done a number of additional analyses and experiments to alleviate the reviewer's concerns. I was pleased to see that they went the extra mile and included CLIP data. These new results strengthen their conclusions and increase the impact of the story. I am pleased with the way the authors have addressed the comments and recommend publication.

Referee #2:

This is a revised version of a manuscript that demonstrated the usage of Grad-Seq in Streptococcus pneumoniae. The new version contains most of the suggestion we have made previously and we are now supporting publication.

Referee #3:

The work/manuscript has been substantially improved by meeting with my concerns. I have no further criticism on the revised manuscript.

2nd Revision - authors' response 3rd March 2020

The Authors have made the requested editorial changes.

3rd Editorial Decision 6th March 2020

Thank you again for submitting the final revised version of your manuscript for our consideration. I am pleased to inform you that we have now accepted it for publication in The EMBO Journal.

© European Molecular Biology Organization

EMBO PRESS

YOU MUST COMPLETE ALL CELLS WITH A PINK BACKGROUND

PLEASE NOTE THAT THIS CHECKLIST WILL BE PUBLISHED ALONGSIDE YOUR PAPER

Corresponding Author Name: Birgitta Henriques-Normark, Jörg Vogel
Journal Submitted to: The EMBO Journal
Manuscript Number: EMBOJ-2019-103852

USEFUL LINKS FOR COMPLETING THIS FORM

http://www.antibodypedia.com
http://1degreebio.org
http://www.equator-network.org/reporting-guidelines/improving-bioscience-research-repor
http://grants.nih.gov/grants/olaw/olaw.htm
http://www.mrc.ac.uk/Ourresearch/Ethicsresearchguidance/Useofanimals/index.htm
http://ClinicalTrials.gov
http://www.consort-statement.org
http://www.consort-statement.org/checklists/view/32-consort/66-title
http://www.equator-network.org/reporting-guidelines/reporting-recommendations-for-tumo
http://datadryad.org
http://figshare.com
http://www.ncbi.nlm.nih.gov/gap
http://www.ebi.ac.uk/ega
http://biomodels.net/
http://biomodels.net/miriam/
http://jjj.biochem.sun.ac.za
http://oba.od.nih.gov/biosecurity/biosecurity_documents.html
http://www.selectagents.gov/

Reporting Checklist For Life Sciences Articles (Rev. June 2017)

This checklist is used to ensure good reporting standards and to improve the reproducibility of published results. These guidelines are consistent with the Principles and Guidelines for Reporting Preclinical Research issued by the NIH in 2014. Please follow the journal's authorship guidelines in preparing your manuscript.

A- Figures
1. Data
The data shown in figures should satisfy the following conditions:

- the data were obtained and processed according to the field's best practice and are presented to reflect the results of the experiments in an accurate and unbiased manner.
- figure panels include only data points, measurements or observations that can be compared to each other in a scientifically meaningful way.
- graphs include clearly labeled error bars for independent experiments and sample sizes. Unless justified, error bars should not be shown for technical replicates.
- if n< 5, the individual data points from each experiment should be plotted and any statistical test employed should be justified
- Source Data should be included to report the data underlying graphs. Please follow the guidelines set out in the author ship guidelines on Data Presentation.

2. Captions
Each figure caption should contain the following information, for each panel where they are relevant:

- a specification of the experimental system investigated (eg cell line, species name).
- the assay(s) and method(s) used to carry out the reported observations and measurements
- an explicit mention of the biological and chemical entity(ies) that are being measured.
- an explicit mention of the biological and chemical entity(ies) that are altered/varied/perturbed in a controlled manner.

- the exact sample size (n) for each experimental group/condition, given as a number, not a range;
- a description of the sample collection allowing the reader to understand whether the samples represent technical or biological replicates (including how many animals, litters, cultures, etc.).
- a statement of how many times the experiment shown was independently replicated in the laboratory.
- definitions of statistical methods and measures:
  - common tests, such as t-test (please specify whether paired vs. unpaired), simple χ^2 tests, Wilcoxon and Mann-Whitney tests, can be unambiguously identified by name only, but more complex techniques should be described in the methods section;
  - are tests one-sided or two-sided?
  - are there adjustments for multiple comparisons?
  - exact statistical test results, e.g., P values = x but not P values < x;
  - definition of 'center values' as median or average;
  - definition of error bars as s.d. or s.e.m.

Any descriptions too long for the figure legend should be included in the methods section and/or with the source data.

In the pink boxes below, please ensure that the answers to the following questions are reported in the manuscript itself. Every question should be answered. If the question is not relevant to your research, please write NA (non applicable). We encourage you to include a specific subsection in the methods section for statistics, reagents, animal models and human subjects.

B- Statistics and general methods

	Please fill out these boxes (Do not worry if you cannot see all your text once you press return)
1.a. How was the sample size chosen to ensure adequate power to detect a pre-specified effect size?	No sample size calculation was performed.
1.b. For animal studies, include a statement about sample size estimate even if no statistical methods were used.	NA
2. Describe inclusion/exclusion criteria if samples or animals were excluded from the analysis. Were the criteria pre-established?	No data was excluded.
3. Were any steps taken to minimize the effects of subjective bias when allocating animals/samples to treatment (e.g. randomization procedure)? If yes, please describe.	No
For animal studies, include a statement about randomization even if no randomization was used.	NA
4.a. Were any steps taken to minimize the effects of subjective bias during group allocation or/and when assessing results (e.g. blinding of the investigator)? If yes please describe.	No
4.b. For animal studies, include a statement about blinding even if no blinding was done	NA
5. For every figure, are statistical tests justified as appropriate?	Yes
Do the data meet the assumptions of the tests (e.g., normal distribution)? Describe any methods used to assess it.	NA
Is there an estimate of variation within each group of data?	NA
Is the variance similar between the groups that are being statistically compared?	NA

C- Reagents

6. To show that antibodies were profiled for use in the system under study (assay and species), provide a citation, catalog number and/or clone number, supplementary information or reference to an antibody validation profile. e.g., Antibodypedia (see link list at top right), 1DegreeBio (see link list at top right).	1. Anti-FLAG antibody: Sigma, Cat# F1804, clone M2<br>2. Anti-mouse antibody: Thermo Fisher Scientific, Cat# 31430<br>3. Anti-GAPDH antibody: Immunoreactive sera to S. pneumoniae GAPDH (GenBank AAK76079) was raised in rabbit against a synthetic peptide (DPIVSSDIVGMS) corresponding to amino acids 275-286. Synthetic peptide production, immunization and validation by ELISA was done by Innovagen (Lund, Sweden), following a 41-days immunization protocol including two booster injections. It was further used in the following publications: PMID: 30139996 and PMID: 29636428.<br>4. Anti-rabbit antibody: Thermo Fisher Scientific, Cat# 31460
7. Identify the source of cell lines and report if they were recently authenticated (e.g., by STR profiling) and tested for mycoplasma contamination.	NA

* for all hyperlinks, please see the table at the top right of the document

D- Animal Models

8. Report species, strain, gender, age of animals and genetic modification status where applicable. Please detail housing and husbandry conditions and the source of animals.	NA
9. For experiments involving live vertebrates, include a statement of compliance with ethical regulations and identify the committee(s) approving the experiments.	NA
10. We recommend consulting the ARRIVE guidelines (see link list at top right) (PLoS Biol. 8(6), e1000412, 2010) to ensure that other relevant aspects of animal studies are adequately reported. See author guidelines, under 'Reporting Guidelines'. See also: NIH (see link list at top right) and MRC (see link list at top right) recommendations. Please confirm compliance.	NA

E- Human Subjects

11. Identify the committee(s) approving the study protocol.	NA
12. Include a statement confirming that informed consent was obtained from all subjects and that the experiments conformed to the principles set out in the WMA Declaration of Helsinki and the Department of Health and Human Services Belmont Report.	NA
13. For publication of patient photos, include a statement confirming that consent to publish was obtained.	NA
14. Report any restrictions on the availability (and/or on the use) of human data or samples.	NA
15. Report the clinical trial registration number (at ClinicalTrials.gov or equivalent), where applicable.	NA
16. For phase II and III randomized controlled trials, please refer to the CONSORT flow diagram (see link list at top right) and submit the CONSORT checklist (see link list at top right) with your submission. See author guidelines, under 'Reporting Guidelines'. Please confirm you have submitted this list.	NA
17. For tumor marker prognostic studies, we recommend that you follow the REMARK reporting guidelines (see link list at top right). See author guidelines, under 'Reporting Guidelines'. Please confirm you have followed these guidelines.	NA

F- Data Accessibility

18: Provide a "Data Availability" section at the end of the Materials & Methods, listing the accession codes for data generated in this study and deposited in a public database (e.g. RNA-Seq data: Gene Expression Omnibus GSE39462, Proteomics data: PRIDE PXD000208 etc.) Please refer to our author guidelines for 'Data Deposition'.<br><br>Data deposition in a public repository is mandatory for:<br>a. Protein, DNA and RNA sequences<br>b. Macromolecular structures<br>c. Crystallographic data for small molecules<br>d. Functional genomics data<br>e. Proteomics and molecular interactions	The raw data of this study have been made available in public online repositories (accession numbers see manuscript).
19. Deposition is strongly recommended for any datasets that are central and integral to the study; please consider the journal's data policy. If no structured public repository exists for a given data type, we encourage the provision of datasets in the manuscript as a Supplementary Document (see author guidelines under 'Expanded View' or in unstructured repositories such as Dryad (see link list at top right) or Figshare (see link list at top right).	The raw data of this study have been made available in public online repositories (accession numbers see manuscript). Processed data have been made available as expanded view datasets.
20. Access to human clinical and genomic datasets should be provided with as few restrictions as possible while respecting ethical obligations to the patients and relevant medical and legal issues. If practically possible and compatible with the individual consent agreement used in the study, such data should be deposited in one of the major public access-controlled repositories such as dbGAP (see link list at top right) or EGA (see link list at top right).	NA
21. Computational models that are central and integral to a study should be shared without restrictions and provided in a machine-readable form. The relevant accession numbers or links should be provided. When possible, standardized format (SBML, CellML) should be used instead of scripts (e.g. MATLAB). Authors are strongly encouraged to follow the MIRIAM guidelines (see link list at top right) and deposit their model in a public database such as Biomodels (see link list at top right) or JWS Online (see link list at top right). If computer source code is provided with the paper, it should be deposited in a public repository or included in supplementary information.	The code used in this study has been made available in public online repositories (see manuscript).

G- Dual use research of concern

22. Could your study fall under dual use research restrictions? Please check biosecurity documents (see link list at top right) and list of select agents and toxins (APHIS/CDC) (see link list at top right). According to our biosecurity guidelines, provide a statement only if it could.	No
